# Effects of palmitate on genome-wide mRNA expression and DNA methylation patterns in human pancreatic islets

**DOI:** 10.1186/1741-7015-12-103

**Published:** 2014-06-23

**Authors:** Elin Hall, Petr Volkov, Tasnim Dayeh, Karl Bacos, Tina Rönn, Marloes Dekker Nitert, Charlotte Ling

**Affiliations:** 1 Epigenetics and Diabetes Unit, Department of Clinical Sciences, Lund University Diabetes Centre, CRC, Lund University, Scania University Hospital, Malmö, Sweden; 2 School of Medicine, Royal Brisbane Clinical School, The University of Queensland, Herston, QLD 4006, Australia

**Keywords:** Palmitate, Human pancreatic islets, Type 2 diabetes, Lipotoxicity, DNA methylation, mRNA expression, Insulin secretion, Epigenetics

## Abstract

**Background:**

Circulating free fatty acids are often elevated in patients with type 2 diabetes (T2D) and obese individuals. Chronic exposure to high levels of saturated fatty acids has detrimental effects on islet function and insulin secretion. Altered gene expression and epigenetics may contribute to T2D and obesity. However, there is limited information on whether fatty acids alter the genome-wide transcriptome profile in conjunction with DNA methylation patterns in human pancreatic islets. To dissect the molecular mechanisms linking lipotoxicity to impaired insulin secretion, we investigated the effects of a 48 h palmitate treatment *in vitro* on genome-wide mRNA expression and DNA methylation patterns in human pancreatic islets.

**Methods:**

Genome-wide mRNA expression was analyzed using Affymetrix GeneChip® Human Gene 1.0 ST whole transcript-based array (n = 13) and genome-wide DNA methylation was analyzed using Infinium HumanMethylation450K BeadChip (n = 13) in human pancreatic islets exposed to palmitate or control media for 48 h. A non-parametric paired Wilcoxon statistical test was used to analyze mRNA expression. Apoptosis was measured using Apo-ONE® Homogeneous Caspase-3/7 Assay (n = 4).

**Results:**

While glucose-stimulated insulin secretion was decreased, there was no significant effect on apoptosis in human islets exposed to palmitate. We identified 1,860 differentially expressed genes in palmitate-treated human islets. These include candidate genes for T2D, such as *TCF7L2, GLIS3, HNF1B* and *SLC30A8*. Additionally, genes in glycolysis/gluconeogenesis, pyruvate metabolism, fatty acid metabolism, glutathione metabolism and one carbon pool by folate were differentially expressed in palmitate-treated human islets. Palmitate treatment altered the global DNA methylation level and DNA methylation levels of CpG island shelves and shores, 5′UTR, 3′UTR and gene body regions in human islets. Moreover, 290 genes with differential expression had a corresponding change in DNA methylation, for example, *TCF7L2* and *GLIS3*. Importantly, out of the genes differentially expressed due to palmitate treatment in human islets, 67 were also associated with BMI and 37 were differentially expressed in islets from T2D patients.

**Conclusion:**

Our study demonstrates that palmitate treatment of human pancreatic islets gives rise to epigenetic modifications that together with altered gene expression may contribute to impaired insulin secretion and T2D.

## Background

The risk of developing type 2 diabetes (T2D) is influenced by both genetic and environmental factors. While genome-wide association studies (GWAS) have identified more than 60 single nucleotide polymorphisms (SNPs) associated with an increased risk for T2D [1,2], obesity, physical inactivity and ageing represent non-genetic risk factors for the disease. Recent studies suggest that epigenetic factors, such as DNA methylation, play a role in the pathogenesis of T2D [3-11]. Nevertheless, genome-wide human epigenetic studies linking altered DNA methylation to diabetes remain scarce. In mammalian cells DNA methylation mainly occurs at the cytosine of CpG dinucleotides. Methylated CpG sites can alter transcriptional activity by interfering with binding of transcription factors in promoter regions or by recruiting methyl binding proteins which in turn may recruit histone deacethylases and transcriptional co-repressors [3]. Increased DNA methylation of beta-cell specific genes, such as *PDX-1* and *INS,* correlates negatively with the expression of respective genes in pancreatic islets from T2D patients [4,5].

Plasma levels of free fatty acids are often elevated in T2D patients and in obese individuals [12,13]. Chronic exposure to high levels of fatty acids has negative effects on beta-cell function [12,13]. The severity of this effect depends on the length and saturation of fatty acids. Long chain saturated fatty acids, for example, palmitate and stearate, are reportedly more cytotoxic than the long chain unsaturated fatty acid oleate [14-16], and long term treatment (≥48 h) with palmitate reduces glucose-stimulated insulin secretion in rodent islets and clonal beta-cells [17,18]. Moreover, prolonged exposure to non-esterified fatty acids *in vivo* also resulted in impaired islets function and decreased glucose-stimulated insulin secretion in humans [19,20]. Additionally, transcriptome analyses of clonal beta-cells revealed differences in the gene expression pattern in cells treated with high palmitate concentrations. Specifically, palmitate exposure altered the expression of genes with a role in fatty acid metabolism and steroid biosynthesis [21,22]. In clonal beta-cells, palmitate exposure also altered histone modifications [22]. As most of the cell types in pancreatic islets affect whole body energy homeostasis [23], it is essential to also study the impact of fatty acids on intact human islets. However, while some studies have analyzed expression of specific genes in human islets exposed to palmitate [24-27], to our knowledge no previous study has analyzed the genome-wide expression profile in palmitate-treated human islets of more than five human donors [28,29]. Moreover, whether the genome-wide DNA methylation pattern is affected by fatty acids in human islets remains unknown.

The aim of this study was therefore to investigate if treatment with palmitate for 48 h affects genome-wide mRNA expression and DNA methylation patterns in human pancreatic islets and, consequently influences glucose-stimulated insulin secretion and/or apoptosis. To validate our *in vitro* findings, we related genome-wide gene expression in human islets to BMI in non-diabetic individuals and to T2D in a case-control cohort.

## Methods

### Human pancreatic islets

Pancreatic islets from 13 donors were included in the genome-wide RNA and DNA methylation array analyses. While pancreatic islets from eight donors were included in both the mRNA array analysis and the DNA methylation array analysis, pancreatic islets from five donors were unique for each array (Table 1 and Additional file 1: Table S1). The impact of body mass index (BMI) on gene expression was studied in pancreatic islets from 87 non-diabetic donors (53 males and 34 females, BMI ranged between 17.6 to 40.1 kg/m^2^, mean BMI = 25.8 ± 3.4 kg/m^2^, age = 56.7 ± 10.5 years). The impact of T2D on gene expression was studied in pancreatic islets from 15 donors (10 males and 5 females, age = 59.5 ± 10.7 years and mean BMI = 28.3 ± 4.7 kg/m^2^) diagnosed with T2D and 34 non-diabetic donors (22 males and 12 females, age = 56.0 ± 9.0 years and mean BMI = 28.3 ± 4.7 kg/m^2^) with an HbA1c below 6.0%. Informed consent for organ donation for medical research was obtained from pancreatic donors or their relatives in accordance with the approval by the regional ethics committee in Lund, Sweden (Dnr 173/2007). This study was performed in agreement with the Helsinki Declaration.

**Table 1:** Characteristics of human pancreatic donors included in the mRNA expression array analysis

n (male/female)	13 (7/6)
Age (years)	55 ± 14
BMI (kg/m^2^)	25.5 ± 4.3
HbA1c* (%)	5.6 ± 0.9
HbA1c* (mmol/mol)	47.6 ± 9.2

Data are expressed as mean ± SD. BMI, Body Mass Index.

*Data available for eight donors (five males and three females).

Human pancreatic islets were prepared by collagenase digestion and density gradient purification. The islet purity was 80% ± 2.5%, as assessed by the ratio of expression of islet (*INS, GCG,* and *SST)* and non-islet specific (*AMY2A, PNLIP, CTRC*) genes.

### Preparation of medium containing palmitate

First, a stock solution of 10 mM palmitate and 10% fatty acid free BSA was created. A total of 128 mg palmitate was dissolved in 50 ml 99% ethanol and then 60 μl 10 M NaOH was added. The solution was vacuum-dried and then resolved in 25 ml H_2_O during heating. Next, 6 g of fatty acid free BSA was dissolved in 24 ml H_2_O and then 25 ml was taken and mixed with the 25 ml palmitate solution. The stock solution was then diluted to a final concentration of 1 mM palmitate and 1 weight % BSA (corresponding to 0.15 mM BSA) in the CMRL 1066 medium (ICN Biomedicals, Costa Mesa, CA, USA) supplemented with 10 mM nicotinamide (Sigma-Aldrich, Sweden, Stockholm), 10 mM HEPES buffer (GIBCO, BRL, Gaithersburg, MD, USA), 0.25 μg/ml fungizone (GIBCO), 50 μg/ml gentamicin, 2 mM L-glutamine (GIBCO), 10 μg/ml Ciprofloxacin (Bayer Healthcare, Leverkusen, Germany), 10% (v/v) heat-inactivated human serum and 5.56 mM glucose. The molar (mmol/l) ratio of palmitate/BSA concentrations was 6.6:1 in the culture medium.

### Palmitate treatment

**Figure 1:**
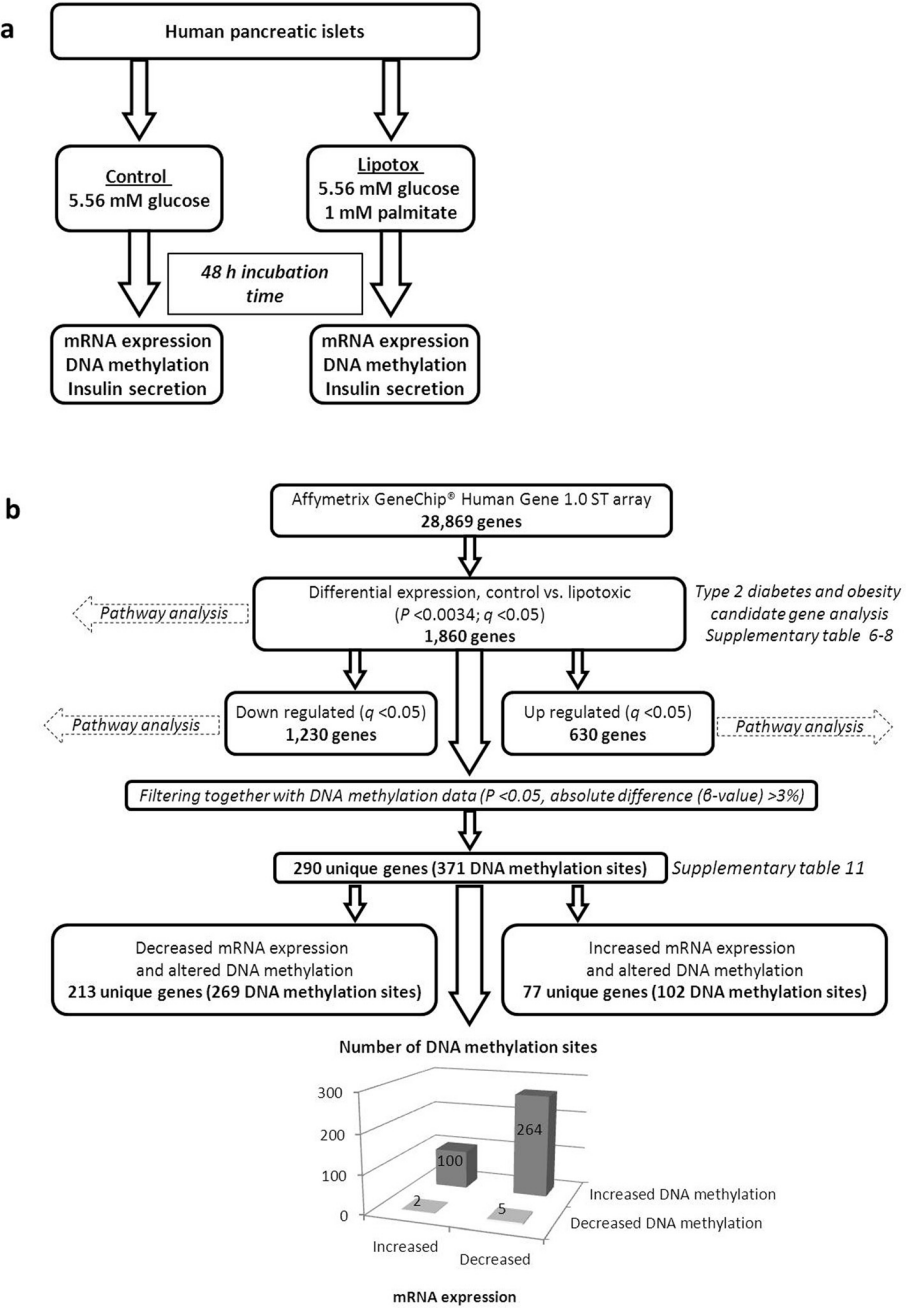
**Study design and work flow.** Study design for the lipotoxicity study in human pancreatic islets is presented in panel **a** Work flow for the analysis of mRNA expression data in combination with DNA methylation data in human pancreatic islets exposed to palmitate is presented in panel **b**.

To study the impact of palmitate-induced lipotoxicity on human islets, approximately 1,000 islets from each donor (n = 13) were cultured for 48 h in CMRL 1066 medium (including 5.56 mM glucose) either with (lipotox) or without (control) 1 mM palmitate conjugated with 1% BSA (corresponding to 0.15 mM BSA) (Figure 1a). The same treatment time and palmitate/BSA ratio have been used in previous studies examining the impact of lipotoxicity on islet function and was therefore selected in the present study [22,30]. Circulating non-esterified fatty acid levels have been reported to range between 0.59 to 0.83 mM for overweight, non-diabetic individuals (BMI approximately 26 kg/m^2^) and between 0.69 to 0.975 mM for overweight, diabetic individuals (BMI of approximately 29 kg/m^2^) [31]. The 1 mM palmitate used in the current study, which is close to the upper limit of the reference range, mimics the levels reported in overweight/obese individuals with diabetes. After 48 h DNA and RNA were extracted, glucose-stimulated insulin secretion was analyzed and/or apoptosis assays were performed.

### RNA and DNA isolation

DNA and RNA were extracted from the human pancreatic islets using the AllPrep DNA/RNA kit (Qiagen, Hilden, Germany) according to the manufacturer’s instructions. RNA quantity and quality were assessed by Nanodrop (Nanodrop, Wilmington, DE, USA). The 260/280 ratios of all samples were between 1.98 and 2.16. The integrity and quality of the RNA was assessed using the Bioanalyzer (Agilent Technologies, Santa Clara, CA, USA). All RNA integrity number (RIN) values were ≥7.4.

### Microarray mRNA expression analysis

The Affymetrix GeneChip® Human Gene 1.0 ST whole transcript-based array (Affymetrix, Santa Clara, CA, USA) covering 28,869 genes was used to analyze mRNA expression (Figure 1b) in pancreatic islets from 13 human donors (Table 1) exposed to palmitate or control conditions (in total 26 samples) and in pancreatic islets from 87 non-diabetic donors as well as from diabetic and non-diabetic donors, according to the manufacturer’s recommendations. The Oligo package from Bioconductor was used to compute Robust Multichip Average expression measures [32].

### Genome-wide DNA methylation analysis

A total of 500 ng genomic DNA from human pancreatic islets of 13 donors (Additional file 1: Table S1) exposed to palmitate or control conditions (in total 26 samples) was bisulfite-converted with the EZ DNA methylation kit (Zymo Research Corporation, Irvine, CA, USA). DNA methylation was analyzed by using the Infinium HumanMethylation450K BeadChip (Illumina, San Diego, CA, USA) which contains 485,577 probes and covers 99% of all RefSeq genes [33]. Bisulfite converted DNA was used to analyze DNA methylation with the Infinium® assay according to the standard Infinium HD Assay Methylation Protocol (Part # 15019519, Illumina). The Infinium HumanMethylation450K BeadChips were then imaged with the Illumina iScan. The raw methylation score for each CpG site, which is represented as β-value, was calculated using the GenomeStudio® methylation module software. The β-values were calculated as (β = intensity of the methylated allele (M)/(intensity of the Unmethylated allele (U) + intensity of the Methylated allele (M) + 100)). All samples passed GenomeStudio® quality control steps based on built-in control probes for staining, hybridization, extension and specificity, and displayed high quality bisulfite conversion efficiency with an intensity signal above 4,000 [34]. Probes were filtered away from further analysis based on a mean detection *P*-value >0.01. After quality control analysis, DNA methylation data were obtained for 483,844 probes. β-values were then converted to M-values (M = log2 (β/(1 - β))) for further bioinformatic and statistical analyses of the methylation data [35]. Background and quantile normalization was performed using the lumi package from Bioconductor [36]. Background correction was performed by subtracting the median M-value of the 600 built-in negative controls and methylation data were further normalized using quantile normalization [37]. ComBat was used to adjust for batch effects between arrays [38]. A linear regression model was used to identify differences in DNA methylation between control and palmitate-treated islets in a paired fashion as described elsewhere [39]. As β-values are biologically easier to interpret, M-values were reconverted to β-values when describing the DNA methylation results. The DNA methylation probes on the Infinium HumanMethylation450K BeadChip have been annotated to different genomic regions depending on their location in relation to a gene or a CpG island [33].

### KEGG pathway analysis

Kyoto Encyclopedia of Genes and Genomes (KEGG) pathway analysis of expression data was performed with the online tool WebGestalt [40,41] (accessed 27 March 2012 and 12 February 2014). For the pathway analysis of mRNA expression data, Affymetrix probe IDs were used to identify unique genes and Affymetrix GeneChip® Human Gene 1.0 ST genes were used as background in this analysis. For the pathway analysis of the DNA methylation data, the gene symbol was used to identify unique genes and the human genome was used as background in this analysis. The Benjamini and Hochberg method was used to correct *P*-values for multiple testing.

### Glucose-stimulated insulin secretion

Glucose-stimulated insulin secretion was analyzed in control and palmitate-treated human islets from nine donors. After 48 h culture in control or palmitate-containing medium, 10 replicates of 10 human islets per culture condition (control and palmitate-treated) and donor were pre-incubated in HEPES-balanced salt solution (HBSS) containing (in mM) 114 NaCl, 4.7 KCl, 1.2 KH_2_PO_4_, 1.16 MgSO_4_, 20 HEPES, 25.5 NaHCO_3_, 2.5 CaCl_2_ at pH 7.2 with 0.575 BSA and 3.3 mM glucose (1.65 mM glucose for one sample) for 1 h at 37°C. Thereafter, for each donor, glucose was added to five of the replicates to a final concentration of 16.7 mM glucose (15.05 mM glucose for one sample) to study glucose-stimulated insulin secretion and the other five replicates were kept in 3.3 mM glucose to study basal insulin secretion and the incubation was continued for one more hour. The supernatant was immediately removed and the insulin concentration in the medium was measured by radioimmunoassay (RIA) (Millipore, Uppsala, Sweden).

### Assessment of apoptosis in human pancreatic islets

Apoptosis was measured in islets from four human donors with the Apo-ONE® Homogeneous Caspase-3/7 Assay (Promega, Madison, WI, USA) as described elsewhere [42]. The assay contains proflourescent rhodamin 110 (Z-DEVD-R110) which serves as a substrate for both Caspase-3 and -7. Upon lysis of cells, the available Caspase -3/-7 in the sample will cleave Z-DEVD-R110 to fluorescent rhodamine 110, which is then measured. Subsequently, the assay measures the combined activity of Caspase-3 and -7. After 48 h incubation in control or palmitate medium, triplicates of 20 human pancreatic islets each were handpicked from each culture condition, washed and transferred to a plate containing HBSS. After 1.5 h, fluorescence was measured with a Tecan Infinite M200pro plate reader (Tecan Group Ltd., Männedorf, Switzerland) to determine the Caspase-3/7 activity.

### Statistics

A non-parametric paired test (Wilcoxon) was used to identify differences in mRNA expression between control and palmitate-treated human islets. A False Discovery Rate (FDR) analysis was performed to correct for multiple testing in the mRNA expression data. Genes exhibiting differential expression with a FDR below 5% (*q* <0.05) were considered significant. To find associations between BMI and gene expression in human islets, a linear regression model was used including age, gender, HbA1c, islet purity and days of culture as covariates. To identify differences in gene expression between T2D and non-diabetic islets a linear regression model was used including gender, BMI, age, islet purity and days of culture as covariates. Data are presented as mean ± standard error of mean (sem), unless stated otherwise.

## Results

### Impaired insulin secretion in human islets exposed to palmitate

**Figure 2:**
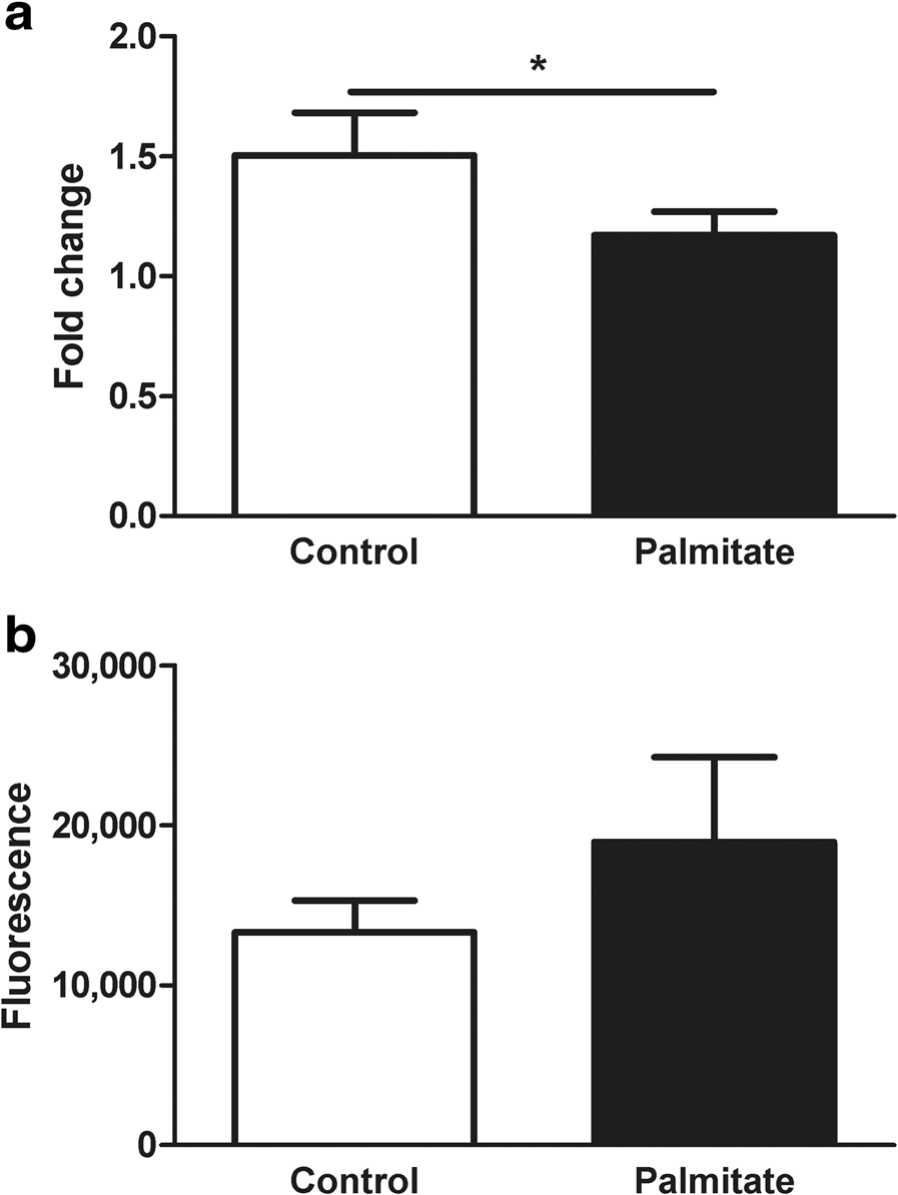
**Impact of palmitate treatment on insulin secretion and apoptosis in human pancreatic islets. a)** Glucose**-**stimulated insulin secretion represented as fold change of insulin secretion (insulin secretion at high glucose levels/insulin secretion at low glucose levels) from human islets (n = 9) exposed to palmitate or control treatment for 48 h. A Wilcoxon one-tailed test was used. *P ≤0.05. **b)** Combined activity of Caspase-3/-7 as a measure of apoptosis in human islets (n = 4) exposed to palmitate or control treatment for 48 h. A Wilcoxon test was performed.

To investigate the physiological response to 1 mM palmitate treatment for 48 h, we measured glucose-stimulated insulin secretion in human islets cultured under control (5.56 mM glucose) or lipotoxic (5.56 mM glucose and 1 mM palmitate) conditions. We found decreased glucose-stimulated insulin secretion measured as fold change (insulin secretion at high glucose levels/insulin secretion at low glucose levels) in the palmitate-treated compared with control-treated human islets (Figure 2a). We also evaluated the effect of the palmitate treatment on apoptosis in human islets by measuring the combined activity of Caspase-3 and -7. Palmitate treatment did not alter islet cell apoptosis rates (*P* = 0.62, Figure 2b).

### Palmitate exposure influences mRNA expression in human islets

To study the impact of palmitate-induced lipotoxicity on gene expression in human islets, mRNA expression array data were generated for pancreatic islets from 13 donors cultured in control or lipotoxic conditions (Figure 1a). The characteristics of these 13 donors are described in Table 1. We identified 1,860 individual genes that were differentially expressed in human islets exposed to palmitate compared with the control condition after correction for multiple testing using a FDR below 5% (*q* <0.05) (Additional file 2: Table S2). Out of these 1,860 genes, 1,230 were down-regulated and 630 genes were up-regulated due to palmitate treatment. The work flow for the mRNA expression data can be viewed in Figure 1b.

**Figure 3:**
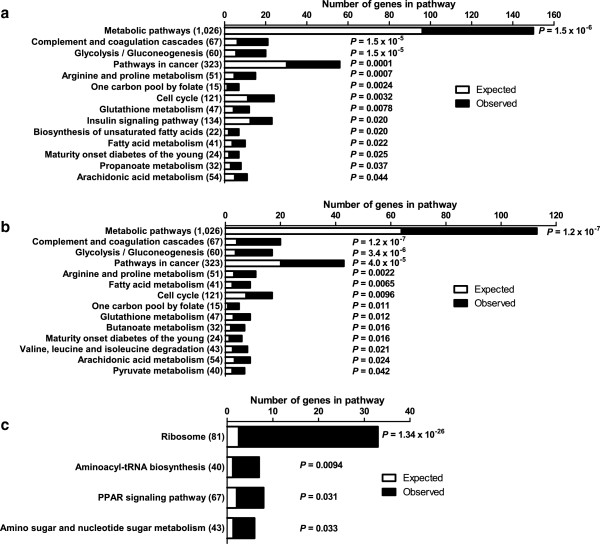
**Gene set analysis of differentially expressed genes in human islets exposed to palmitate.** Results of KEGG pathway analysis using **a)** all differentially expressed genes, **b)** down-regulated genes only and **c)** up-regulated genes only in human islets exposed to palmitate. Numbers in brackets indicate the total number of genes in the corresponding pathway.

**Figure 4:**
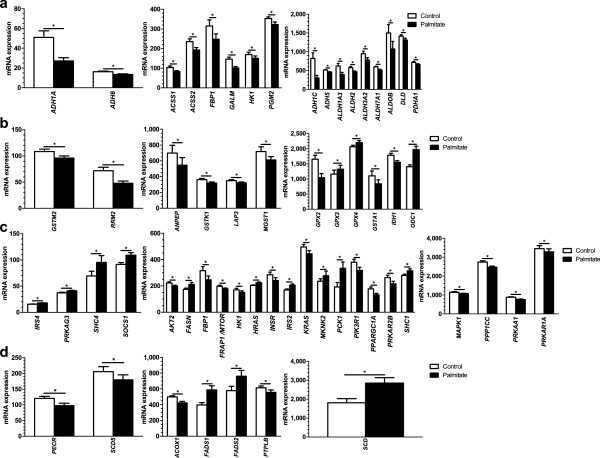
**Differential mRNA expression of genes in enriched KEGG pathways in palmitate-exposed human islets. a)** Differentially down-regulated genes in the glycolysis/gluconeogenesis pathway. **b)** Differentially expressed genes in the glutathione metabolism pathway. **c)** Differentially expressed genes in the insulin signaling pathway. **d)** Differentially expressed genes in the biosynthesis of unsaturated fatty acids pathway. All data are presented as mean ± sem. *q <0.05.

To test if genes in certain biological pathways were enriched among the differentially expressed genes in palmitate-treated human islets, KEGG pathway analyses were performed using WebGestalt. Pathway analyses were performed using either the list of all differentially expressed genes (*q* <0.05) or by dividing the genes based on down- or up-regulation in islets exposed to palmitate. A selection of the enriched pathways (*P*_*adjusted*_ <0.05) can be found in Figure 3 and all enriched pathways of possible relevance for lipotoxicity in human islets can be found in Additional file 3: Table S3, Additional file 4: Tables S4 and Additional file 5: Table S5. The metabolic pathways was the top KEGG pathway with down-regulated genes (Figure 3b and Additional file 4: Table S4) and it includes several genes encoding proteins involved in oxidative phosphorylation. Additionally, the glycolysis/gluconeogenesis (Figures 3a, b, 4a and Additional file 3: Table S3 and Additional file 4: Table S4), fatty acid metabolism, glutathione metabolism (Figures 3a, b, 4b and Additional file 3: Table S3 and Additional file 4: Table S4) and pyruvate metabolism (Figure 3b and Additional file 4: Table S4) pathways were enriched in human islets exposed to palmitate. Moreover, there was an enrichment of genes involved in the insulin signaling pathway (Figures 3a, 4c and Additional file 3: Table S3) and the biosynthesis of unsaturated fatty acids pathway (Figures 3a, 4d and Additional file 3: Table S3). Interestingly, the “one carbon pool by folate” was also enriched in the KEGG pathway analysis (Figure 3a, b and Additional file 3: Table S3 and Additional file 4: Table S4).

**Figure 5:**
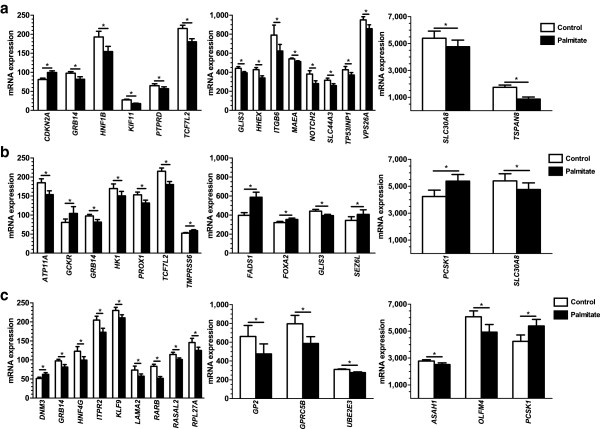
**Impact of palmitate treatment on gene expression of candidate genes for T2D, related traits and obesity in human islets.** Differentially expressed candidate genes (*q* <0.05) for **a)** T2D, **b)** T2D related traits and **c)** obesity. Figures are divided into different expression levels. All data are presented as mean ± sem. *q <0.05. T2D, type 2 diabetes.

We further examined if candidate genes associated with either T2D, T2D-related traits or obesity in previous GWAS were differentially expressed in human islets exposed to palmitate. Candidate gene lists were retrieved using the online GWAS SNP library [43,44] (accessed on 22 August 2012). Among these gene lists, we found 16 candidate genes for T2D out of a total of 86 genes (Figure 5a and Additional file 6: Table S6), 13 T2D-related trait genes out of a total of 76 (Figure 5b and Additional file 7: Table S7) and 15 candidate genes for obesity out of a total of 127 (Figure 5c and Additional file 8: Table S8) that were differentially expressed palmitate-treated islets. However, since some candidate genes are associated with more than one trait, the differentially expressed candidate genes correspond to 38 unique genes out of a total of 262 genes.

### Impact of palmitate on DNA methylation in human islets

**Figure 6:**
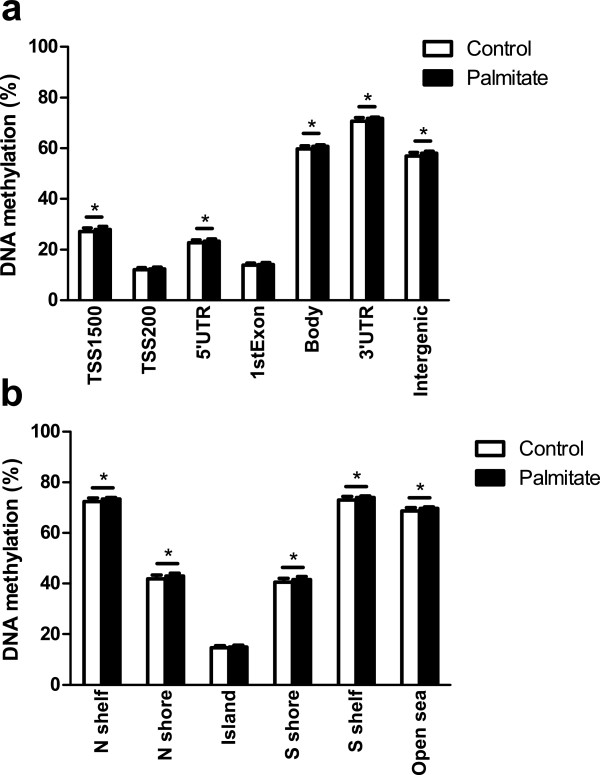
**Impact of palmitate treatment on global DNA methylation in human islets.** Average DNA methylation levels of **a)** gene regions and **b)** CpG island regions in control and lipotoxic treated human islets. All data are presented as mean ± sem. *q <0.05.

To estimate the global DNA methylation in human islets, we calculated the average level of DNA methylation for all analyzed CpG sites on the Infinium HumanMethylation450K BeadChip array. The average level of genome-wide DNA methylation was slightly, but significantly, higher in palmitate-treated compared to control islets (44.9 ± 0.8 vs. 43.9 ± 1.2, *P* = 0.002). The analyzed DNA methylation sites on the array have been annotated to different gene and CpG island regions [33]. The annotated gene regions include TSS1500, TSS200, 5′UTR, 1^st^ exon, gene body, 3′UTR and intergenic regions. Annotations were also made according to the location of the DNA methylation sites in relation to CpG islands, as previously defined [33]. The 2 kb sequences, directly up- and downstream of CpG islands are called the northern and southern shore, respectively. The 2 kb sequences directly adjacent to the shores are called the northern and southern shelves. DNA methylation sites outside the CpG island regions are annotated as “open sea”. We then tested if palmitate exposure affects the average level of DNA methylation for any of these gene regions in human islets. We found an increase in average DNA methylation in the palmitate-treated islets for all gene and CpG island regions except TSS200, 1st Exon and CpG islands (Figure 6 and Additional file 9: Table S9).

We next evaluated if palmitate also affects the level of DNA methylation of individual CpG sites in human islets. Genome-wide DNA methylation array data were successfully generated for 483,844 sites in islets of 13 donors. Palmitate exposure changed the degree of DNA methylation of 46,977 sites at *P* <0.05, which is almost double the expected number with *P* <0.05 and significantly more than expected based on a chi-squared test (*P* <0.0001, Additional file 10: Table S10). However, no individual methylation site had *q* <0.05 based on a FDR analysis and the lowest *P*-value was 5.7 × 10^−6^. Out of those, 4,690 sites had an absolute difference in DNA methylation greater than 3% in palmitate-treated versus control islets. This cut-off was set to increase the biological relevance of the results. Among the 4,690 sites with an absolute difference in methylation greater than 3% and *P* <0.05, 4,561 sites displayed increased DNA methylation due to palmitate treatment, corresponding to 2,753 unique genes and 1,429 intergenic sites. Moreover, 129 sites showed decreased DNA methylation due to palmitate exposure out of which 99 were located in 94 unique genes, and 30 were intergenic sites. The fold change for the 46,977 differentially methylated DNA methylation sites (*P* <0.05), calculated as DNA methylation of palmitate-treated islets/DNA methylation of control treated islets, ranged from 0.54 to 1.84. This corresponds to changes in DNA methylation from a 46% decrease to an 84% increase.

### Overlapping changes in mRNA expression and DNA methylation in palmitate-treated human islets

Epigenetic modifications may regulate mRNA expression [3-5] and we therefore tested if any of the genes that exhibit differential mRNA expression also exhibit differential DNA methylation in islets exposed to palmitate. Significant mRNA expression data (*q* <0.05) were merged with DNA methylation sites with *P* <0.05 and an absolute difference in DNA methylation ≥3%. We found 290 individual genes with differential mRNA expression (*q* <0.05) and a corresponding change in DNA methylation (in total, 371 DNA methylation sites) (Figure 1b and Additional file 11: Table S11). Out of these 290 genes, 213 had decreased mRNA expression together with altered DNA methylation (269 DNA methylation sites, whereof 264 sites had increased and 5 sites had decreased DNA methylation) in response to palmitate treatment. Additionally, 77 unique genes had increased mRNA expression together with altered DNA methylation (102 DNA methylation sites, whereof 2 sites had decreased and 100 sites had increased DNA methylation) (Figure 1b). Furthermore, for some of the candidate genes for T2D, T2D related traits and obesity identified by GWAS, we found both differential mRNA expression and changes in DNA methylation in islets exposed to palmitate, for example, *TCF7L2* and *GLIS3* show decreased expression and increased DNA methylation (Additional file 6: Table S6, Additional file 7: Table S7 and Additional file 8: Table S8).

A KEGG pathway analysis was performed using WebGestalt to test if genes in biological pathways found in the mRNA expression pathway analysis (Additional file 3: Table S3, Additional file 4: Table S4 and Additional file 5: Table S5) also were enriched among the differentially methylated genes in palmitate-treated human islets. The pathway analysis was performed using a list of all differentially methylated genes (*P* <0.05) (Additional file 10: Table S10). All 17 pathways with enrichment for both DNA methylation and gene expression are presented in Additional file 12: Figure S1 and Additional file 13: Table S12.

The Infinium HumanMethylation450K BeadChip array has been reported to have probes with possible cross reactivity to other locations in the genome than their intended match [45]. Importantly, none of our reported probes with *P* <0.05 have a perfect match to other locations in the genome. Furthermore, only 13 probes have a near-perfect match (Additional file 14: Table S13).

### Impact of BMI and T2D on gene expression in human islets

**Figure 7:**
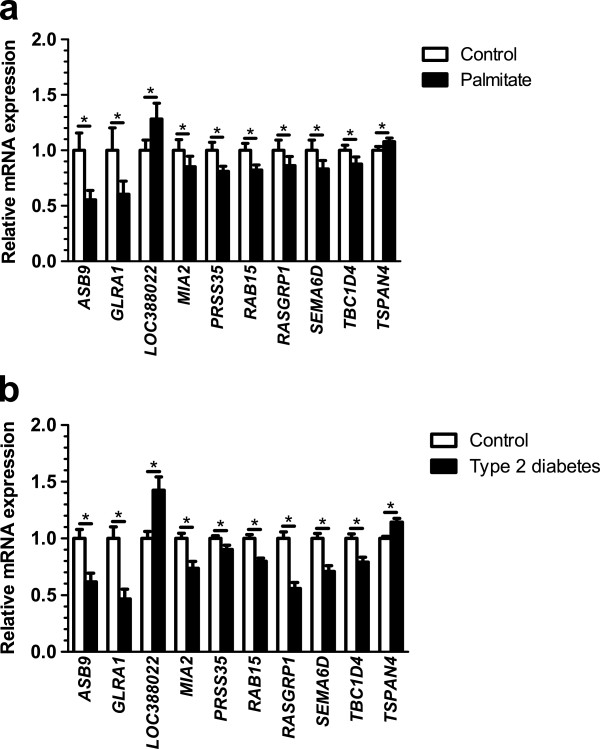
**Top 10 genes showing differential expression in human islets due to both palmitate exposure and T2D. a)** Relative mRNA expression of the top 10 significant genes, in human islets exposed to control or palmitate treatment, overlapping with differentially expressed genes in human islets from donors with or without T2D. **b**) Relative mRNA expression of the top 10 significant genes, in human islets from donors with or without T2D, overlapping with differentially expressed genes in human islets exposed to control or palmitate treatment. Data are presented as mean ± sem. *P ≤0.05. T2D, type 2 diabetes.

Since it has been shown that T2D patients and obese individuals have elevated levels of free fatty acids [12,13,46,47], we finally tested if increased BMI and/or T2D also affect islet expression in the same direction as any of the 1,860 genes that exhibit differential expression in human islets exposed to lipotoxicity for 48 h *in vitro*. The impact of BMI on expression of these 1,860 genes was examined in human islets from 87 non-diabetic donors with BMI spanning between 17.6 to 40.1 kg/m^2^. BMI was associated with differential expression of 67 of the 1,860 genes (Additional file 15: Table S14). The impact of T2D was examined in pancreatic islets from 15 donors with T2D and 34 non-diabetic donors. We found 37 genes differentially expressed (*P* <0.05) in islets from T2D versus non-diabetic donors overlapping with the 1,860 genes in the palmitate-exposed islets (Additional file 16: Table S15). The data for three of these genes (that is, *CDKN1A, IL1RL2, TNFRSF10B*) have been reported previously [48] and are hence not reported here. The top 10 genes showing differential expression in human islets due to both palmitate exposure and T2D are presented in Figure 7. Moreover, five genes, that is, *RASGRP1, MIA2, CDKN1A, TNFRSF103* and *RAB7L1*, were present among both the BMI- and T2D-associated genes.

## Discussion

This study shows that palmitate exposure alters mRNA expression genome-wide in human islets in parallel with impaired insulin secretion, a defect often seen in T2D patients. Several genes with altered expression in palmitate-treated human islets also exhibited differential expression in islets from patients with T2D. We also demonstrate for the first time that the genome-wide DNA methylation pattern in human islets was affected by palmitate treatment. Several genomic regions had significantly higher global DNA methylation levels in the palmitate-treated islets compared with control islets, although these differences were generally small. This may be the result of the relatively short treatment (48 h) and that DNA methylation changes of a larger magnitude may require longer exposure to hyperlipidemia, a condition seen in many T2D patients. Also, since T2D is known to be a polygenic disease, it is possible that a combination of several modest changes in DNA methylation might have a combined larger effect which together could contribute to the pathogenesis of the disease. In support of this hypothesis, previous studies have shown relatively modest differences of DNA methylation in non-cancerous tissues and cell types, ranging from 0.13% to 11% [9,49,50]. However, even an absolute change of only a few percent units can represent a large difference in relative terms, as evident by the findings in our study where the fold change of DNA methylation between the treatment groups (palmitate treatment/control treatment) ranged from 0.54 to 1.84. This is in line with data from a recent study, where we found differential DNA methylation of 3,116 CpG sites in human pancreatic islets from subjects with T2D compared with non-diabetic controls with a fold change ranging from 0.58 to 1.61 when dividing the degree of methylation in diabetics with that in controls [48].

We also identified many genes with a difference in mRNA expression and a corresponding change in DNA methylation. This could suggest that altered DNA methylation influences the expression of the corresponding genes. Indeed, we have previously shown that increased DNA methylation reduces the transcriptional activity in functional *in vitro* studies [5,8]. Interestingly, here we find decreased expression in parallel with increased DNA methylation of several candidate genes for T2D, such as *TCF7L2* and *GLIS3*[51], in palmitate-treated human islets, suggesting that lipid-induced epigenetic modifications may affect the risk for diabetes. The fact that many of the up-regulated genes have corresponding increased DNA methylation could be due to the location of these CpG sites in the gene body. Indeed, DNA methylation of the gene body has been demonstrated to have a positive effect on gene expression [52]. The gene regions with differential gene expression but without any change in DNA methylation could be targets for other forms of transcriptional regulation, such as histone modifications and/or altered activation by transcription factors. Also, genetic and epigenetic variation may interact to affect gene expression and subsequently contribute to the development of complex metabolic disease, such as obesity and T2D. Indeed, it has previously been shown that SNPs that introduce or remove a CpG site, so called CpG-SNPs, can influence the expression of target genes by interfering with certain proteins [53]. Moreover, we recently showed that approximately 50% of SNPs associated with T2D are CpG-SNPs, which affect the degree of DNA methylation in the SNP site as well as gene expression and alternative splicing events in human pancreatic islets [7]. It has been hypothesized that since DNA methylation can affect the regulation of splicing, CpG-SNPs can possibly affect alternative splicing events [54].

There is an increased risk for obesity and T2D among children with obese and/or diabetic parents [55,56]. Additionally, rodent studies demonstrate that an altered intrauterine environment gives rise to epigenetic changes, which later in life can predispose the offspring to impaired metabolism and T2D [57-59]. These data suggest that epigenetic modifications contribute to the pathogenesis of T2D. Based on the results from our study, we speculate that early exposure to palmitate may affect the epigenetic patterns of genes which are known to affect the risk of T2D. This may increase the risk of disease later in life. However, we cannot exclude that epigenetic changes seen in patients with T2D are secondary to the disease [4,5,48,60,61].

Our human insulin secretion data are in concordance with previous rodent studies, where palmitate treatment was found to lower glucose-stimulated insulin secretion in rodent pancreatic islets [17,18]. A tight coupling of glycolysis to mitochondrial respiration and ATP production is essential for proper beta-cell function and glucose-stimulated insulin secretion. Palmitate treatment of human islets resulted in altered expression of individual metabolic genes as well as of genes in metabolic pathways such as glycolysis/gluconeogenesis, pyruvate metabolism and biosynthesis of unsaturated fatty acids. Additionally, several down-regulated genes in the enriched metabolic pathways encode proteins which are part of the respiratory chain, for example, *NDUFA4*, *NDUFB5*, *NDUFS1*, *NDUFS2*, *SDHA* and *UQCRB*. Decreased expression of these genes may contribute to decreased oxidative phosphorylation and subsequently decreased ATP production and insulin secretion in islets exposed to lipotoxicity. Indeed, our previous study showed that decreased expression of genes involved in oxidative phosphorylation results in impaired insulin secretion [62].

While some studies have found decreased beta-cell number in T2D islets, others do not find an altered cell composition in diabetic islets [10,63-65]. In the present study, palmitate had no significant effect on apoptosis in human islets and it is hence unlikely that the beta-cell number is significantly decreased. As the majority of cell types in human islets have important effects on whole body glucose homeostasis [23], it is physiologically warranted to study both whole human islets and cell-lines representing the individual cell types in the pancreatic islets.

Additionally, the insulin signaling pathway was significantly enriched when performing a pathway analysis on all the significant expression data, including both up- and down-regulated genes. Interestingly, this pathway was also enriched when performing a pathway analysis on the differentially methylated genes. Previous studies have shown that insulin signaling contributes in the regulation of beta-cell mass and apoptosis as well as insulin synthesis and secretion [66] and here we show that this pathway is affected by palmitate treatment in human islets. This in turn could potentially affect insulin secretion in these islets. *PPARGC1A* (encoding PGC1α) is a part of the insulin signaling pathway and its expression was reduced in human islets exposed to palmitate. We have previously shown that *PPARGC1A* expression is decreased in islets from T2D patients compared to non-diabetics, and *PPARGC1A* expression correlated positively with insulin secretion in human islets [6]. *PPARGC1A* encodes a transcriptional co-activator of mitochondrial genes involved in oxidative phosphorylation and silencing of *PPARGC1A* in human islets results in decreased insulin secretion [6]. Furthermore, *SCD* (encoding for stearoyl-CoA desaturase (delta-9-desaturase)) was up-regulated in human islets due to palmitate treatment. *SCD* is a component of the biosynthesis of unsaturated fatty acids pathway, which was enriched in the KEGG pathway analysis. Stearoyl-CoA desaturase catalyzes the conversion of saturated fatty acids to unsaturated fatty acids, and it has been shown to protect rodent and human beta-cells from palmitate-induced ER stress and apoptosis [67,68]. Our result is in accordance with these previous studies and could provide an explanation for the absence of an increase in apoptosis in the palmitate-treated human islets.

Furthermore, the “one carbon pool by folate” pathway was enriched in the KEGG pathway analysis using both mRNA expression data and DNA methylation data. Altered expression of genes in this pathway may affect the amount of methyl donors, for example, S-adenosyl methionine in the islets exposed to palmitate and, thereby, contribute to differential DNA methylation. *SHMT2* and *MTHFD2* were both up-regulated due to palmitate exposure. The enzymes encoded by these genes are involved in the folate cycle which is linked to the methionine cycle, which in turn controls the amount of S-adenosyl methionine [69].

Importantly, our study demonstrates that palmitate directly affects the expression of genes that also show differential expression in islets from diabetic donors [70]. Additionally, some of our *in vitro* findings were validated in a cohort of islets from donors with a large spread in BMI (17.6 to 40.1 kg/m^2^) suggesting that lipid-induced changes seen *in vitro* correspond to those *in vivo*. While some previous studies have examined the impact of lipotoxicity on the expression of a limited number of candidate genes in human islets *in vitro*[24-26], the present study is to our knowledge the first to perform a genome-wide analysis of gene expression in lipotoxic-treated human islets of more than five donors [28,29].

It is debated whether lipotoxicity can occur in the absence of high glucose levels, a phenomenon known as glucolipotoxicity. However, previous *in vivo* studies in humans have shown that prolonged exposure (24 to 48 h) to free fatty acids, in the absence of elevated glucose levels, perturbs islet function [20]. Moreover, a recent study showed that the lipotoxic effect of palmitate occurs even at low concentrations of glucose in intact human islets [30]. Our findings provide further evidence that palmitate-induced lipotoxicity under normal glucose conditions results in extensive transcriptional changes and impaired insulin secretion in human islets. It is important, however, to note that our study only examined the effects of palmitate on human islets, and it is known that different fatty acids can have divergent, and even opposite, effects on cell function. Also, the *in vivo* fatty acid composition in plasma contains several different fatty acids [71] where palmitate is one of the most abundant saturated fatty acids. We can therefore in our study not rule out that other types of fatty acids have additional effects on human islets. However, our study provides evidence for palmitate-induced changes on gene expression, DNA methylation and insulin secretion which might be of relevance to phenotypes seen in obese individuals and T2D patients. Finally, as our previous studies have shown that the genome-wide methods used in the present study are robust and reproducible, we did not technically validate the array results in the present study [8,72-74].

## Conclusion

In conclusion, we have identified novel genes and metabolic pathways that are affected by palmitate exposure in human pancreatic islets. Importantly, a number of these genes also show differential gene expression in islets from patients with T2D. We have also shown for the first time that there are both global and specific changes in the DNA methylation pattern in the palmitate-treated islets which may affect mRNA expression. Together, these changes may contribute to the impaired insulin secretion seen in palmitate-treated human islets.

## Abbreviations

BSA: Bovine serum albumin; CpG: Cytosine-phosphate-guanine; FDR: False discovery rate; GWAS: Genome-wide association studies; HBSS: HEPES-balanced salt solution; KEGG: Kyoto Encyclopedia of Genes and Genomes; SNP: Single nucleotide polymorphism; T2D: Type 2 diabetes; TSS: Transcription start site; UTR: Untranslated region.

## Competing interests

The authors declare that there is no duality of interest associated with this manuscript.

## Authors’ contributions

EH designed and conducted the study, performed lab work, collected, analyzed and interpreted data, and wrote the manuscript. PV analyzed and interpreted data and reviewed and edited the manuscript. TD interpreted data and reviewed and edited the manuscript. KB performed lab work and reviewed and edited the manuscript. TR interpreted data and reviewed and edited the manuscript. MDN designed and conducted the study, collected and interpreted data, and reviewed and edited the manuscript. CL designed and conducted the study, interpreted data, and reviewed and edited the manuscript. EH, MDN and CL are guarantors of this work and, as such, had full access to all of the data in the study and take responsibility for the integrity of the data. All authors read and approved the final version of the manuscript.

## Supplementary Material

Additional file 1: Table S1 Characteristics of the human pancreatic donors included in the DNA methylation array analysis. Click here for file

Additional file 2: Table S2 Differentially expressed genes in human islets exposed to palmitate compared with the control (*q* <0.05). Click here for file

Additional file 3: Table S3 Biological pathways including all genes that exhibit differential mRNA expression (*q* <0.05) in human pancreatic islets exposed to palmitate compared to control treatment. Click here for file

Additional file 4: Table S4 Biological pathways including genes that exhibit decreased mRNA expression (*q* <0.05) in human pancreatic islets exposed to palmitate compared to control treatmet. Click here for file

Additional file 5: Table S5 Biological pathways including genes that exhibit increased mRNA expression (*q* <0.05) in human pancreatic islets exposed to palmitate compared to control treatmet. Click here for file

Additional file 6: Table S6 Differential mRNA expression (*q* <0.05) of candidate genes for type 2 diabetes (T2D) in human pancreatic islets exposed to palmitate versus control. DNA methylation data are displayed if the absolute difference in DNA methylation ≥3% and *P* <0.05. Click here for file

Additional file 7: Table S7 Differential mRNA expression (*q* <0.05) of candidate genes for type 2 diabetes (T2D) related traits in human pancreatic islets exposed to palmitate versus control. DNA methylation data are displayed if the absolute difference in DNA methylation ≥3% and *P* <0.05. Click here for file

Additional file 8: Table S8 Differential mRNA expression (*q* <0.05) of candidate genes for obesity in human pancreatic islets exposed to palmitate versus control. DNA methylation data are displayed if the absolute difference in DNA methylation ≥3% and *P* <0.05. Click here for file

Additional file 9: Table S9 Global DNA methylation in human pancreatic islets exposed to palmitate compared to control treatment. Average DNA methylation (%) in gene- and CpG-regions in human pancreatic islets treated for 48 h with control media or palmitate-containing media. Click here for file

Additional file 10: Table S10 CpG sites with differential DNA methylation in human pancreatic islets exposed to palmitate compared to control treatment *P* <0.05. Click here for file

Additional file 11: Table S11 Differential mRNA expression (*q* <0.05) of genes with a corresponding change in DNA methylation (*P* <0.05 and absolute DNA methylation difference ≥3%) of the nearest gene, in human pancreatic islets exposed to palmitate versus control. Click here for file

Additional file 12: Figure S1 Results from KEGG pathway analysis using genes that exhibit differential DNA methylation (*P* <0.05) in human pancreatic islets exposed to palmitate compared to control treatment. It includes only pathways which also displayed enrichment for genes with differential expression after palmitate treatment in human pancreatic islets. Click here for file

Additional file 13: Table S12 Biological pathways including genes that exhibit differential DNA methylation (*P* <0.05) in human pancreatic islets exposed to palmitate compared to control teatment. It includes only pathways which also displayed enrichment for genes with differential expression after palmitate treatment in human pancreatic islets. Click here for file

Additional file 14: Table S13 Infinium HumanMethylation450K BeadChip array probes with possible cross reactivity to other locations in the genome. Click here for file

Additional file 15: Table S14 Genes displaying significant difference in mRNA expression between control and palmitate-treated human pancreatic islets (*q* <0.05) as well as significant correlation between BMI and mRNA expression in human pancreatic islets (*P* <0.05). Click here for file

Additional file 16: Table S15 Differentially expressed genes (*P* <0.05) in islets from T2D versus non-diabetic donors overlapping with the 1,860 genes with altered expression in the palmitate-exposed islets. Click here for file
