# Supplementary material for: Effects of palmitate on genome-wide mRNA expression and DNA methylation patterns in human pancreatic islets
Source: BMC Med. 2014 Jun 23;12:103. doi: 10.1186/1741-7015-12-103 (PMC4065864; doi:10.1186/1741-7015-12-103)
Supplement: Additional file 1: Table S1 — Characteristics of the human pancreatic donors included in the DNA methylation array analysis. [file 1741-7015-12-103-S1.pdf]

Supplementary Table 1: Characteristics of the human pancreatic donors included in the DNA methylation array analysis.

n (male/female)	13 (8/5)
Age (years)	49 ± 16
BMI (kg/m^2)	26.4 ± 4.5
Hba1c (%)*	5.4 ± 0.7

Data are expressed as mean ± SD
**Data available for 10 donors (7 males/3 females)*
