# Supplementary material for: Effects of palmitate on genome-wide mRNA expression and DNA methylation patterns in human pancreatic islets
Source: BMC Med. 2014 Jun 23;12:103. doi: 10.1186/1741-7015-12-103 (PMC4065864; doi:10.1186/1741-7015-12-103)
Supplement: Additional file 3: Table S3 — Biological pathways including all genes that exhibit differential mRNA expression (q <0.05) in human pancreatic islets exposed to palmitate compared to control treatment. [file 1741-7015-12-103-S3.pdf]

**Supplementary Table 3:** Biological pathways including all genes that exhibit differential mRNA expression ($q$ <0.05) in human pancreatic islets due to palmitate treatment.

| **Pathway (total number of genes in the pathway)** | **Observed number of genes** | **Expected number of genes** | **Ratio Observed/ expected** | **Raw *P*-value** | **Adjusted *P*-value** |
|---|---|---|---|---|---|
| Ribosome (81) | 35 | 7.59 | 4.61 | $1.03 \times 10^{-15}$ | $1.85 \times 10^{-13}$ |
| Metabolic pathways (1,026) | 150 | 96.10 | 1.56 | $1.70 \times 10^{-08}$ | $1.53 \times 10^{-06}$ |
| Complement and coagulation cascades (67) | 21 | 6.28 | 3.35 | $4.24 \times 10^{-07}$ | $1.53 \times 10^{-05}$ |
| Glycolysis / Gluconeogenesis (60) | 20 | 5.62 | 3.56 | $2.59 \times 10^{-07}$ | $1.53 \times 10^{-05}$ |
| Pathways in cancer (323) | 56 | 30.25 | 1.85 | $4.54 \times 10^{-06}$ | 0.0001 |
| Focal adhesion (196) | 39 | 18.36 | 2.12 | $4.75 \times 10^{-06}$ | 0.0001 |
| Lysosome (115) | 26 | 10.77 | 2.41 | $1.77 \times 10^{-05}$ | 0.0004 |
| Arginine and proline metabolism (51) | 15 | 4.78 | 3.14 | $4.35 \times 10^{-05}$ | 0.0007 |
| ECM-receptor interaction (82) | 20 | 7.68 | 2.60 | $5.13 \times 10^{-05}$ | 0.0008 |
| One carbon pool by folate (15) | 7 | 1.40 | 4.98 | 0.0002 | 0.0024 |
| Regulation of actin cytoskeleton (204) | 35 | 19.11 | 1.83 | 0.0003 | 0.0032 |
| Cell cycle (121) | 24 | 11.33 | 2.12 | 0.0003 | 0.0032 |
| p53 signaling pathway (67) | 16 | 6.28 | 2.55 | 0.0004 | 0.0040 |
| Methane metabolism (6) | 4 | 0.56 | 7.12 | 0.0010 | 0.0078 |
| Glutathione metabolism (47) | 12 | 4.40 | 2.73 | 0.0010 | 0.0078 |
| Retinol metabolism (47) | 12 | 4.40 | 2.73 | 0.0010 | 0.0078 |
| Pancreatic cancer (71) | 15 | 6.65 | 2.26 | 0.0021 | 0.0151 |
| Tryptophan metabolism (38) | 10 | 3.56 | 2.81 | 0.0021 | 0.0151 |
| Fructose and mannose metabolism (33) | 9 | 3.09 | 2.91 | 0.0026 | 0.0180 |
| Insulin signaling pathway (134) | 23 | 12.55 | 1.83 | 0.0032 | 0.0198 |
| Adherens junction (74) | 15 | 6.93 | 2.16 | 0.0032 | 0.0198 |
| Biosynthesis of unsaturated fatty acids (22) | 7 | 2.06 | 3.40 | 0.0030 | 0.0198 |
| Fatty acid metabolism (41) | 10 | 3.84 | 2.60 | 0.0038 | 0.0221 |
| Antigen processing and presentation (55) | 12 | 5.15 | 2.33 | 0.0043 | 0.0242 |
| O-Glycan biosynthesis (30) | 8 | 2.81 | 2.85 | 0.0052 | 0.0253 |
| ErbB signaling pathway (85) | 16 | 7.96 | 2.01 | 0.0051 | 0.0253 |
| Gap junction (85) | 16 | 7.96 | 2.01 | 0.0051 | 0.0253 |
| Maturity onset diabetes of the young (24) | 7 | 2.25 | 3.11 | 0.0052 | 0.0253 |
| Ascorbate and aldarate metabolism (13) | 5 | 1.22 | 4.11 | 0.0049 | 0.0253 |
| Propanoate metabolism (32) | 8 | 3.00 | 2.67 | 0.0079 | 0.0374 |

| | | | | | |
|---|---|---|---|---|---|
| Neurotrophin signaling pathway (122) | 20 | 11.43 | 1.75 | 0.0094 | 0.0411 |
| Arachidonic acid metabolism (54) | 11 | 5.06 | 2.17 | 0.0104 | 0.0435 |

| **Pathway** | **Observed genes** |
|---|---|
| Ribosome | *RPS20, RPS8, RPS2, RPS5, RPL30, RPS17, RPS17, RPL11, RPL18, RPL10, RPL10, RPL17, RPL17, RPS11, RPL27, RPS18, RPS18, RPS18, RPS23, RPL29, RPL35, RPL36AL, RPS25, RPL41, RPS10, RPL38, RPL8, RPS27, RPL18A, RPL10A, RPL27A, RPS9, RPLP0, RPL35A, RPL12, RPL12, RPS15A, RPS7, RPS19, RPL24, FAU* |
| Metabolic pathways | *ARG2, ACSL1, UQCRB, MCCC1, ASS1, NANS, CDIPT, GLUL, CHPF, ACSS2, PNLIPRP2, PAPSS1, ALDH1A3, DPYD, DLD, NDUFS2, DNMT1, UGT2B10, PDHA1, LAP3, GALNT5, UPP1, HSD17B2, RRM2, GALNT7, ODC1, IDH1, PLA2G10, CYP4F3, PRIM1, PYCR1, MAN2A2, P4HA2, FBP1, NNT, CYP3A5, BDH2, TYMS, PIK3C2B, ATP6V0E1, PFKP, CYP2J2, DHCR24, ASAH1, , DHRS3, GALNT1, CTPS, PCK1, ST6GALNAC1, AGL, ALDH3A2, PIGS, FDFT1, ADH1A, HSD17B8, HSD17B8, HSD17B8, ALG6, RDH10, AMD1, TBXAS1, FASN, SCP2, HGSNAT, ADH1C, CAT, IDS, COX17, COX17, MTHFD2, HGD, HGD, PAH, CYP4F2, PPAP2B, GCH1, P4HA3, SGMS2, UGT2A3, PLD1, HEXA, ACSS1, PTDSS1, PHPT1, ACSM3, HIBADH, GALNT12, NDUFB5, ATP5I, SDHA, PPAP2A, ATP6V0B, ADI1, ASNS, KMO, CMPK1, AMACR, AHCYL1, MMAB, GATM, PLA2G7, POLR2G, MVD, ACO1, ANPEP, PON2, TCIRG1, DDOST, MAOA, GMPPB, HSD3B1, B4GALT3, ST3GAL1, LTA4H, ALDOB, NDUFS1, PGM2, ENO2, DGKZ, SORD, RPE, PMM2, ST6GALNAC4, ADH5, MAT2B, POLR3D, GART, FUT2, HSD11B1, PNLIP, CHST4, SHMT2, ALDH7A1, ADH6, PCCB, CPS1, SHMT1, B3GNT2, ALDH2, GUK1, AKR1B10, GCNT3, POLD3, CYP3A4, CYP2C18, B3GALT5, ACSS3, ACOX1, DHFR, HK1, ACADM, AFMID, PIP5K1B* |
| Complement and coagulation cascades | *C3, CFH, KLKB1, C6, TFPI, CFI, PLAT, F11, C1QB, SERPINA5, CD59, F10, SERPING1, PLAU, C1QC, CFB, CFB, CFB, F2R, C4BPA, BDKRB2, THBD, C3AR1* |
| Glycolysis / Gluconeogenesis | *PDHA1, ADH1A, ADH6, ALDH2, PGM2, ENO2, GALM, ADH1C, ADH5, ACSS2, FBP1, ALDH1A3, PCK1, ALDH7A1, HK1, ALDOB, ACSS1, DLD, ALDH3A2, PFKP* |
| Pathways in cancer | *HSP90B1, FN1, FGF13, MTOR, EGF, RASSF1, ERBB2, TGFBR1, STAT5B, CDKN1A, PDGFRA, TGFBR2, FGF19, CCNE1, WNT5A, LAMB3, PIAS1, LAMA3, IGF1R, IGF1R, CDH1, CTNNB1, MITF, COL4A1, NFKB2, PDGFRB, LAMA4, LAMC1, ACVR1B, CDK4, AKT2, PIK3R1, SKP2, KRAS, LAMA2, FOS, TCF7L2, SMAD3, JAK1, FGFR2, NCOA4, HIF1A, CCDC6, IL8, LAMB1, STAT1, HDAC1, KITLG, HGF, ITGAV, DVL1, PLD1, MAPK1, HRAS, ARNT, CDKN2A, HDAC2* |
| Focal adhesion | *SHC4, FN1, DOCK1, KDR, EGF, ERBB2, PDGFRA, PPP1CC, COL6A3, LAMB3, SPP1, LAMA3, SHC1, CTNNB1, IGF1R, IGF1R, ITGB3, COL4A1, TNN, ITGB6, CAV2, PDGFRB, LAMA4, COL5A2, LAMC1, ITGA4, AKT2, COL1A1, PIK3R1, ITGA5, LAMA2, PAK3, LAMB1,* |

| | |
|---|---|
| | *PDGFD, ACTN4, HGF, COL1A2, ITGAV, MAPK1, HRAS* |
| Lysosome | *LAMP1, CTSE, ASAH1, GGA2, HGSNAT, LIPA, AP3S1, NEU1, NEU1, NEU1, IDS, CD68, ATP6V0B, AP1M2, ENTPD4, CTSS, TCIRG1, CTSC, CTSO, GNPTAB, LAPTM5, LAPTM4B, CTSA, CTSD, HEXA, CTSH, ACP5, LAMP3* |
| Arginine and proline metabolism | *ARG2, ALDH2, CPS1, LAP3, P4HA2, P4HA3, AMD1, MAOA, ASS1, ALDH7A1, ODC1, GATM, GLUL, ALDH3A2, PYCR1* |
| ECM-receptor interaction | *LAMA4, COL5A2, FN1, LAMC1, COL1A1, ITGA4, ITGA5, LAMA2, SDC1, COL6A3, LAMB3, SPP1, LAMA3, LAMB1, ITGB3, COL4A1, ITGAV, COL1A2, TNN, ITGB6* |
| One carbon pool by folate | *DHFR, TYMS, SHMT2, GART, SHMT1, MTHFD2, MTFMT* |
| Regulation of actin cytoskeleton | *FN1, FGF13, DOCK1, EGF, ENAH, NCKAP1, DIAPH3, PDGFRA, FGF19, PPP1CC, IQGAP2, ITGB3, F2R, ITGB6, PDGFRB, CFL1, LIMK2, CYFIP1, ITGA4, PIK3R1, EZR, ITGA5, KRAS, ARHGEF12, ITGB2, PAK3, CSK, FGFR2, PDGFD, ACTN4, ITGAV, MAPK1, HRAS, BDKRB2, PIP5K1B* |
| Cell cycle | *CDKN1C, PRKDC, CDK4, SKP2, CHEK1, CDKN1A, CCNB1, MCM7, CDC26, CCNE1, CDC2, SMAD3, WEE1, CCNA2, PCNA, TTK, HDAC1, ATR, TFDP2, CCNB2, YWHAB, CDKN2A, HDAC2, SFN* |
| p53 signaling pathway | *SESN2, SESN3, PMAIP1, CDK4, TNFRSF10B, RRM2, CHEK1, ATR, CDKN1A, CCNB1, CCNB2, TP53I3, CCNE1, CDC2, CDKN2A, SFN* |
| Methane metabolism | *CAT, ADH5, SHMT2, SHMT1* |
| Glutathione metabolism | *GSTK1, GPX3, GSTM2, LAP3, GPX2, RRM2, IDH1, ODC1, MGST1, ANPEP, GSTA1, GPX4* |
| Retinol metabolism | *ADH5, ADH1A, ADH6, CYP1A1, RDH10, CYP3A5, UGT2A3, DHRS3, CYP3A4, CYP2C18, UGT2B10, ADH1C* |
| Pancreatic cancer | *SMAD3, JAK1, ACVR1B, AKT2, CDK4, EGF, PIK3R1, STAT1, ERBB2, TGFBR1, KRAS, PLD1, MAPK1, TGFBR2, CDKN2A* |
| Tryptophan metabolism | *CAT, ALDH2, CYP1A1, KMO, MAOA, ALDH7A1, AFMID, ALDH3A2, WARS, CYP1B1* |
| Fructose and mannose metabolism | *SORD, FBP1, AKR1B10, GMPPB, HK1, PMM2, ALDOB, PHPT1, PFKP* |
| Insulin signaling pathway | *SHC4, MTOR, AKT2, PRKAA1, PIK3R1, FASN, KRAS, PRKAR2B, PPP1CC, IRS4, PRKAG3, IRS2, INSR, SOCS1, SHC1, FBP1, PCK1, MKNK2, HK1, PPARGC1A, MAPK1, HRAS, PRKAR1A* |
| Adherens junction | *SMAD3, INSR, IGF1R, IGF1R, CTNNB1, CDH1, ACVR1B, ACTN4, ERBB2, TGFBR1, CTNND1, MAPK1, TGFBR2, TCF7L2, SNAI2, FARP2* |
| Biosynthesis of unsaturated fatty acids | *FADS1, SCD, SCD, FADS2, PECR, PTPLB, SCD5, ACOX1* |
| Fatty acid metabolism | *ADH5, ADH1A, ADH6, ACSL1, ALDH2, ACOX1, ALDH7A1, ACADM, ALDH3A2, ADH1C* |

| | |
|---|---|
| Antigen processing and presentation | *HLA-DRA, HLA-DRA, HLA-DMA, HLA-DMA, HLA-DMA, CTSS, HSPA5, HLA-DMB, HLA-DMB, IFI30, B2M, RFX5, CREB1, HLA-DPB1, CD74, HSPA6* |
| O-Glycan biosynthesis | *GALNT1, B4GALT5, GALNT5, GALNT12, GCNT3, ST6GALNAC1, GALNT7, ST3GAL1* |
| ErbB signaling pathway | *SHC4, MTOR, AKT2, EGF, PIK3R1, ERBB2, STAT5B, KRAS, CDKN1A, PAK3, SHC1, NCK2, ERBB3, MAPK1, GAB1, HRAS* |
| Gap junction | *EGF, TUBA1C, KRAS, TUBB6, ITPR2, PDGFRA, CDC2, ADCY2, TUBA8, TUBB2B, PDGFD, GNAQ, MAPK1, HRAS, PDGFRB, TUBA4A* |
| Maturity onset diabetes of the young | *HHEX, FOXA2, NKX2-2, HNF4G, NR5A2, HNF1B, NEUROD1* |
| Ascorbate and aldarate metabolism | *ALDH7A1, UGT2A3, ALDH2, ALDH3A2, UGT2B10* |
| Propanoate metabolism | *PCCB, ALDH2, ACSS3, ACSS2, ALDH7A1, ACADM, ACSS1, ALDH3A2* |
| Neurotrophin signalling pathway | *SHC4, RPS6KA1, AKT2, PIK3R1, KRAS, PSEN1, IRS4, IRS2, IRAK1, KIDINS220, SHC1, CSK, PRKCD, ARHGDIA, NFKBIB, MAPK1, HRAS, GAB1, YWHAB, MAP3K1* |
| Arachidonic acid metabolism | *GPX3, CYP4F2, CYP2J2, GPX2, TBXAS1, AKR1C3, LTA4H, CYP4F3, PLA2G10, CYP2C18, GPX4* |
