# Supplementary material for: Effects of palmitate on genome-wide mRNA expression and DNA methylation patterns in human pancreatic islets
Source: BMC Med. 2014 Jun 23;12:103. doi: 10.1186/1741-7015-12-103 (PMC4065864; doi:10.1186/1741-7015-12-103)
Supplement: Additional file 4: Table S4 — Biological pathways including genes that exhibit decreased mRNA expression (q <0.05) in human pancreatic islets exposed to palmitate compared to control treatmet. [file 1741-7015-12-103-S4.pdf]

Supplementary Table 4: Biological pathways including genes that exhibit differential down-regulated mRNA expression (q <0.05) after palmitate treatment in human pancreatic islets.

Pathway (total number of genes in the pathway)	Observed number of genes	Expected number of genes	Ratio Observed/ expected	Raw *P*-value	Adjusted *P*-value
Metabolic pathways (1,026)	113	63.85	1.77	1.57×10^{-09}	1.22×10^{-07}
Complement and coagulation cascades (67)	20	4.17	4.80	2.26×10^{-09}	1.22×10^{-07}
Glycolysis / Gluconeogenesis (60)	17	3.73	4.55	8.47×10^{-08}	3.43×10^{-06}
Focal adhesion (196)	33	12.20	2.71	1.58×10^{-07}	4.27×10^{-06}
Regulation of actin cytoskeleton (204)	32	12.70	2.52	1.23×10^{-06}	2.85×10^{-05}
Pathways in cancer (323)	43	20.10	2.14	2.01×10^{-06}	4.03×10^{-05}
ECM-receptor interaction (82)	18	5.10	3.53	2.24×10^{-06}	4.03×10^{-05}
Retinol metabolism (47)	12	2.93	4.10	2.18×10^{-05}	0.0004
Adherens junction (74)	15	4.61	3.26	4.21×10^{-05}	0.0006
Lysosome (115)	19	7.16	2.65	8.47×10^{-05}	0.0011
Arginine and proline metabolism (51)	11	3.17	3.47	0.0002	0.0022
Pancreatic cancer (71)	13	4.42	2.94	0.0004	0.0038
O-Glycan biosynthesis (30)	8	1.87	4.28	0.0004	0.0038
Propanoate metabolism (32)	8	1.99	4.02	0.0006	0.0054
Fatty acid metabolism (41)	9	2.55	3.53	0.0008	0.0065
Ascorbate and aldarate metabolism (13)	5	0.81	6.18	0.0008	0.0065
Cell cycle (121)	17	7.53	2.26	0.0013	0.0096
One carbon pool by folate (15)	5	0.93	5.36	0.0016	0.0112
Antigen processing and presentation (55)	10	3.42	2.92	0.0019	0.0114
Tryptophan metabolism (38)	8	2.36	3.38	0.0020	0.0116
Glutathione metabolism (47)	9	2.93	3.08	0.0022	0.0123
Butanoate metabolism (32)	7	1.99	3.51	0.0030	0.0157
Maturity onset diabetes of the young (24)	6	1.49	4.02	0.0029	0.0157
Methane metabolism (6)	3	0.37	8.03	0.0042	0.0206
Valine, leucine and isoleucine degradation (43)	8	2.68	2.99	0.0045	0.0214
Axon guidance (127)	16	7.90	2.02	0.0055	0.0241
Linoleic acid metabolism (27)	6	1.68	3.57	0.0055	0.0241
Arachidonic acid metabolism (54)	9	3.36	2.68	0.0057	0.0243
Notch signaling pathway (46)	8	2.86	2.79	0.0069	0.0287
Histidine metabolism (29)	6	1.80	3.32	0.0079	0.0320

beta-Alanine metabolism (22)	5	1.37	3.65	0.0100	0.0395
Pyruvate metabolism (40)	7	2.49	2.81	0.0109	0.0420

**Pathway**	**Observed genes**
Metabolic pathways	*UQCRB, GALNT12, MCCC1, NDUFB5, ASS1, CDIPT, GLUL, SDHA, PPAP2A, ADI1, ACSS2, PNLIPRP2, PAPSS1, ALDH1A3, CMPK1, DPYD, AMACR, AHCYL1, DLD, NDUFS2, GATM, DNMT1, UGT2B10, PDHA1, PLA2G7, LAP3, GALNT5, ACO1, HSD17B2, RRM2, GALNT7, IDH1, PLA2G10, CYP4F3, ANPEP, PRIM1, MAN2A2, PON2, TCIRG1, FBP1, NNT, CYP3A5, BDH2, MAOA, TYMS, PIK3C2B, ST3GAL1, LTA4H, ALDOB, ATP6V0E1, NDUFS1, PGM2, SORD, CYP2J2, DHCR24, ASAH1, NDUFA4, RPE, DHRS3, ADH5, MAT2B, GALNT1, GART, FUT2, HSD11B1, PNLIP, CTPS, CHST4, ALDH7A1, ST6GALNAC1, AGL, ALDH3A2, PIGS, FDFT1, ADH1A, ADH6, PCCB, CPS1, B3GNT2, ALDH2, SHMT1, ALG6, AMD1, RDH10, AKR1B10, TBXAS1, SCP2, GCNT3, HGSNAT, POLD3, CYP2C18, CYP3A4, B3GALT5, ADH1C, CAT, ACSS3, HGD, HGD, CYP4F2, PAH, PPAP2B, ACOX1, P4HA3, DHFR, UGT2A3, ACADM, PLD1, HK1, ACSS1, AFMID, PTDSS1, HIBADH, ACSM3, PIP5K1B*
Complement and coagulation cascades	*C3, CFH, KLKB1, C6, TFPI, CFI, PLAT, F11, C1QB, SERPINA5, CD59, PLAU, SERPING1, C1QC, CFB, CFB, CFB, F2R, C4BPA, BDKRB2, THBD, C3AR1*
Glycolysis / Gluconeogenesis	*ADH5, ADH1A, PDHA1, ADH6, ALDH2, PGM2, ACSS2, FBP1, ALDH1A3, ALDH7A1, GALM, HK1, DLD, ACSS1, ALDOB, ALDH3A2, ADH1C*
Focal adhesion	*FN1, DOCK1, KDR, EGF, ERBB2, PDGFRA, PPP1CC, COL6A3, SPP1, LAMA3, IGF1R, IGF1R, CTNNB1, ITGB3, COL4A1, ITGB6, CAV2, PDGFRB, LAMA4, LAMC1, COL5A2, AKT2, COL1A1, ITGA4, PIK3R1, ITGA5, LAMA2, LAMB1, PDGFD, ACTN4, HGF, ITGAV, COL1A2, MAPK1*
Regulation of actin cytoskeleton	*FN1, DOCK1, EGF, ENAH, NCKAP1, DIAPH3, PDGFRA, PPP1CC, FGF19, IQGAP2, ITGB3, ITGB6, F2R, LIMK2, CFL1, PDGFRB, CYFIP1, ITGA4, PIK3R1, EZR, ITGA5, KRAS, ARHGEF12, ITGB2, FGFR2, CSK, ACTN4, PDGFD, ITGAV, MAPK1, BDKRB2, PIP5K1B*
Pathways in cancer	*FN1, MTOR, EGF, ERBB2, TGFBR1, PDGFRA, TGFBR2, FGF19, CCNE1, WNT5A, PIAS1, LAMA3, IGF1R, IGF1R, CTNNB1, CDH1, MITF, COL4A1, PDGFRB, LAMA4, LAMC1, AKT2, ACVR1B, PIK3R1, SKP2, KRAS, LAMA2, TCF7L2, SMAD3, JAK1, HIF1A, CCDC6, NCOA4, FGFR2, LAMB1, STAT1, HDAC1, KITLG, HGF, PLD1, ITGAV, MAPK1, ARNT, HDAC2*
ECM-receptor interaction	*LAMA4, COL5A2, LAMC1, FN1, ITGA4, COL1A1, ITGA5, LAMA2, SDC1, COL6A3, SPP1, LAMA3, LAMB1, ITGB3, COL4A1, ITGAV, COL1A2, ITGB6*
Retinol metabolism	*ADH5, ADH1A, ADH6, CYP1A1, RDH10, CYP3A5, UGT2A3, DHRS3, CYP2C18, CYP3A4, UGT2B10, ADH1C*

Adherens junction	*SMAD3, INSR, IGF1R, IGF1R, CTNNB1, CDH1, ACVR1B, ERBB2, ACTN4, TGFBR1, CTNND1, MAPK1, TGFBR2, SNAI2, TCF7L2, FARP2*
Lysosome	*LAMP1, CTSE, ASAH1, HGSNAT, AP3S1, LIPA, CTSS, AP1M2, ENTPD4, TCIRG1, CTSC, CTSO, GNPTAB, LAPTM5, LAPTM4B, CTSA, CTSD, CTSH, ACP5*
Arginine and proline metabolism	*CPS1, ALDH2, LAP3, P4HA3, AMD1, MAOA, ASS1, ALDH7A1, GATM, GLUL, ALDH3A2*
Pancreatic cancer	*SMAD3, JAK1, ACVR1B, AKT2, PIK3R1, EGF, STAT1, ERBB2, TGFBR1, KRAS, PLD1, MAPK1, TGFBR2*
O-Glycan biosynthesis	*PCCB, ACSS3, ALDH2, ACSS2, ALDH7A1, ACADM, ACSS1, ALDH3A2*
Propanoate metabolism	*ADH5, ADH1A, ADH6, ALDH2, ACOX1, ALDH7A1, ACADM, ALDH3A2, ADH1C*
Fatty acid metabolism	*ADH5, ADH1A, ADH6, ALDH2, ACOX1, ALDH7A1, ACADM, ALDH3A2, ADH1C*
Ascorbate and aldarate metabolism	*ALDH7A1, UGT2A3, ALDH2, ALDH3A2, UGT2B10*
Cell cycle	*PRKDC, SKP2, CHEK1, CCNB1, MCM7, CDC2, CCNE1, SMAD3, CCNA2, PCNA, TTK, HDAC1, ATR, TFDP2, CCNB2, HDAC2, YWHAB*
One carbon pool by folate	*DHFR, TYMS, GART, SHMT1, MTFMT*
Antigen processing and presentation	*HLA-DRA, HLA-DRA, HLA-DMA, HLA-DMA, HLA-DMA, CTSS, HLA-DMB, HLA-DMB, IFI30, B2M, RFX5, CREB1, HLA-DPB1, CD74*
Tryptophan metabolism	*MAOA, CAT, ALDH7A1, ALDH2, CYP1A1, AFMID, ALDH3A2, CYP1B1*
Glutathione metabolism	*GSTK1, GSTM2, LAP3, GPX2, RRM2, IDH1, MGST1, GSTA1, ANPEP*
Butanoate metabolism	*PDHA1, ALDH7A1, ALDH2, ALDH3A2, AKR1B10, ACSM3, BDH2*
Maturity onset diabetes of the young	*HHEX, NKX2-2, NR5A2, HNF4G, HNF1B, NEUROD1*
Methane metabolism	*ADH5, CAT, SHMT1*
Valine, leucine and isoleucine degradation	*PCCB, ALDH2, MCCC1, ALDH7A1, ACADM, DLD, ALDH3A2, HIBADH*
Axon guidance	*EFNB2, ROBO1, ARHGEF12, KRAS, SEMA6D, PLXNB2, SEMA6A, CDK5, ABLIM1, NCK2, NTN4, NRP1, MAPK1, CFL1, LIMK2, CHP*
Linoleic acid metabolism	*CYP2J2, PLA2G10, CYP2C18, CYP3A4, AKR1B10, CYP3A5*
Arachidonic acid metabolism	*CYP4F2, CYP2J2, GPX2, TBXAS1, LTA4H, AKR1C3, PLA2G10, CYP4F3, CYP2C18*
Notch sigling pathway	*HDAC1, NOTCH3, JAG1, NOTCH2, LFNG, PSEN1, HDAC2, DTX4*
Histidine metabolism	*MAOA, ALDH7A1, ALDH2, HNMT, ALDH3A2, ALDH1A3*

beta-Alanine metabolism	*ALDH7A1*, *ACADM*, *ALDH2*, *DPYD*, *ALDH3A2*
Pyruvate metabolism	*PDHA1*, *ALDH7A1*, *ALDH2*, *ACSS2*, *ACSS1*, *DLD*, *ALDH3A2*
