# Supplementary material for: Effects of palmitate on genome-wide mRNA expression and DNA methylation patterns in human pancreatic islets
Source: BMC Med. 2014 Jun 23;12:103. doi: 10.1186/1741-7015-12-103 (PMC4065864; doi:10.1186/1741-7015-12-103)
Supplement: Additional file 5: Table S5 — Biological pathways including genes that exhibit increased mRNA expression (q <0.05) in human pancreatic islets exposed to palmitate compared to control treatmet. [file 1741-7015-12-103-S5.pdf]

Supplementary Table 5: Biological pathways including genes that exhibit differential up-regulated mRNA expression (q <0.05) after palmitate treatment in human pancreatic islets.

Pathway (total number of genes in the pathway)	Observed number of genes	Expected number of genes	Ratio Observed/ expected	Raw *P*-value	Adjusted *P*-value
Ribosome (81)	33	2.55	12.96	1.43×10^{-28}	1.34×10^{-26}
Aminoacyl-tRNA biosynthesis (40)	7	1.26	5.57	0.0002	0.0094
PPAR signaling pathway (67)	8	2.11	3.80	0.0012	0.0305
Amino sugar and nucleotide sugar metabolism (43)	6	1.35	4.44	0.0021	0.0329

Pathway	Observed genes
Ribosome	*RPS20, RPS8, RPS2, RPS5, RPL30, RPL11, RPS17, RPL18, RPL10, RPL17, RPS11, RPL27, RPS18, RPL29, RPL35, RPS25, RPL36AL, RPS10, RPL38, RPL8, RPL41, RPS27, RPL18A, RPL10A, RPS9, RPLP0, RPL35A, RPL12, RPS15A, RPS7, RPS19, RPL24, FAU*
Aminoacyl-tRNA biosynthesis	*CARS, MARS, GARS, IARS, YARS, AARS, WARS*
PPAR signaling pathway	*FABP3, FADS2, SCD, ACSL1, SLC27A2, ANGPTL4, UBC, PCK1*
Amino sugar and nucleotide sugar metabolism	*GMPPB, NANS, PMM2, HEXA, NAGK, PGM3*
