# Supplementary material for: Effects of palmitate on genome-wide mRNA expression and DNA methylation patterns in human pancreatic islets
Source: BMC Med. 2014 Jun 23;12:103. doi: 10.1186/1741-7015-12-103 (PMC4065864; doi:10.1186/1741-7015-12-103)
Supplement: Additional file 6: Table S6 — Differential mRNA expression (q <0.05) of candidate genes for type 2 diabetes (T2D) in human pancreatic islets exposed to palmitate versus control. DNA methylation data are displayed if the absolute difference in DNA methylation ≥3% and P <0.05. [file 1741-7015-12-103-S6.pdf]

Supplementary Table 6: Differential mRNA expression (*q* <0.05) of candidate genes for type 2 diabetes (T2D) in human pancreatic islets exposed to palmitate versus control. DNA methylation data is displayed if the absolute difference in DNA methylation ≥3% and *P* <0.05.

**T2D candidate genes**	**mRNA expression**							**DNA methylation (%)**					
	**Probe ID**	**Chr**	**Control mean ± sd**	**Palmitate mean ± sd**	**Diff. palmitate-control**	***P*-value**	***q*-value**	**Probe ID**	**Region**	**Control mean ± sd**	**Palmitate mean ± sd**	**Diff. palmitate-control**	***P*-value**
CDKN2A	8160441	9	81.2 ± 12.7	99.6 ± 16.4	18.34	0.0024	0.041						
GLIS3	8159900	9	441.4 ± 66.2	394.9 ± 47.8	-46.51	0.0012	0.027	cg14641122	Body; Open sea	33.4 ± 8.2	36.7 ± 9.3	3.2	0.010
								cg14269813	Body; Open sea	58.2 ± 11.1	61.6 ± 11.3	3.4	0.016
GRB14	8056327	2	97.5 ± 15.5	81.5 ± 24.1	-15.99	0.0024	0.041						
HHEX	7929282	10	427.6 ± 78.2	340.5 ± 77.3	-87.03	0.0002	0.013						
HNF1B	8014591	17	193.1 ± 53.2	154.7 ± 48.4	-38.38	0.0005	0.017						
ITGB6	8056184	2	790.1 ± 379.4	623.3 ± 250.2	-166.88	0.0012	0.027						
KIF11	7929258	10	27.4 ± 7.0	17.8 ± 4.3	-9.60	0.0005	0.017						
MAEA	8093462	4	541.4 ± 29.9	512.1 ± 32.2	-29.38	0.0012	0.027						
NOTCH2	7919095	1	381.1 ± 126.7	281.3 ± 105.9	-99.81	0.0002	0.013						
PTPRD	8160040	9	64.9 ± 17.7	57.1 ± 16.7	-7.85	0.0024	0.041						
SLC30A8	8148003	8	5,399.1 ± 1,886.6	4,762.5 ± 1,777.0	-636.61	0.0034	0.049						
SLC44A3	7903144	1	316.4 ± 76.9	260.8 ± 68.5	-55.59	0.0034	0.049						
TCF7L2	7930537	10	215.1 ± 30.9	180.2 ± 28.9	-34.89	0.0002	0.013	cg26775558	Body; Open sea	56.5 ± 10.0	60.4 ± 9.2	3.9	0.006
TP53INP1	8151890	8	426.4 ± 122.2	371.3 ± 94.1	-55.04	0.0024	0.041						
TSPAN8	7964927	12	1,740.4 ± 604.9	870.6 ± 550.3	-869.75	0.0002	0.013						
VPS26A	7927972	10	950.4 ± 120.1	858.1 ± 145.6	-92.31	0.0012	0.027						
