# Supplementary material for: Effects of palmitate on genome-wide mRNA expression and DNA methylation patterns in human pancreatic islets
Source: BMC Med. 2014 Jun 23;12:103. doi: 10.1186/1741-7015-12-103 (PMC4065864; doi:10.1186/1741-7015-12-103)
Supplement: Additional file 7: Table S7 — Differential mRNA expression (q <0.05) of candidate genes for type 2 diabetes (T2D) related traits in human pancreatic islets exposed to palmitate versus control. DNA methylation data are displayed if the absolute difference in DNA methylation ≥3% and P <0.05. [file 1741-7015-12-103-S7.pdf]

Supplementary Table 7: Differential mRNA expression (*q* <0.05) of candidate genes for type 2 diabetes (T2D) related traits in human pancreatic islets exposed to palmitate versus control. DNA methylation data is displayed if the absolute difference in DNA methylation ≥3% and *P* <0.05.

T2D trait candidate gene	Trait*	mRNA expression							DNA methylation (%)					
		**Probe ID**	**Chr**	**Control mean ± sd**	**Palmitate mean ± sd**	**Diff. palmitate -control**	***P*-value**	***q*-value**	**Probe ID**	**Region**	**Control mean ± sd**	**Palmitate mean ± sd**	**Diff. palmitate -control**	***P*-value**
ATP11A	1	7970162	13	184.9 ± 37.8	153.7 ± 35.7	31.2	0.0017	0.033	cg02738374	Body; Island	80.0 ± 3.7	83.4 ± 2.7	3.4	0.001
									cg19293163	Body; N Shore	63.9 ± 7.4	68.4 ± 5.6	4.5	0.005
FADS1	2, 3	7948612	11	396.9 ± 102.4	588.2 ± 187.9	-191.3	0.00024	0.013						
FOXA2	3	8065344	20	320.9 ± 47.1	353.7 ± 54.1	-32.8	0.0024	0.041						
GCKR	2, 3, 4, 5, 6	8040960	2	80.8 ± 32.2	104.2 ± 65.0	-23.4	0.0012	0.027	cg20229788	TSS1500; S Shore	24.3 ± 6.3	27.4 ± 5.9	3.1	0.013
GLIS3	2, 3	8159900	9	441.4 ± 66.2	394.9 ± 47.8	46.5	0.0012	0.027	cg14641122	Body; Open sea	33.4 ± 8.2	36.7 ± 9.3	3.2	0.010
									cg14269813	Body; Open sea	58.2 ± 11.1	61.6 ± 11.3	3.4	0.016
GRB14	5	8056327	2	97.5 ± 15.5	81.5 ± 24.1	16.0	0.0024	0.041						
HK1	1	7928019	10	169.7 ± 43.7	150.4 ± 43.5	19.3	0.0024	0.041	cg04031454	TSS1500; Open sea	65.9 ± 4.4	69.1 ± 4.2	3.2	0.004
PCSK1	3	8113234	5	4,238.9 ± 1,705.4	5,381.2 ± 1,765.8	-1,142.3	0.0002	0.013						
PROX1	2, 3	7909681	1	153.2 ± 26.4	131.9 ± 26.7	21.3	0.00024	0.013						
SEZ6L	7	8072088	22	344.9 ± 136.9	407.1 ± 174.6	-62.2	0.0017	0.033						
SLC30A8	2, 3, 1, 8	8148003	8	5,399.1 ± 1,886.6	4,762.5 ± 1,777.0	636.6	0.0034	0.049						
TCF7L2	2, 3, 5, 1, 8, 9	7930537	10	215.1 ± 30.9	180.2 ± 28.9	34.9	0.00024	0.013	cg26775558	Body; Open sea	56.5 ± 10.0	60.4 ± 9.2	3.9	0.006
TMPRSS6	1	8075865	22	52.4 ± 5.6	59.0 ± 9.0	-6.6	0.00073	0.020						

Note: *1) Glycated hemoglobin levels, 2) Fasting glucose-related traits, 3) Fasting glucose-related traits (interaction with BMI), 4) Fasting insulin-related traits, 5) Fasting insulin-related traits (interaction with BMI), 6) Fasting plasma glucose, 7) Insulin resistance/response, 8) Type 2 diabetes and other traits, 9)Two-hour glucose challenge*
