# Supplementary material for: Effects of palmitate on genome-wide mRNA expression and DNA methylation patterns in human pancreatic islets
Source: BMC Med. 2014 Jun 23;12:103. doi: 10.1186/1741-7015-12-103 (PMC4065864; doi:10.1186/1741-7015-12-103)
Supplement: Additional file 8: Table S8 — Differential mRNA expression (q <0.05) of candidate genes for obesity in human pancreatic islets exposed to palmitate versus control. DNA methylation data are displayed if the absolute difference in DNA methylation ≥3% and P <0.05. [file 1741-7015-12-103-S8.pdf]

Supplementary Table 8: Differential mRNA expression (q <0.05) of candidate genes for obesity in human pancreatic islets exposed to palmitate versus control. DNA methylation data is displayed if the absolute difference in DNA methylation ≥3% and P <0.05.

**Obesity candidate gene**	**mRNA expression**							**DNA methylation (%)**					
	**Probe ID**	**Chr**	**Control mean ± sd**	**Palmitate mean ± sd**	**Diff. palmitate-control**	***P*-value**	***q*-value**	**Probe ID**	**Region**	**Control mean ± sd**	**Palmitate mean ± sd**	**Diff. palmitate-control**	***P*-value**
ASAH1	8149534	8	2,772.5 ± 351.5	2,517.6 ± 434.2	-254.9	0.0017	0.033						
DNM3	7907370	1	51.6 ± 11.7	62.1 ± 17.4	10.6	0.0017	0.033	cg21450888	Body; Open sea	70.8 ± 5.7	75.0 ± 8.4	4.2	0.041
								cg10769535	Body; Open sea	66.4 ± 4.4	71.5 ± 3.7	5.1	0.001
GP2	7999920	16	661.9 ± 420.1	475.1 ± 388.5	-186.8	0.0012	0.027						
GPRC5B	7999909	16	796.9 ± 322.0	586.9 ± 262.5	-210.0	0.0002	0.013						
GRB14	8056327	2	97.5 ± 15.5	81.5 ± 24.1	-16.0	0.0024	0.041						
HNF4G	8146986	8	123.1 ± 43.5	99.8 ± 32.8	-23.3	0.0005	0.017						
ITPR2	7961900	12	204.8 ± 36.8	172.7 ± 38.7	-32.1	0.0005	0.017						
KLF9	8161648	9	230.1 ± 29.3	211.0 ± 28.2	-19.1	0.0012	0.027						
LAMA2	8121949	6	73.7 ± 37.0	57.7 ± 20.2	-16.0	0.0034	0.049	cg15002362	Body; Open sea	49.5 ± 6.4	52.9 ± 7.7	3.4	0.014
								cg23621912	Body; Open sea	36.5 ± 4.7	40.0 ± 3.6	3.5	0.003
OLFM4	7969288	13	6,063.4 ± 1,604.4	4,926.3 ± 2,045.2	-1,137.1	0.0024	0.041						
PCSK1	8113234	5	4,238.9 ± 1,705.4	5,381.2 ± 1,765.8	1,142.3	0.0002	0.013						
RARB	8078286	3	83.4 ± 20.4	51.8 ± 17.7	-31.6	0.0002	0.013						
RASAL2	7907611	1	114.5 ± 20.7	101.4 ± 14.8	-13.1	0.0017	0.033	cg00112588	TSS1500; N Shore	53.7 ± 4.6	56.7 ± 3.7	3.0	0.017
								cg22685215	Body; Open sea	56.7 ± 5.4	60.0 ± 5.8	3.4	0.024
RPL27A	7938295	11	145.9 ± 40.1	124.9 ± 30.5	-21.0	0.0034	0.049						
UBE2E3	8046685	2	310.0 ± 22.3	276.8 ± 26.8	-33.2	0.0017	0.033						
