# Supplementary material for: Effects of palmitate on genome-wide mRNA expression and DNA methylation patterns in human pancreatic islets
Source: BMC Med. 2014 Jun 23;12:103. doi: 10.1186/1741-7015-12-103 (PMC4065864; doi:10.1186/1741-7015-12-103)
Supplement: Additional file 9: Table S9 — Global DNA methylation in human pancreatic islets exposed to palmitate compared to control treatment. Average DNA methylation (%) in gene- and CpG-regions in human pancreatic islets treated for 48 h with control media or palmitate-containing media. [file 1741-7015-12-103-S9.pdf]

Supplementary Table 9: Global DNA methylation in human pancreatic islets. Average DNA methylation (%) in gene- and CpG-regions in human pancreatic islets treated for 48 h with control media or palmitate containing media.

Relation to gene region					
**Region**	**Control mean ± sd (%)**	**Palmitate mean ± sd (%)**	**Diff. palmitate - control**	***P*-value**	***q*-value**
TSS1500	27.2 ± 1.3	27.9 ± 1.2	0.8	0.0032	0.0056
TSS200	12.2 ± 0.7	12.4 ± 0.7	0.2	0.0767	0.0895
5'UTR	22.7 ± 1.1	23.3 ± 1.0	0.6	0.0151	0.0211
1stExon	13.9 ± 0.9	14.1 ± 0.8	0.3	0.0948	0.0948
Body	59.7 ± 1.3	60.7 ± 0.5	1.0	0.0011	0.0025
3'UTR	70.7 ± 1.3	71.8 ± 0.5	1.0	0.0003	0.0018
Intergenic	56.9 ± 1.4	58.0 ± 0.8	1.1	0.0011	0.0025

Relation to CpG island region					
**Region**	**Control mean ± sd (%)**	**Palmitate mean ± sd (%)**	**Diff. palmitate - control**	***P*-value**	***q*-value**
N Shelf	72.5 ± 1.3	73.5 ± 0.6	1.0	0.0022	0.0026
N Shore	41.9 ± 1.5	43.0 ± 1.1	1.1	0.0009	0.0026
Island	14.7 ± 0.8	15.0 ± 0.7	0.2	0.1189	0.1189
S Shore	40.6 ± 1.5	41.7 ± 1.1	1.1	0.0010	0.0026
S Shelf	73.1 ± 1.3	74.1 ± 0.6	1.0	0.0022	0.0026
Open Sea	68.7 ± 1.3	69.7 ± 0.6	1.1	0.0013	0.0026
