# Supplementary material for: Effects of palmitate on genome-wide mRNA expression and DNA methylation patterns in human pancreatic islets
Source: BMC Med. 2014 Jun 23;12:103. doi: 10.1186/1741-7015-12-103 (PMC4065864; doi:10.1186/1741-7015-12-103)
Supplement: Additional file 11: Table S11 — Differential mRNA expression (q <0.05) of genes with a corresponding change in DNA methylation (P <0.05 and absolute DNA methylation difference ≥3%) of the nearest gene, in human pancreatic islets exposed to palmitate versus control. [file 1741-7015-12-103-S11.pdf]

Supplementary Table 11: Differential mRNA expression (q <0.05) of genes with a corresponding change in DNA methylation (P <0.05 and absolute DNA methylation difference ≥3%) of the nearest gene, in human pancreatic islets exposed to palmitate versus control.

Gene symbol	Chr	mRNA expression						DNA methylation (%)						
		Probe ID	Control mean ± sd	Palmitate mean ± sd	Diff. palmitate - control	*P*-value	*q*-value	Probe ID	Gene region	CpG island region	Control mean ± sd	Palmitate mean ± sd	Diff. palmitate - control	*P*-value
ABLIM1	10	7936463	494.1 ± 130.0	427.5 ± 97.7	-66.6	0.0002	0.013	cg19226626	Body	Open sea	71.7 ± 6.3	75.4 ± 5.4	3.7	0.0374
ACSS3	12	7957386	141.6 ± 57.9	105.4 ± 44.3	-36.2	0.0002	0.013	cg19981475	Body	Open sea	49.5 ± 11.4	53.6 ± 12.9	4.1	0.0477
ADAM19	5	8115490	46.1 ± 8.8	38.4 ± 4.8	-7.6	0.0017	0.033	cg07257747	Body	Open sea	58.8 ± 3.8	62.1 ± 5.3	3.4	0.0253
ADAM28	8	8145293	96.6 ± 48.4	66.5 ± 28.9	-30.1	0.0002	0.013	cg22915945	1stExon; 5'UTR	Open sea	53.8 ± 5.3	57.2 ± 4.4	3.4	0.0014
ADAMTS12	5	8111387	163.0 ± 84.2	108.9 ± 40.9	-54.1	0.0002	0.013	cg26077446	Body	Open sea	68.4 ± 3.5	72.0 ± 3.3	3.6	0.0011
ADAMTS5	21	8069689	42.9 ± 8.1	51.6 ± 12.0	8.7	0.0012	0.027	cg07771160	Body	N Shore	60.5 ± 4.6	63.8 ± 5.2	3.3	0.0124
ADAR	1	7920531	585.3 ± 80.8	544.2 ± 78.2	-41.2	0.0012	0.027	cg27530370	5'UTR	Open sea	44.9 ± 6.6	48.2 ± 4.5	3.3	0.0100
ADCY2	5	8104394	75.6 ± 10.1	116.1 ± 31.0	40.5	0.0002	0.013	cg05921947	Body	Open sea	64.9 ± 2.8	69.3 ± 3.6	4.4	0.0019
ADD3	10	7930380	417.9 ± 57.4	337.1 ± 51.4	-80.8	0.0002	0.013	cg14742187	5'UTR	Open sea	71.8 ± 4.6	74.9 ± 4.7	3.1	0.0047
ADD3	10	7930380	417.9 ± 57.4	337.1 ± 51.4	-80.8	0.0002	0.013	cg22666103	5'UTR	Open sea	54.0 ± 7.4	57.6 ± 7.5	3.7	0.0168
ALB	4	8095628	2,647.2 ± 1,479.3	1,986.9 ± 1,467.5	-660.3	0.0007	0.020	cg24656976	Body	Open sea	76.9 ± 4.4	80.4 ± 2.6	3.5	0.0076
ALDH1A3	15	7986446	624.8 ± 244.8	396.3 ± 203.3	-228.5	0.0002	0.013	cg26333136	TSS1500	Island	13.8 ± 6.6	10.2 ± 2.9	-3.6	0.0052
ANK3	10	7933772	69.4 ± 14.4	56.3 ± 11.2	-13.1	0.0005	0.017	cg24332570	Body	Open sea	72.3 ± 4.6	75.6 ± 3.7	3.3	0.0167
ANK3	10	7933772	69.4 ± 14.4	56.3 ± 11.2	-13.1	0.0005	0.017	cg02573152	Body	Open sea	66.6 ± 7.3	70.4 ± 5.2	3.8	0.0082
ANTXR1	2	8042439	214.1 ± 99.9	167.6 ± 67.5	-46.5	0.0005	0.017	cg16190888	Body	Open sea	55.7 ± 6.6	59.2 ± 6.5	3.5	0.0221
ANTXR2	4	8101260	140.3 ± 32.6	112.5 ± 34.5	-27.8	0.0024	0.041	cg03164800	Body	Open sea	69.9 ± 4.1	73.1 ± 4.8	3.2	0.0020
ANTXR2	4	8101260	140.3 ± 32.6	112.5 ± 34.5	-27.8	0.0024	0.041	cg01174264	Body	N Shore	44.7 ± 5.1	49.2 ± 4.6	4.5	0.0019
AP3S1	5	8107421	237.2 ± 28.6	209.2 ± 26.9	-28.0	0.0024	0.041	cg16627211	Body	Open sea	71.7 ± 5.0	75.1 ± 3.3	3.4	0.0080
AP3S1	5	8107421	237.2 ± 28.6	209.2 ± 26.9	-28.0	0.0024	0.041	cg24770230	Body	Open sea	77.1 ± 4.8	81.9 ± 4.3	4.7	0.0050

ARG2	14	7975268	331.4 ± 163.6	436.4 ± 198.9	105.1	0.0017	0.033	cg11752250	Body	S Shelf	64.0 ± 6.0	68.0 ± 7.0	4.0	0.0029
ARHGAP26	5	8108873	164.9 ± 32.5	135.4 ± 32.8	-29.5	0.0002	0.013	cg05153380	Body	Open sea	58.3 ± 5.5	61.6 ± 5.0	3.3	0.0094
ARHGAP26	5	8108873	164.9 ± 32.5	135.4 ± 32.8	-29.5	0.0002	0.013	cg15368044	Body	Open sea	71.2 ± 4.6	75.2 ± 3.6	4.0	0.0087
ARHGAP26	5	8108873	164.9 ± 32.5	135.4 ± 32.8	-29.5	0.0002	0.013	cg00997251	Body	Open sea	62.9 ± 5.2	67.3 ± 6.5	4.4	0.0038
ARHGAP26	5	8108873	164.9 ± 32.5	135.4 ± 32.8	-29.5	0.0002	0.013	cg17262755	Body	Open sea	53.7 ± 4.9	59.5 ± 3.7	5.9	0.0002
ARHGEF18	19	8025142	206.0 ± 39.5	192.7 ± 40.3	-13.3	0.0005	0.017	cg08036804	5'UTR; TSS1500	N Shore	67.8 ± 7.4	70.9 ± 7.5	3.1	0.0196
ARHGEF3	3	8088247	394.8 ± 100.2	328.5 ± 99.9	-66.3	0.0024	0.041	cg04389058	Body	Open sea	52.3 ± 9.1	55.8 ± 10.4	3.5	0.0076
ARID5B	10	7927732	408.8 ± 52.4	495.3 ± 86.6	86.4	0.0024	0.041	cg22782105	Body	Open sea	78.0 ± 2.9	81.2 ± 2.4	3.3	0.0062
ARID5B	10	7927732	408.8 ± 52.4	495.3 ± 86.6	86.4	0.0024	0.041	cg27027230	Body	Open sea	52.7 ± 6.4	56.2 ± 6.1	3.5	0.0349
ARID5B	10	7927732	408.8 ± 52.4	495.3 ± 86.6	86.4	0.0024	0.041	cg11283402	Body	Open sea	78.0 ± 4.4	82.0 ± 2.6	4.0	0.0023
ARID5B	10	7927732	408.8 ± 52.4	495.3 ± 86.6	86.4	0.0024	0.041	cg02863179	Body	Open sea	50.5 ± 7.5	55.8 ± 6.1	5.4	0.0001
ARL6IP5	3	8080926	3,148.3 ± 229.4	2,819.0 ± 313.9	-329.3	0.0012	0.027	cg11421073	3'UTR	Open sea	67.2 ± 3.9	71.0 ± 3.8	3.8	0.0036
ARNT	1	7919825	439.5 ± 37.7	404.0 ± 42.8	-35.5	0.0005	0.017	cg12912293	TSS1500	S Shore	70.4 ± 4.8	73.6 ± 3.1	3.2	0.0255
ASB9	X	8171392	174.5 ± 99.1	96.7 ± 52.8	-77.7	0.0002	0.013	cg13824755	Body	Open sea	79.9 ± 5.2	83.1 ± 3.5	3.2	0.0025
ASB9	X	8171392	174.5 ± 99.1	96.7 ± 52.8	-77.7	0.0002	0.013	cg01540916	TSS200; TSS1500	Open sea	39.8 ± 6.6	43.0 ± 9.4	3.2	0.0461
ATP11A	13	7970162	184.9 ± 37.8	153.7 ± 35.7	-31.2	0.0017	0.033	cg02738374	Body	Island	80.0 ± 3.7	83.4 ± 2.7	3.4	0.0007
ATP11A	13	7970162	184.9 ± 37.8	153.7 ± 35.7	-31.2	0.0017	0.033	cg19293163	Body	N Shore	63.9 ± 7.4	68.4 ± 5.6	4.5	0.0049
ATP11C	X	8175492	95.6 ± 24.0	80.0 ± 18.0	-15.6	0.0005	0.017	cg18433694	TSS200	Open sea	73.1 ± 5.9	76.1 ± 7.8	3.0	0.0059
ATP7A	X	8168472	198.0 ± 23.2	172.2 ± 33.9	-25.8	0.0024	0.041	cg26358122	5'UTR	Open sea	68.9 ± 7.6	72.3 ± 4.7	3.4	0.0130
ATP7A	X	8168472	198.0 ± 23.2	172.2 ± 33.9	-25.8	0.0024	0.041	cg22396161	3'UTR	Open sea	66.4 ± 9.6	70.0 ± 9.5	3.6	0.0164
B2M	15	7983360	5,372.3 ± 635.7	4,947.1 ± 759.7	-425.2	0.0017	0.033	cg18555073	TSS1500	N Shore	36.2 ± 5.3	39.4 ± 4.9	3.2	0.0331
BCO2	11	7943867	80.6 ± 14.5	68.9 ± 17.6	-11.7	0.0034	0.049	cg24002907	3'UTR	Open sea	28.2 ± 4.0	31.6 ± 4.7	3.3	0.0255
BGN	X	8170648	472.3 ± 232.4	374.8 ± 151.2	-97.5	0.0017	0.033	cg16847374	Body	Open sea	66.2 ± 5.9	69.5 ± 3.8	3.4	0.0079
BICC1	10	7927681	464.7 ± 189.0	351.0 ± 149.7	-113.7	0.0002	0.013	cg02387380	Body	Open sea	78.0 ± 4.7	81.9 ± 4.7	3.9	0.0054
BMPR1A	10	7928855	675.8 ± 67.7	618.1 ± 101.2	-57.7	0.0024	0.041	cg08626436	5'UTR	Open sea	22.5 ± 5.2	25.8 ± 5.2	3.3	0.0042

C11orf54	11	7943162	770.8 ± 161.3	677.3 ± 130.1	-93.5	0.0002	0.013	cg13528726	3'UTR	Open sea	84.3 ± 2.6	87.4 ± 1.7	3.1	0.0012
C20orf26	20	8061272	36.7 ± 9.4	45.4 ± 16.6	8.6	0.0034	0.049	cg11382945	TSS200; 5'UTR	S Shelf	50.5 ± 4.1	54.5 ± 4.3	4.0	0.0247
C22orf42	22	8075555	189.5 ± 84.5	234.9 ± 104.0	45.4	0.0024	0.041	cg01558877	TSS200	Open sea	42.0 ± 5.8	47.4 ± 7.0	5.4	0.0003
C3AR1	12	7960874	53.0 ± 16.8	42.5 ± 12.7	-10.5	0.0034	0.049	cg25757697	TSS1500	Open sea	77.4 ± 3.2	80.6 ± 2.9	3.1	0.0081
C4orf19	4	8094550	180.0 ± 41.5	147.7 ± 31.9	-32.3	0.0005	0.017	cg12209861	5'UTR	S Shelf	60.2 ± 6.1	64.4 ± 5.5	4.2	0.0019
C6orf27	6	8125072	116.3 ± 24.8	145.1 ± 33.3	28.8	0.0005	0.017	cg00686197	Body	Open sea	67.0 ± 5.4	70.2 ± 4.0	3.1	0.0086
C9orf5	9	8163086	965.6 ± 74.8	840.0 ± 62.4	-125.6	0.0002	0.013	cg11727569	TSS1500	S Shore	53.3 ± 5.0	57.6 ± 4.2	4.4	0.0020
CARS	11	7945803	321.5 ± 39.5	371.7 ± 47.0	50.3	0.0012	0.027	cg14935025	Body; 5'UTR	Open sea	55.3 ± 11.0	58.6 ± 11.4	3.3	0.0163
CASP1	11	7951397	62.0 ± 22.2	44.0 ± 13.9	-18.1	0.0024	0.041	cg05671385	Body	Open sea	59.0 ± 5.7	63.0 ± 6.5	4.0	0.0181
CASP5	11	7951385	29.7 ± 13.3	20.2 ± 8.0	-9.5	0.0012	0.027	cg24432977	Body; 3'UTR	Open sea	38.6 ± 10.4	42.8 ± 10.7	4.2	0.0191
CBR4	4	8103622	483.1 ± 70.9	391.5 ± 98.7	-91.6	0.0002	0.013	cg26174928	Body	N Shore	76.6 ± 5.2	79.8 ± 4.9	3.1	0.0263
CCDC149	4	8099670	118.3 ± 10.4	134.5 ± 9.0	16.2	0.0005	0.017	cg06102690	TSS200	Open sea	18.6 ± 4.9	22.2 ± 4.9	3.6	0.0025
CCNA2	4	8102643	89.1 ± 21.8	61.6 ± 11.4	-27.5	0.0002	0.013	cg23574427	TSS1500	S Shore	68.9 ± 4.3	72.2 ± 5.0	3.2	0.0096
CD200R1	3	8089568	9.0 ± 1.1	10.1 ± 0.8	1.1	0.0034	0.049	cg00246486	3'UTR	Open sea	63.6 ± 5.2	68.7 ± 3.4	5.1	0.0011
CD2AP	6	8120102	1,048.2 ± 126.6	944.1 ± 134.8	-104.1	0.0007	0.020	cg20172563	Body	Open sea	42.9 ± 5.4	46.0 ± 5.3	3.1	0.0321
CD2AP	6	8120102	1,048.2 ± 126.6	944.1 ± 134.8	-104.1	0.0007	0.020	cg19989043	Body	Open sea	61.3 ± 5.7	64.5 ± 4.6	3.1	0.0077
CD2AP	6	8120102	1,048.2 ± 126.6	944.1 ± 134.8	-104.1	0.0007	0.020	cg15629447	3'UTR	Open sea	75.9 ± 5.5	80.1 ± 4.5	4.1	0.0039
CDK2AP1	12	7967412	1,125.2 ± 132.0	953.3 ± 103.5	-171.9	0.0005	0.017	cg22465824	3'UTR	Open sea	76.4 ± 4.0	79.7 ± 2.1	3.3	0.0106
CDK5	7	8143850	255.7 ± 35.6	232.3 ± 36.9	-23.4	0.0012	0.027	cg11368578	1stExon; 5'UTR	Island	14.6 ± 6.5	18.9 ± 7.4	4.3	0.0129
CHEK1	11	7945014	73.8 ± 20.0	52.0 ± 16.7	-21.8	0.0002	0.013	cg00554702	5'UTR	S Shore	11.6 ± 3.3	14.6 ± 5.1	3.0	0.0007
CLIC5	6	8126729	34.8 ± 10.1	24.6 ± 4.3	-10.2	0.0007	0.020	cg23716866	TSS1500	Open sea	62.5 ± 3.7	66.5 ± 5.1	4.0	0.0074
CLMN	14	7981142	304.8 ± 101.3	243.9 ± 91.5	-60.9	0.0002	0.013	cg05239310	3'UTR	Open sea	47.9 ± 6.6	50.9 ± 5.2	3.0	0.0248
CLRN3	10	7937016	53.6 ± 32.7	24.8 ± 8.7	-28.8	0.0002	0.013	cg23817637	TSS1500	Open sea	72.9 ± 6.7	76.7 ± 4.3	3.9	0.0118
CLYBL	13	7969815	112.1 ± 15.7	101.3 ± 13.6	-10.8	0.0007	0.020	cg17826893	Body	Open sea	67.5 ± 6.7	70.6 ± 6.7	3.1	0.0256
CLYBL	13	7969815	112.1 ± 15.7	101.3 ± 13.6	-10.8	0.0007	0.020	cg02509730	Body	Island	68.5 ± 5.7	72.3 ± 7.4	3.8	0.0106

CNGA3	2	8043782	114.5 ± 44.8	190.1 ± 79.9	75.6	0.0002	0.013	cg05564412	TSS1500	N Shore	67.2 ± 7.3	70.6 ± 8.1	3.4	0.0114
COL15A1	9	8156783	302.3 ± 109.1	249.3 ± 87.1	-53.0	0.0034	0.049	cg14259208	Body	Open sea	70.4 ± 3.1	74.1 ± 5.0	3.8	0.0070
COL4A1	13	7972750	467.7 ± 171.7	366.4 ± 106.8	-101.3	0.0024	0.041	cg02730492	Body	Open sea	69.4 ± 4.2	72.8 ± 4.3	3.3	0.0139
COL4A1	13	7972750	467.7 ± 171.7	366.4 ± 106.8	-101.3	0.0024	0.041	cg19234894	Body	Open sea	67.1 ± 6.3	71.2 ± 5.6	4.2	0.0275
COPG	3	8082478	699.6 ± 104.0	781.6 ± 110.1	82.0	0.0024	0.041	cg12216435	Body	N Shore	62.6 ± 4.4	65.7 ± 5.1	3.1	0.0090
CRIM1	2	8041447	552.1 ± 129.2	498.8 ± 124.7	-53.3	0.0007	0.020	cg11913694	Body	Open sea	79.0 ± 4.2	82.6 ± 3.8	3.7	0.0072
CRMP1	4	8099172	248.5 ± 80.3	272.9 ± 67.4	24.4	0.0034	0.049	cg20529645	Body	N Shelf	68.5 ± 4.7	71.7 ± 3.5	3.2	0.0363
CRMP1	4	8099172	248.5 ± 80.3	272.9 ± 67.4	24.4	0.0034	0.049	cg05737153	Body	Open sea	69.7 ± 6.3	73.4 ± 7.0	3.6	0.0136
CRMP1	4	8099172	248.5 ± 80.3	272.9 ± 67.4	24.4	0.0034	0.049	cg23830540	Body	Open sea	38.9 ± 13.0	42.9 ± 13.2	4.0	0.0150
CSTF1	20	8063484	188.8 ± 9.9	175.5 ± 11.4	-13.4	0.0012	0.027	cg15747531	Body	S Shelf	76.4 ± 6.6	79.6 ± 5.7	3.1	0.0101
CTSC	11	7950906	312.2 ± 81.0	225.5 ± 52.2	-86.7	0.0007	0.020	cg03596479	Body	Open sea	66.7 ± 2.9	70.5 ± 4.5	3.8	0.0014
CXXC5	5	8108447	739.8 ± 60.3	645.7 ± 82.9	-94.1	0.0005	0.017	cg17306279	5'UTR	Island	41.4 ± 6.1	38.3 ± 7.0	-3.0	0.0409
CXXC5	5	8108447	739.8 ± 60.3	645.7 ± 82.9	-94.1	0.0005	0.017	cg06905511	3'UTR	S Shore	81.8 ± 2.3	85.5 ± 2.9	3.7	0.0003
CYFIP1	15	7981824	478.1 ± 60.7	436.7 ± 59.2	-41.3	0.0005	0.017	cg02594498	5'UTR	Island	12.2 ± 5.6	15.6 ± 6.4	3.4	0.0233
DCBLD2	3	8089082	554.9 ± 156.9	428.9 ± 107.9	-126.0	0.0005	0.017	cg16331862	3'UTR	Open sea	69.2 ± 8.5	73.8 ± 7.3	4.6	0.0038
DCDC2	6	8124196	483.2 ± 135.9	391.0 ± 132.8	-92.2	0.0024	0.041	cg04945158	TSS1500	N Shore	30.4 ± 3.5	34.8 ± 4.8	4.5	0.0031
DDAH1	1	7917347	710.1 ± 122.2	586.1 ± 79.9	-123.9	0.0002	0.013	cg01550348	Body	Open sea	60.8 ± 11.4	64.0 ± 12.5	3.2	0.0437
DDX5	17	8017634	2,163.1 ± 291.2	2,023.7 ± 233.2	-139.3	0.0017	0.033	cg23225508	Body	N Shore	38.9 ± 5.6	42.6 ± 4.9	3.7	0.0028
DDX5	17	8017634	2,163.1 ± 291.2	2,023.7 ± 233.2	-139.3	0.0017	0.033	cg14548777	3'UTR	S Shore	55.1 ± 6.5	58.7 ± 6.0	3.7	0.0115
DDX5	17	8017634	2,163.1 ± 291.2	2,023.7 ± 233.2	-139.3	0.0017	0.033	cg10244222	Body	S Shelf	70.5 ± 2.4	74.3 ± 4.1	3.8	0.0021
DHX15	4	8099649	1,318.2 ± 137.1	1,222.2 ± 117.0	-96.0	0.0024	0.041	cg21302120	Body	N Shore	14.9 ± 8.2	18.6 ± 8.0	3.7	0.0288
DNAJA4	15	7985147	318.3 ± 94.8	417.0 ± 127.4	98.7	0.0005	0.017	cg21642988	TSS200; Body	S Shore	49.0 ± 9.7	52.4 ± 10.5	3.4	0.0322
DNAJB6	7	8137627	373.7 ± 32.1	328.2 ± 40.6	-45.5	0.0017	0.033	cg05090392	3'UTR	S Shore	79.2 ± 3.5	83.4 ± 3.4	4.3	0.0002
DNM3	1	7907370	51.6 ± 11.7	62.1 ± 17.4	10.6	0.0017	0.033	cg21450888	Body	Open sea	70.8 ± 5.7	75.0 ± 8.4	4.2	0.0415
DNM3	1	7907370	51.6 ± 11.7	62.1 ± 17.4	10.6	0.0017	0.033	cg10769535	Body	Open sea	66.4 ± 4.4	71.5 ± 3.7	5.1	0.0013

DSE	6	8121588	237.1 ± 66.9	194.5 ± 49.5	-42.6	0.0005	0.017	cg24407607	Body	Open sea	49.9 ± 23.0	52.9 ± 23.1	3.0	0.0106
DSE	6	8121588	237.1 ± 66.9	194.5 ± 49.5	-42.6	0.0005	0.017	cg19341652	TSS1500	N Shore	17.8 ± 3.1	20.9 ± 3.0	3.1	0.0021
DUSP5	10	7930413	357.0 ± 61.4	471.4 ± 66.0	114.4	0.0005	0.017	cg18756771	Body	S Shelf	52.5 ± 6.3	55.6 ± 4.7	3.1	0.0145
EFR3A	8	8148333	1,179.9 ± 218.9	1,071.9 ± 203.8	-108.0	0.0005	0.017	cg24184792	Body	S Shelf	68.7 ± 7.9	71.7 ± 6.5	3.1	0.0140
ENAH	1	7924619	345.3 ± 75.1	278.0 ± 58.4	-67.3	0.0002	0.013	cg21410954	Body	Open sea	51.0 ± 13.7	54.9 ± 12.5	3.8	0.0124
ENG	9	8164269	268.5 ± 66.3	211.6 ± 47.4	-56.8	0.0012	0.027	cg13458609	Body	Open sea	43.8 ± 6.6	40.7 ± 8.7	-3.2	0.0298
ENOX1	13	7971311	45.9 ± 4.5	52.9 ± 7.2	7.0	0.0034	0.049	cg06587495	5'UTR	Open sea	79.7 ± 3.9	82.8 ± 3.2	3.1	0.0195
ENOX1	13	7971311	45.9 ± 4.5	52.9 ± 7.2	7.0	0.0034	0.049	cg09603398	5'UTR	Open sea	74.8 ± 6.0	77.9 ± 3.3	3.1	0.0206
ENOX1	13	7971311	45.9 ± 4.5	52.9 ± 7.2	7.0	0.0034	0.049	cg25544384	5'UTR	Open sea	76.4 ± 4.0	79.6 ± 4.1	3.2	0.0149
ENOX1	13	7971311	45.9 ± 4.5	52.9 ± 7.2	7.0	0.0034	0.049	cg04417236	Body	Open sea	37.1 ± 3.9	42.8 ± 7.4	5.6	0.0024
EPB41L1	20	8062251	122.0 ± 15.5	110.9 ± 15.5	-11.1	0.0012	0.027	cg21211882	Body	Open sea	66.5 ± 4.7	69.7 ± 3.9	3.2	0.0074
EPB41L4A	5	8113512	120.2 ± 25.7	95.5 ± 26.5	-24.7	0.0005	0.017	cg24942990	3'UTR	S Shelf	76.6 ± 4.6	79.7 ± 3.6	3.1	0.0055
EPB41L4A	5	8113512	120.2 ± 25.7	95.5 ± 26.5	-24.7	0.0005	0.017	cg14798584	Body	Open sea	59.9 ± 3.2	63.7 ± 4.4	3.8	0.0058
ERCC6	10	7933509	191.3 ± 23.6	174.4 ± 31.8	-16.9	0.0007	0.020	cg25656283	Body	Open sea	64.6 ± 4.9	68.5 ± 5.9	3.9	0.0045
EXOC4	7	8136293	623.4 ± 56.3	579.0 ± 64.4	-44.4	0.0005	0.017	cg10939418	Body	Open sea	70.4 ± 7.2	73.9 ± 3.6	3.5	0.0233
F2R	5	8106393	194.1 ± 101.6	138.6 ± 51.2	-55.5	0.0007	0.020	cg09059904	Body	S Shore	30.9 ± 3.9	34.4 ± 3.8	3.5	0.0123
FAM13C	10	7933733	67.7 ± 31.5	78.4 ± 34.0	10.7	0.0012	0.027	cg04557025	Body; 5'UTR	Open sea	78.5 ± 5.2	81.6 ± 4.6	3.2	0.0187
FARP1	13	7969736	263.1 ± 50.1	232.7 ± 40.9	-30.4	0.0005	0.017	cg20653375	3'UTR	Open sea	48.3 ± 5.8	51.8 ± 5.2	3.5	0.0185
FARP1	13	7969736	263.1 ± 50.1	232.7 ± 40.9	-30.4	0.0005	0.017	cg16689193	Body	Open sea	76.1 ± 3.0	80.0 ± 4.7	3.9	0.0019
FBXL4	6	8128356	279.8 ± 39.4	249.3 ± 29.1	-30.5	0.0017	0.033	cg19140262	5'UTR	Open sea	49.1 ± 13.2	52.5 ± 12.6	3.4	0.0307
FBXO34	14	7974473	420.0 ± 45.1	360.4 ± 39.0	-59.6	0.0012	0.027	cg04971161	5'UTR	Open sea	69.2 ± 5.1	72.4 ± 3.5	3.1	0.0304
FGF13	X	8175444	110.0 ± 24.7	138.0 ± 33.3	28.1	0.0002	0.013	cg03537374	5'UTR; Body; TSS1500	Open sea	59.0 ± 7.8	62.2 ± 5.7	3.2	0.0275
FGF13	X	8175444	110.0 ± 24.7	138.0 ± 33.3	28.1	0.0002	0.013	cg02588867	5'UTR; Body; TSS1500	Open sea	76.0 ± 5.0	79.3 ± 4.0	3.4	0.0101
FGF19	11	7950023	202.8 ± 119.0	117.5 ± 43.1	-85.3	0.0012	0.027	cg02513449	TSS1500	S Shore	39.7 ± 5.8	43.3 ± 5.9	3.6	0.0046

FHL2	2	8054377	152.2 ± 36.7	129.6 ± 27.1	-22.6	0.0007	0.020	cg01007821	5'UTR	S Shelf	75.4 ± 6.4	78.8 ± 6.1	3.4	0.0281
FN1	2	8058765	1,679.4 ± 750.8	1,213.2 ± 636.7	-466.2	0.0012	0.027	cg09335314	Body	Open sea	79.1 ± 5.1	82.1 ± 2.7	3.0	0.0190
FN1	2	8058765	1,679.4 ± 750.8	1,213.2 ± 636.7	-466.2	0.0012	0.027	cg25099021	Body	Open sea	78.8 ± 7.3	82.9 ± 3.2	4.1	0.0397
FRAS1	4	8095907	58.9 ± 13.8	48.0 ± 12.8	-10.9	0.0002	0.013	cg14091154	Body	Open sea	72.2 ± 4.8	76.0 ± 4.1	3.9	0.0127
GAB2	11	7950671	96.8 ± 11.9	110.3 ± 11.9	13.4	0.0034	0.049	cg05447955	Body	N Shelf	78.6 ± 3.9	84.1 ± 2.9	5.5	0.00003
GALNT5	2	8045835	59.0 ± 18.4	38.8 ± 8.6	-20.2	0.0012	0.027	cg10982635	Body	Open sea	47.4 ± 13.0	51.0 ± 12.6	3.6	0.0174
GBP2	1	7917532	143.4 ± 63.3	105.1 ± 53.0	-38.3	0.0017	0.033	cg13187849	Body	Open sea	75.3 ± 3.4	78.4 ± 2.8	3.1	0.0136
GCH1	14	7979269	722.3 ± 248.2	1,050.1 ± 404.9	327.7	0.0005	0.017	cg16387581	Body	Open sea	74.3 ± 4.8	77.3 ± 4.7	3.0	0.0211
GCH1	14	7979269	722.3 ± 248.2	1,050.1 ± 404.9	327.7	0.0005	0.017	cg01899130	Body	Open sea	29.1 ± 11.0	32.4 ± 11.2	3.4	0.0440
GCKR	2	8040960	80.8 ± 32.2	104.2 ± 65.0	23.4	0.0012	0.027	cg20229788	TSS1500	S Shore	24.3 ± 6.3	27.4 ± 5.9	3.1	0.0129
GFRA1	10	7936494	57.9 ± 9.8	72.7 ± 14.5	14.8	0.0005	0.017	cg17718728	Body	Open sea	57.7 ± 12.0	60.7 ± 11.0	3.0	0.0422
GIT2	12	7966268	298.0 ± 19.8	269.8 ± 28.6	-28.2	0.0002	0.013	cg17337670	Body	Open sea	64.2 ± 6.7	67.9 ± 6.6	3.7	0.0024
GLIPR1	12	7957260	213.6 ± 104.2	153.3 ± 54.1	-60.3	0.0012	0.027	cg01554451	TSS1500	Open sea	67.4 ± 5.0	71.5 ± 4.0	4.1	0.0025
GLIS3	9	8159900	441.4 ± 66.2	394.9 ± 47.8	-46.5	0.0012	0.027	cg14641122	Body	Open sea	33.4 ± 8.2	36.7 ± 9.3	3.2	0.0103
GLIS3	9	8159900	441.4 ± 66.2	394.9 ± 47.8	-46.5	0.0012	0.027	cg14269813	Body	Open sea	58.2 ± 11.1	61.6 ± 11.3	3.4	0.0157
GPR110	6	8126820	246.7 ± 123.7	117.4 ± 48.5	-129.2	0.0002	0.013	cg21577639	Body	Open sea	69.5 ± 6.8	73.6 ± 2.2	4.1	0.0133
GPR160	3	8083839	151.5 ± 46.6	117.4 ± 42.4	-34.1	0.0017	0.033	cg16635196	5'UTR	S Shore	51.0 ± 2.8	55.2 ± 5.8	4.2	0.0092
GUK1	1	7910241	587.1 ± 53.3	658.1 ± 79.8	71.0	0.0002	0.013	cg22521182	Body; 5'UTR; TSS1500	S Shelf	80.9 ± 3.6	84.1 ± 2.1	3.2	0.0065
HIST1H1A	6	8124380	178.7 ± 90.8	121.4 ± 32.7	-57.3	0.0012	0.027	cg10146929	1stExon	N Shelf	57.0 ± 5.5	61.0 ± 4.0	4.0	0.0235
HIST1H2AC	6	8117372	745.9 ± 192.9	660.6 ± 164.2	-85.3	0.0017	0.033	cg05804587	TSS200	N Shore	10.2 ± 2.3	13.6 ± 4.3	3.4	0.0025
HIST1H3I	6	8124531	361.5 ± 121.2	274.6 ± 78.7	-86.9	0.0012	0.027	cg25845597	TSS1500	Island	11.9 ± 6.0	15.7 ± 6.5	3.7	0.0137
HK1	10	7928019	169.7 ± 43.7	150.4 ± 43.5	-19.3	0.0024	0.041	cg04031454	TSS1500	Open sea	65.9 ± 4.4	69.1 ± 4.2	3.2	0.0038
HLA-DMB	6	8125530	183.6 ± 45.3	134.1 ± 37.9	-49.5	0.0002	0.013	cg24523259	Body	Open sea	78.9 ± 4.2	82.2 ± 2.9	3.3	0.0018
HLA-DMB	6_qbl_hap2	8180078	177.9 ± 45.0	130.1 ± 39.4	-47.9	0.0002	0.013	cg24523259	Body	Open sea	78.9 ± 4.2	82.2 ± 2.9	3.3	0.0018
HMGN1	X	8176191	939.9 ± 66.3	856.7 ± 90.5	-83.2	0.0024	0.041	cg04973239	3'UTR	Open sea	70.2 ± 4.2	74.7 ± 3.7	4.5	0.00003

ICAM2	17	8017547	86.9 ± 12.3	105.5 ± 20.3	18.6	0.0012	0.027	cg10275770	5'UTR; 1stExon	Open sea	54.0 ± 4.4	57.3 ± 4.6	3.3	0.0380
ID3	1	7913655	371.2 ± 126.9	278.3 ± 76.3	-92.9	0.0002	0.013	cg09858862	TSS1500	S Shore	73.9 ± 5.0	77.8 ± 3.7	3.8	0.0175
IER3	6_cox_hap1	8178435	1,166.2 ± 400.3	1,669.4 ± 454.9	503.1	0.0012	0.027	cg20284982	TSS1500	S Shore	44.7 ± 5.2	48.3 ± 5.3	3.6	0.0301
IER3	6	8124848	960.3 ± 311.5	1,376.0 ± 361.6	415.7	0.0012	0.027	cg20284982	TSS1500	S Shore	44.7 ± 5.2	48.3 ± 5.3	3.6	0.0301
IER3	6_qbl_hap2	8179704	960.3 ± 311.5	1,376.0 ± 361.6	415.7	0.0012	0.027	cg20284982	TSS1500	S Shore	44.7 ± 5.2	48.3 ± 5.3	3.6	0.0301
IFRD1	7	8135514	341.5 ± 97.7	458.2 ± 161.0	116.8	0.0007	0.020	cg25191332	TSS1500	Open sea	70.1 ± 5.4	73.3 ± 5.0	3.2	0.0167
IGF1R	15	7986359	361.2 ± 97.4	307.7 ± 54.7	-53.5	0.0002	0.013	cg07160602	Body	S Shelf	69.4 ± 4.1	72.7 ± 4.7	3.2	0.0016
IGF1R	15	7986359	361.2 ± 97.4	307.7 ± 54.7	-53.5	0.0002	0.013	cg12398448	Body	S Shore	77.3 ± 4.6	81.4 ± 3.6	4.1	0.0033
IGF1R	15	7986359	361.2 ± 97.4	307.7 ± 54.7	-53.5	0.0002	0.013	cg05110803	Body	Open sea	75.5 ± 5.0	79.6 ± 4.8	4.1	0.0085
IGF1R	15	7986359	361.2 ± 97.4	307.7 ± 54.7	-53.5	0.0002	0.013	cg24122407	Body	Open sea	66.8 ± 8.7	71.2 ± 9.2	4.4	0.0084
IGF1R	15	7986383	474.7 ± 140.4	386.2 ± 85.9	-88.5	0.0005	0.017	cg07160602	Body	S Shelf	69.4 ± 4.1	72.7 ± 4.7	3.2	0.0016
IGF1R	15	7986383	474.7 ± 140.4	386.2 ± 85.9	-88.5	0.0005	0.017	cg12398448	Body	S Shore	77.3 ± 4.6	81.4 ± 3.6	4.1	0.0033
IGF1R	15	7986383	474.7 ± 140.4	386.2 ± 85.9	-88.5	0.0005	0.017	cg05110803	Body	Open sea	75.5 ± 5.0	79.6 ± 4.8	4.1	0.0085
IGF1R	15	7986383	474.7 ± 140.4	386.2 ± 85.9	-88.5	0.0005	0.017	cg24122407	Body	Open sea	66.8 ± 8.7	71.2 ± 9.2	4.4	0.0084
IQGAP2	5	8106354	340.9 ± 124.2	211.5 ± 115.6	-129.4	0.0002	0.013	cg16057435	Body	Open sea	66.8 ± 5.4	70.2 ± 4.9	3.5	0.0223
IRS2	13	7972745	169.0 ± 27.3	205.6 ± 33.2	36.6	0.0012	0.027	cg08348990	Body	Open sea	74.6 ± 6.3	78.7 ± 6.8	4.0	0.0200
JAK1	1	7916747	1,423.0 ± 207.5	1,316.8 ± 189.7	-106.2	0.0024	0.041	cg02755455	5'UTR	Open sea	82.4 ± 2.6	85.6 ± 2.2	3.2	0.0004
JARID2	6	8116998	187.6 ± 36.2	159.9 ± 31.8	-27.7	0.0002	0.013	cg01769037	5'UTR; 1stExon	S Shore	9.6 ± 3.5	13.1 ± 6.4	3.5	0.0012
KBTBD10	2	8046160	616.8 ± 258.3	904.9 ± 417.8	288.1	0.0002	0.013	cg10740880	TSS1500	Open sea	75.5 ± 6.3	78.7 ± 4.8	3.2	0.0433
KCNK17	6	8126163	166.0 ± 82.9	140.6 ± 72.9	-25.5	0.0002	0.013	cg13075951	TSS200	S Shore	61.6 ± 6.9	64.8 ± 7.9	3.2	0.0100
KCNRG	13	7969166	81.5 ± 11.8	65.6 ± 13.4	-15.9	0.0034	0.049	cg17948986	Body; 3'UTR	Open sea	82.2 ± 5.6	85.5 ± 3.7	3.3	0.0135
KCTD2	17	8009737	219.6 ± 19.7	198.9 ± 16.0	-20.6	0.0005	0.017	cg09185884	Body	Open sea	51.7 ± 7.3	55.8 ± 8.3	4.1	0.0088
KIAA0182	16	7997680	256.6 ± 40.8	240.6 ± 37.4	-16.1	0.0034	0.049	cg02563600	5'UTR; Body	Island	66.0 ± 5.5	69.2 ± 3.9	3.2	0.0062
KIAA0317	14	7980189	326.5 ± 38.3	286.7 ± 47.8	-39.9	0.0012	0.027	cg01090926	Body	Open sea	70.3 ± 4.2	73.3 ± 3.0	3.0	0.0187
KIAA1033	12	7958216	840.0 ± 80.9	781.7 ± 79.4	-58.4	0.0024	0.041	cg09095792	Body	S Shelf	53.5 ± 5.8	57.1 ± 4.4	3.5	0.0248

KIAA1244	6	8122279	574.1 ± 190.3	735.4 ± 279.5	161.2	0.0012	0.027	cg11385476	Body	Open sea	53.3 ± 4.4	56.4 ± 4.5	3.1	0.0052
KIDINS220	2	8050128	991.8 ± 143.8	903.5 ± 123.0	-88.3	0.0017	0.033	cg06718139	Body	Open sea	62.0 ± 8.1	65.5 ± 7.5	3.6	0.0072
KLKB1	4	8098654	55.5 ± 42.1	40.7 ± 26.7	-14.8	0.0002	0.013	cg11597515	Body	Open sea	53.7 ± 11.2	57.3 ± 12.7	3.7	0.0168
LAMA2	6	8121949	73.7 ± 37.0	57.7 ± 20.2	-16.0	0.0034	0.049	cg15002362	Body	Open sea	49.5 ± 6.4	52.9 ± 7.7	3.4	0.0141
LAMA2	6	8121949	73.7 ± 37.0	57.7 ± 20.2	-16.0	0.0034	0.049	cg23621912	Body	Open sea	36.5 ± 4.7	40.0 ± 3.6	3.5	0.0026
LAMB1	7	8142194	608.8 ± 129.2	467.5 ± 101.8	-141.3	0.0005	0.017	cg26448609	Body	Open sea	73.1 ± 3.8	76.4 ± 2.7	3.3	0.0058
LAMC1	1	7908041	791.0 ± 144.6	662.8 ± 113.6	-128.2	0.0002	0.013	cg22809683	Body	Open sea	59.6 ± 5.4	63.6 ± 6.4	4.1	0.0027
LANCL1	2	8058614	437.1 ± 43.3	376.6 ± 56.6	-60.5	0.0017	0.033	cg13257412	Body	N Shore	13.0 ± 2.3	16.7 ± 7.0	3.7	0.0047
LASS6	2	8046086	1,299.1 ± 341.9	1,158.7 ± 267.3	-140.4	0.0005	0.017	cg15701842	Body	Open sea	72.8 ± 4.1	76.1 ± 3.5	3.3	0.0035
LGR4	11	7947199	841.3 ± 239.0	650.1 ± 211.1	-191.2	0.0002	0.013	cg16964946	Body	Open sea	54.1 ± 6.5	57.3 ± 9.4	3.2	0.0328
LHFP	13	7971150	131.5 ± 43.5	103.5 ± 28.0	-28.0	0.0005	0.017	cg02710723	Body	Open sea	81.4 ± 4.6	84.5 ± 5.4	3.1	0.0058
LHFP	13	7971150	131.5 ± 43.5	103.5 ± 28.0	-28.0	0.0005	0.017	cg15617023	Body	Open sea	59.0 ± 6.9	63.1 ± 9.5	4.1	0.0106
LIMA1	12	7963187	1,119.1 ± 177.0	868.4 ± 164.9	-250.8	0.0002	0.013	cg00718418	TSS1500	Open sea	71.8 ± 7.0	75.2 ± 6.8	3.4	0.0271
LIMA1	12	7963187	1,119.1 ± 177.0	868.4 ± 164.9	-250.8	0.0002	0.013	cg18559571	3'UTR	Open sea	75.5 ± 3.7	78.9 ± 3.3	3.4	0.0044
LIMA1	12	7963187	1,119.1 ± 177.0	868.4 ± 164.9	-250.8	0.0002	0.013	cg05903046	Body	Open sea	68.1 ± 3.0	71.7 ± 4.1	3.6	0.0133
LIMK2	22	8072461	188.8 ± 52.0	162.4 ± 27.8	-26.4	0.0012	0.027	cg15504662	TSS200; Body	Open sea	33.3 ± 8.0	37.0 ± 11.1	3.8	0.0236
LITAF	16	7999468	946.5 ± 244.1	817.5 ± 275.8	-129.0	0.0005	0.017	cg03071793	TSS1500	S Shore	64.9 ± 4.8	69.2 ± 4.6	4.4	0.0036
LMCD1	3	8077490	164.3 ± 42.3	209.9 ± 39.7	45.6	0.0002	0.013	cg16784366	Body	Open sea	59.3 ± 6.3	63.3 ± 8.9	4.0	0.0115
LNX2	13	7970716	662.0 ± 146.2	553.7 ± 125.4	-108.3	0.0005	0.017	cg11588787	5'UTR	Open sea	30.8 ± 6.6	33.9 ± 8.3	3.0	0.0069
LOC388796	20	8066247	131.0 ± 56.0	153.2 ± 48.6	22.2	0.0024	0.041	cg11609780	Body	N Shelf	78.4 ± 2.6	81.7 ± 3.1	3.3	0.0052
LONRF1	8	8149399	234.9 ± 20.7	274.6 ± 43.7	39.7	0.0024	0.041	cg20450712	3'UTR	Open sea	78.0 ± 6.5	81.4 ± 2.8	3.3	0.0269
LPCAT2	16	7995697	114.8 ± 36.5	89.8 ± 26.5	-24.9	0.0002	0.013	cg00495811	3'UTR	Open sea	62.3 ± 8.1	65.9 ± 8.4	3.5	0.0062
LRBA	4	8103106	545.8 ± 100.6	496.8 ± 107.2	-49.0	0.0034	0.049	cg14904733	Body	S Shore	75.9 ± 5.1	79.3 ± 3.9	3.4	0.0304
LRBA	4	8103106	545.8 ± 100.6	496.8 ± 107.2	-49.0	0.0034	0.049	cg06215107	Body	Island	57.6 ± 6.0	62.5 ± 6.0	4.9	0.0057
LRRC16A	6	8117243	259.2 ± 33.4	227.8 ± 35.7	-31.3	0.0005	0.017	cg17031967	Body	Open sea	65.0 ± 5.7	68.5 ± 5.5	3.6	0.0107

LRRTM2	5	8114470	54.0 ± 15.7	82.2 ± 35.9	28.1	0.0002	0.013	cg18712348	TSS1500	Open sea	70.0 ± 4.4	74.1 ± 4.4	4.2	0.0044
LYPD1	2	8055314	260.6 ± 99.0	195.8 ± 70.1	-64.8	0.0005	0.017	cg20273260	TSS1500; TSS200	S Shore	46.0 ± 6.8	49.6 ± 7.1	3.6	0.0045
MAMLD1	X	8170420	88.6 ± 9.3	101.5 ± 13.6	13.0	0.0024	0.041	cg16440909	5'UTR; 1stExon	Open sea	68.1 ± 16.0	71.3 ± 16.7	3.2	0.0367
MAP1B	5	8106098	751.7 ± 250.1	872.0 ± 256.3	120.3	0.0024	0.041	cg21912162	Body	Open sea	60.4 ± 3.1	63.4 ± 4.3	3.1	0.0273
MAP1B	5	8106098	751.7 ± 250.1	872.0 ± 256.3	120.3	0.0024	0.041	cg15126050	Body	Open sea	75.4 ± 3.8	78.5 ± 3.4	3.1	0.0041
MAP1B	5	8106098	751.7 ± 250.1	872.0 ± 256.3	120.3	0.0024	0.041	cg17776376	Body	Open sea	81.8 ± 3.8	85.0 ± 4.4	3.3	0.0038
MAP1LC3A	20	8062023	207.5 ± 48.4	252.6 ± 70.9	45.1	0.0012	0.027	cg07486199	Body	Island	70.7 ± 5.5	73.9 ± 6.0	3.3	0.0134
MAP2K3	17	8005707	143.1 ± 23.1	187.0 ± 57.7	43.9	0.0002	0.013	cg11469540	Body	S Shore	12.0 ± 3.9	15.1 ± 4.5	3.1	0.0118
MAP6	11	7950524	133.1 ± 35.2	143.0 ± 33.2	9.9	0.0034	0.049	cg05062413	Body	Open sea	64.1 ± 5.8	67.6 ± 5.5	3.5	0.0156
MAPKAP1	9	8164177	610.6 ± 66.9	568.3 ± 65.6	-42.3	0.0012	0.027	cg14146657	5'UTR; Body	Open sea	66.9 ± 6.3	70.8 ± 7.4	4.0	0.0013
MATN2	8	8147516	166.8 ± 61.6	122.3 ± 20.2	-44.5	0.0007	0.020	cg19987349	Body	Open sea	57.2 ± 4.5	60.4 ± 3.1	3.3	0.0143
MATR3	5	8108403	952.8 ± 84.4	875.2 ± 94.2	-77.6	0.0007	0.020	cg17668981	5'UTR	Open sea	54.9 ± 6.2	58.4 ± 6.1	3.5	0.0312
MED13L	12	7966706	267.3 ± 33.8	242.3 ± 36.1	-25.1	0.0012	0.027	cg05133323	Body	Open sea	56.2 ± 5.2	59.3 ± 6.2	3.1	0.0121
MED13L	12	7966706	267.3 ± 33.8	242.3 ± 36.1	-25.1	0.0012	0.027	cg20777829	Body	Open sea	57.0 ± 6.6	60.4 ± 8.2	3.4	0.0169
MED13L	12	7966706	267.3 ± 33.8	242.3 ± 36.1	-25.1	0.0012	0.027	cg15877399	Body	Open sea	51.5 ± 10.2	56.0 ± 9.4	4.4	0.0099
MEIS1	2	8042356	700.4 ± 59.6	614.9 ± 91.3	-85.4	0.0012	0.027	cg06994420	Body	Island	30.2 ± 5.1	26.7 ± 6.8	-3.5	0.0248
MELK	9	8155214	58.3 ± 20.2	33.2 ± 9.8	-25.1	0.0002	0.013	cg13720890	Body	Open sea	84.0 ± 3.8	87.1 ± 3.1	3.1	0.0015
METTL7B	12	7956009	119.6 ± 27.9	89.7 ± 17.7	-29.9	0.0002	0.013	cg05567435	TSS1500	Open sea	56.4 ± 7.3	59.7 ± 5.2	3.3	0.0179
MGST1	12	7954196	718.5 ± 211.5	610.5 ± 156.0	-107.9	0.0017	0.033	cg18233746	TSS1500; TSS200; 5'UTR	Open sea	40.0 ± 8.2	43.4 ± 7.4	3.4	0.0076
MKNK2	19	8032392	236.1 ± 66.3	277.3 ± 126.9	41.3	0.0034	0.049	cg18418335	Body	Island	20.8 ± 5.8	16.0 ± 8.3	-4.8	0.0053
MLLT11	1	7905329	429.0 ± 175.7	504.8 ± 202.9	75.8	0.0024	0.041	cg21205392	5'UTR	S Shelf	67.0 ± 4.8	70.5 ± 5.7	3.5	0.0149
MOBKL2B	9	8160521	163.3 ± 43.3	135.9 ± 29.9	-27.4	0.0002	0.013	cg14643264	Body	Open sea	60.2 ± 8.4	63.4 ± 6.8	3.2	0.0126
MPP6	7	8131927	690.1 ± 104.8	627.5 ± 132.6	-62.6	0.0034	0.049	cg17383203	5'UTR	Open sea	64.1 ± 7.6	67.8 ± 6.6	3.7	0.0079
MTA2	11	7948696	501.4 ± 34.8	460.3 ± 38.2	-41.1	0.0007	0.020	cg01538998	3'UTR	S Shore	22.4 ± 4.6	26.0 ± 6.5	3.6	0.0023

MTMR12	5	8111339	475.0 ± 119.1	402.1 ± 100.2	-72.9	0.0002	0.013	cg02285003	Body	Open sea	64.1 ± 3.8	67.4 ± 4.0	3.3	0.0012
MTUS1	8	8149500	433.7 ± 152.1	363.5 ± 84.6	-70.2	0.0017	0.033	cg00121389	TSS200; Body	Open sea	42.4 ± 6.9	46.3 ± 9.1	3.9	0.0097
MYCBP2	13	7972069	256.8 ± 53.4	221.8 ± 44.6	-35.0	0.0017	0.033	cg11911128	Body	Open sea	69.6 ± 4.2	72.8 ± 4.1	3.2	0.0203
MYL6	12	7956211	4,630.9 ± 266.9	4,379.0 ± 339.0	-251.9	0.0002	0.013	cg01526553	Body	Open sea	51.8 ± 7.0	55.6 ± 4.1	3.8	0.0201
MYO6	6	8120783	535.0 ± 60.3	477.3 ± 69.0	-57.6	0.0002	0.013	cg13921196	Body	Open sea	18.6 ± 2.6	21.8 ± 3.9	3.3	0.0025
MYST4	10	7928491	311.9 ± 57.0	286.3 ± 58.5	-25.6	0.0034	0.049	cg07299586	3'UTR	Open sea	61.3 ± 2.3	65.8 ± 3.6	4.5	0.0003
NCK2	2	8044133	469.7 ± 108.9	428.2 ± 108.0	-41.5	0.0034	0.049	cg22765178	5'UTR	Open sea	79.0 ± 2.8	82.4 ± 2.6	3.4	0.0005
NDUFAF2	5	8105499	91.3 ± 9.6	104.9 ± 9.9	13.5	0.0005	0.017	cg17944003	Body	Open sea	58.5 ± 8.0	62.1 ± 9.2	3.6	0.0231
NENF	1	7909603	438.7 ± 64.7	480.4 ± 79.9	41.7	0.0007	0.020	cg04416454	Body	S Shelf	80.9 ± 3.7	85.2 ± 2.8	4.4	0.00004
NFIA	1	7901788	193.8 ± 30.6	143.5 ± 27.6	-50.3	0.0002	0.013	cg00560072	Body	Open sea	72.4 ± 4.5	75.8 ± 4.2	3.3	0.0094
NIN	14	7979044	147.5 ± 25.1	131.3 ± 20.4	-16.3	0.0002	0.013	cg12044531	3'UTR	Open sea	81.6 ± 5.1	85.3 ± 3.4	3.6	0.0168
NOTCH3	19	8034940	199.0 ± 32.3	173.3 ± 19.1	-25.7	0.0005	0.017	cg22809798	Body	N Shore	72.8 ± 3.4	76.2 ± 2.9	3.4	0.0022
NPAT	11	7951497	230.1 ± 29.3	204.9 ± 34.5	-25.2	0.0024	0.041	cg25630123	3'UTR	Open sea	65.3 ± 3.3	69.6 ± 4.0	4.3	0.0018
NR4A2	2	8055952	90.7 ± 17.7	127.9 ± 32.0	37.2	0.0012	0.027	cg14811105	Body	Island	57.9 ± 6.8	60.9 ± 5.7	3.1	0.0279
NR5A2	1	7908597	1,120.8 ± 459.3	772.7 ± 363.5	-348.1	0.0005	0.017	cg16046444	Body	N Shore	44.0 ± 11.7	47.8 ± 10.2	3.9	0.0397
NRIP1	21	8069553	418.2 ± 56.2	360.8 ± 63.0	-57.4	0.0002	0.013	cg07543138	5'UTR	N Shelf	62.6 ± 5.8	65.6 ± 6.3	3.0	0.0209
NTN4	12	7965573	482.7 ± 141.5	418.8 ± 134.1	-63.9	0.0002	0.013	cg25367249	Body	Open sea	58.8 ± 6.9	64.4 ± 7.0	5.6	0.0036
ODZ4	11	7950701	63.0 ± 10.1	75.6 ± 13.5	12.6	0.0002	0.013	cg07441953	Body	Open sea	80.0 ± 6.0	83.0 ± 3.3	3.0	0.0325
ODZ4	11	7950701	63.0 ± 10.1	75.6 ± 13.5	12.6	0.0002	0.013	cg26452771	5'UTR	Open sea	74.2 ± 5.5	77.5 ± 4.7	3.3	0.0147
ODZ4	11	7950701	63.0 ± 10.1	75.6 ± 13.5	12.6	0.0002	0.013	cg05340269	5'UTR	Open sea	74.5 ± 3.3	77.8 ± 4.0	3.3	0.0033
ODZ4	11	7950701	63.0 ± 10.1	75.6 ± 13.5	12.6	0.0002	0.013	cg08913922	5'UTR	Island	39.2 ± 4.7	42.7 ± 4.3	3.5	0.0270
ODZ4	11	7950701	63.0 ± 10.1	75.6 ± 13.5	12.6	0.0002	0.013	cg12830752	5'UTR	Open sea	67.4 ± 6.5	70.9 ± 5.5	3.5	0.0069
PAM	5	8107133	3,515.3 ± 1,199.5	4,440.6 ± 1,304.3	925.2	0.0002	0.013	cg20131596	TSS1500	N Shore	37.9 ± 5.9	40.9 ± 6.6	3.0	0.0485
PAPPA	9	8157487	53.5 ± 17.7	43.5 ± 7.8	-10.0	0.0002	0.013	cg13656878	Body	Open sea	40.8 ± 9.5	44.1 ± 9.8	3.2	0.0088
PBLD	10	7933982	139.3 ± 33.7	115.6 ± 40.4	-23.8	0.0017	0.033	cg06898463	5'UTR	N Shore	83.9 ± 2.2	87.2 ± 2.0	3.3	0.0002

PCK1	20	8063590	192.3 ± 120.1	333.5 ± 175.2	141.2	0.0024	0.041	cg16994880	3'UTR	Open sea	72.8 ± 11.8	79.5 ± 5.7	6.7	0.0080
PDE1A	2	8057486	42.6 ± 17.1	33.5 ± 10.5	-9.1	0.0024	0.041	cg08759112	TSS1500	Open sea	38.1 ± 12.3	42.1 ± 13.4	4.0	0.0230
PDE5A	4	8102532	216.2 ± 39.3	165.5 ± 35.8	-50.6	0.0005	0.017	cg06531595	Body	Open sea	82.0 ± 6.2	85.5 ± 4.3	3.5	0.0394
PDE5A	4	8102532	216.2 ± 39.3	165.5 ± 35.8	-50.6	0.0005	0.017	cg23138179	Body; 5'UTR	N Shore	48.6 ± 9.2	53.9 ± 9.0	5.3	0.0303
PDGFD	11	7951351	436.5 ± 182.5	263.3 ± 147.5	-173.2	0.0007	0.020	cg09194202	Body	S Shelf	82.6 ± 4.8	86.2 ± 2.7	3.6	0.0031
PDGFRA	4	8095080	199.3 ± 131.4	124.0 ± 62.3	-75.3	0.0002	0.013	cg02759489	5'UTR	Island	10.6 ± 5.1	14.5 ± 6.3	3.9	0.0036
PDK4	7	8141094	2,530.7 ± 813.9	3,706.4 ± 941.1	1,175.8	0.0002	0.013	cg03603062	Body	N Shore	64.6 ± 4.2	67.9 ± 6.0	3.3	0.0365
PDZD4	X	8175860	62.2 ± 12.4	74.9 ± 10.1	12.7	0.0002	0.013	cg08065271	Body	N Shelf	64.7 ± 5.4	68.0 ± 7.1	3.3	0.0269
PI3	20	8062927	172.4 ± 90.6	124.0 ± 50.8	-48.3	0.0024	0.041	cg09462575	TSS200	Open sea	48.7 ± 3.9	52.0 ± 4.4	3.3	0.0052
PIK3C2B	1	7923662	82.7 ± 9.4	69.6 ± 8.7	-13.1	0.0005	0.017	cg21195376	5'UTR	Open sea	52.9 ± 5.1	56.0 ± 4.4	3.2	0.0366
PIK3R1	5	8105778	380.2 ± 80.4	317.7 ± 84.4	-62.5	0.0005	0.017	cg22592475	Body	Open sea	40.6 ± 4.0	44.6 ± 5.6	4.0	0.0010
PIP5K1B	9	8155673	65.4 ± 15.8	51.8 ± 15.4	-13.6	0.0005	0.017	cg13750902	5'UTR	N Shore	70.7 ± 3.7	74.5 ± 3.4	3.8	0.0016
PLAT	8	8150509	692.9 ± 431.5	392.8 ± 163.5	-300.1	0.0002	0.013	cg24514275	Body	Open sea	64.0 ± 4.6	67.0 ± 3.7	3.0	0.0336
PLS3	X	8169473	826.5 ± 211.2	673.7 ± 174.4	-152.8	0.0002	0.013	cg11616384	Body	Open sea	49.6 ± 9.5	52.9 ± 10.1	3.3	0.0138
PLXNB2	22	8076998	508.0 ± 71.9	463.4 ± 74.3	-44.6	0.0034	0.049	cg01234546	Body	N Shore	60.8 ± 12.9	65.5 ± 12.9	4.7	0.0002
PNLIP	10	7930777	2,381.3 ± 1,549.5	1,964.5 ± 1,480.7	-416.8	0.0012	0.027	cg23728588	TSS1500	Open sea	69.3 ± 6.5	72.6 ± 4.9	3.2	0.0219
POF1B	X	8173869	208.2 ± 72.6	154.8 ± 61.1	-53.3	0.0002	0.013	cg08238865	5'UTR	Open sea	45.9 ± 4.3	48.9 ± 5.3	3.0	0.0379
POF1B	X	8173869	208.2 ± 72.6	154.8 ± 61.1	-53.3	0.0002	0.013	cg10907112	TSS200	Open sea	38.5 ± 4.7	41.7 ± 4.8	3.2	0.0038
POLD3	11	7942527	183.1 ± 19.9	157.8 ± 23.2	-25.3	0.0007	0.020	cg21048411	Body	S Shelf	70.8 ± 6.2	76.1 ± 3.3	5.3	0.0080
PPAP2A	5	8112107	167.4 ± 25.0	137.1 ± 24.8	-30.3	0.0002	0.013	cg07862423	Body	Island	61.1 ± 4.8	64.9 ± 4.3	3.9	0.0115
PRICKLE1	12	7962375	140.8 ± 42.9	105.2 ± 25.2	-35.5	0.0002	0.013	cg15852150	5'UTR	N Shore	66.1 ± 5.5	69.5 ± 6.7	3.4	0.0386
PRKDC	8	8150599	374.9 ± 61.5	322.4 ± 57.0	-52.4	0.0007	0.020	cg11773920	3'UTR	Open sea	58.7 ± 4.4	62.1 ± 5.6	3.4	0.0166
PRKDC	8	8150599	374.9 ± 61.5	322.4 ± 57.0	-52.4	0.0007	0.020	cg22338356	Body	Open sea	73.4 ± 5.7	77.2 ± 4.1	3.7	0.0024
PRKDC	8	8150599	374.9 ± 61.5	322.4 ± 57.0	-52.4	0.0007	0.020	cg04024910	Body	N Shelf	72.5 ± 3.0	77.0 ± 3.6	4.5	0.0003
PTCD3	2	8043251	542.9 ± 61.5	484.8 ± 73.8	-58.1	0.0024	0.041	cg19622777	Body	Open sea	78.0 ± 2.9	81.6 ± 2.9	3.6	0.0067

PTCD3	2	8043251	542.9 ± 61.5	484.8 ± 73.8	-58.1	0.0024	0.041	cg00350932	Body	S Shelf	42.9 ± 7.8	47.5 ± 9.8	4.5	0.0103
PTP4A1	6	8120431	1,844.6 ± 245.8	1,727.6 ± 233.7	-117.0	0.0002	0.013	cg12857652	Body	Open sea	54.3 ± 8.9	51.0 ± 8.9	-3.2	0.0455
PXDN	2	8050007	319.5 ± 149.3	235.8 ± 69.4	-83.7	0.0024	0.041	cg12760299	Body	Open sea	37.7 ± 7.9	41.1 ± 6.9	3.4	0.0276
PXDN	2	8050007	319.5 ± 149.3	235.8 ± 69.4	-83.7	0.0024	0.041	cg07029777	Body	Open sea	73.9 ± 3.7	77.8 ± 3.2	3.9	0.0014
RABGAP1L	1	7907492	196.7 ± 30.1	175.6 ± 34.2	-21.0	0.0005	0.017	cg05768419	Body	Open sea	55.8 ± 12.9	60.1 ± 11.7	4.3	0.0006
RASAL2	1	7907611	114.5 ± 20.7	101.4 ± 14.8	-13.1	0.0017	0.033	cg00112588	TSS1500	N Shore	53.7 ± 4.6	56.7 ± 3.7	3.0	0.0171
RASAL2	1	7907611	114.5 ± 20.7	101.4 ± 14.8	-13.1	0.0017	0.033	cg22685215	Body	Open sea	56.7 ± 5.4	60.0 ± 5.8	3.4	0.0245
RBCK1	20	8060353	161.7 ± 14.5	176.5 ± 17.9	14.8	0.0034	0.049	cg13812132	Body	S Shelf	75.5 ± 2.9	79.1 ± 2.7	3.7	0.0009
RBM47	4	8099967	1,112.2 ± 121.3	988.7 ± 146.7	-123.5	0.0034	0.049	cg09141635	5'UTR	Open sea	42.6 ± 6.4	45.9 ± 5.7	3.3	0.0395
RCBTB2	13	7971573	133.8 ± 38.4	114.5 ± 29.4	-19.3	0.0002	0.013	cg17300047	TSS1500	S Shore	66.0 ± 3.0	69.3 ± 1.8	3.3	0.0031
RFTN1	3	8085665	68.6 ± 11.2	61.0 ± 6.3	-7.6	0.0002	0.013	cg26792295	5'UTR	Open sea	77.5 ± 3.5	81.2 ± 2.4	3.7	0.0035
RGL1	1	7908125	274.4 ± 70.5	231.9 ± 61.1	-42.5	0.0005	0.017	cg13951632	3'UTR	Open sea	80.3 ± 4.2	83.4 ± 1.9	3.1	0.0072
RGL1	1	7908125	274.4 ± 70.5	231.9 ± 61.1	-42.5	0.0005	0.017	cg12425286	5'UTR	Open sea	76.4 ± 3.9	80.1 ± 4.2	3.7	0.0087
RGNEF	5	8106210	169.2 ± 32.0	147.9 ± 33.9	-21.3	0.0024	0.041	cg04480106	5'UTR	Open sea	58.8 ± 9.9	63.3 ± 8.1	4.5	0.0113
RGS17	6	8130394	163.9 ± 30.8	205.8 ± 51.1	41.8	0.0007	0.020	cg24028809	5'UTR	Open sea	45.7 ± 11.2	49.4 ± 11.2	3.7	0.0131
RGS7	1	7925457	131.0 ± 56.5	164.1 ± 67.7	33.1	0.0012	0.027	cg09786278	Body	Open sea	74.2 ± 5.1	77.5 ± 4.6	3.3	0.0006
RIMBP2	12	7967660	189.5 ± 72.0	224.4 ± 77.6	34.8	0.0012	0.027	cg05308244	5'UTR	Open sea	58.8 ± 22.7	62.3 ± 22.3	3.5	0.0045
RIN2	20	8061247	336.8 ± 74.2	290.2 ± 69.0	-46.6	0.0017	0.033	cg04466743	Body	Open sea	62.4 ± 4.1	65.4 ± 5.5	3.0	0.0301
RIN2	20	8061247	336.8 ± 74.2	290.2 ± 69.0	-46.6	0.0017	0.033	cg09035925	Body	Open sea	34.9 ± 5.1	38.3 ± 6.7	3.5	0.0083
RIN2	20	8061247	336.8 ± 74.2	290.2 ± 69.0	-46.6	0.0017	0.033	cg26396492	Body	Open sea	32.6 ± 4.7	36.3 ± 6.1	3.7	0.0037
RIN2	20	8061247	336.8 ± 74.2	290.2 ± 69.0	-46.6	0.0017	0.033	cg18952506	Body	Open sea	24.9 ± 7.0	28.8 ± 9.6	4.0	0.0065
ROBO1	3	8088919	306.9 ± 118.7	273.6 ± 104.6	-33.2	0.0012	0.027	cg08147563	Body	Open sea	43.5 ± 6.6	47.7 ± 5.8	4.2	0.0006
RPH3AL	17	8010903	214.8 ± 77.7	249.2 ± 102.3	34.4	0.0017	0.033	cg06562014	Body	Open sea	67.7 ± 3.5	70.8 ± 3.4	3.1	0.0059
RPH3AL	17	8010903	214.8 ± 77.7	249.2 ± 102.3	34.4	0.0017	0.033	cg12887033	Body	S Shelf	54.4 ± 7.3	57.5 ± 6.2	3.1	0.0140
RPH3AL	17	8010903	214.8 ± 77.7	249.2 ± 102.3	34.4	0.0017	0.033	cg22251117	5'UTR	N Shelf	63.6 ± 5.0	66.7 ± 4.9	3.1	0.0202

RPH3AL	17	8010903	214.8 ± 77.7	249.2 ± 102.3	34.4	0.0017	0.033	cg18771195	5'UTR	N Shelf	67.2 ± 6.5	71.0 ± 5.6	3.7	0.0217
RPL18A	2	8052022	532.4 ± 155.4	587.8 ± 122.2	55.4	0.0017	0.033	cg06508005	Body	S Shore	60.5 ± 2.8	64.4 ± 3.7	3.9	0.0008
RPL24	6	8126450	2,123.5 ± 278.9	2,342.1 ± 267.1	218.6	0.0005	0.017	cg06789412	1stExon; 5'UTR	Island	8.4 ± 4.5	11.8 ± 7.0	3.4	0.0118
RPL24	3	8089249	3,145.3 ± 364.1	3,459.5 ± 387.4	314.2	0.0002	0.013	cg06789412	1stExon; 5'UTR	Island	8.4 ± 4.5	11.8 ± 7.0	3.4	0.0118
RPL29	3	8086148	3,676.1 ± 655.2	4,022.2 ± 594.6	346.1	0.0007	0.020	cg06489668	TSS1500	S Shore	69.0 ± 3.5	72.2 ± 5.1	3.3	0.0389
RPL30	8	8151935	2,859.9 ± 228.0	3,084.9 ± 256.3	224.9	0.0007	0.020	cg12992040	Body	N Shelf	74.1 ± 5.3	77.2 ± 4.9	3.1	0.0160
RPL39	6	8127526	1,159.5 ± 234.8	1,305.8 ± 258.0	146.3	0.0002	0.013	cg22865905	Body	N Shelf	53.5 ± 5.7	56.8 ± 5.8	3.3	0.0087
RPL39	X	8174710	1,108.4 ± 221.3	1,245.2 ± 237.3	136.8	0.0005	0.017	cg22865905	Body	N Shelf	53.5 ± 5.7	56.8 ± 5.8	3.3	0.0087
RPL41	12	7956159	112.1 ± 18.0	137.9 ± 23.4	25.8	0.0002	0.013	cg25161889	TSS1500	N Shelf	41.1 ± 4.3	44.3 ± 6.7	3.2	0.0412
RPL41	20	8061364	5,182.7 ± 350.5	5,436.7 ± 427.7	254.0	0.0005	0.017	cg25161889	TSS1500	N Shelf	41.1 ± 4.3	44.3 ± 6.7	3.2	0.0412
RPL41	22	8075691	5,435.0 ± 336.7	5,678.5 ± 445.2	243.6	0.0002	0.013	cg25161889	TSS1500	N Shelf	41.1 ± 4.3	44.3 ± 6.7	3.2	0.0412
RPL41	5	8105432	5,306.0 ± 323.5	5,536.2 ± 433.4	230.2	0.0005	0.017	cg25161889	TSS1500	N Shelf	41.1 ± 4.3	44.3 ± 6.7	3.2	0.0412
RPS18	6	8118644	1,928.1 ± 361.3	2,116.1 ± 382.8	188.0	0.0034	0.049	cg12086028	Body	S Shore	52.4 ± 3.9	55.9 ± 5.3	3.6	0.0118
RPS18	6	8118644	1,928.1 ± 361.3	2,116.1 ± 382.8	188.0	0.0034	0.049	cg05560494	Body	S Shore	65.2 ± 3.6	69.1 ± 3.0	3.9	0.0018
RPS18	6	8118644	1,928.1 ± 361.3	2,116.1 ± 382.8	188.0	0.0034	0.049	cg24664526	TSS1500	Island	8.6 ± 3.5	12.7 ± 6.9	4.1	0.0023
RPS18	6_cox_hap1	8178253	1,928.1 ± 361.3	2,116.1 ± 382.8	188.0	0.0034	0.049	cg12086028	Body	S Shore	52.4 ± 3.9	55.9 ± 5.3	3.6	0.0118
RPS18	6_cox_hap1	8178253	1,928.1 ± 361.3	2,116.1 ± 382.8	188.0	0.0034	0.049	cg05560494	Body	S Shore	65.2 ± 3.6	69.1 ± 3.0	3.9	0.0018
RPS18	6_cox_hap1	8178253	1,928.1 ± 361.3	2,116.1 ± 382.8	188.0	0.0034	0.049	cg24664526	TSS1500	Island	8.6 ± 3.5	12.7 ± 6.9	4.1	0.0023
RPS18	6_qbl_hap2	8179544	1,928.1 ± 361.3	2,116.1 ± 382.8	188.0	0.0034	0.049	cg12086028	Body	S Shore	52.4 ± 3.9	55.9 ± 5.3	3.6	0.0118
RPS18	6_qbl_hap2	8179544	1,928.1 ± 361.3	2,116.1 ± 382.8	188.0	0.0034	0.049	cg05560494	Body	S Shore	65.2 ± 3.6	69.1 ± 3.0	3.9	0.0018
RPS18	6_qbl_hap2	8179544	1,928.1 ± 361.3	2,116.1 ± 382.8	188.0	0.0034	0.049	cg24664526	TSS1500	Island	8.6 ± 3.5	12.7 ± 6.9	4.1	0.0023
RTKN	2	8053142	141.7 ± 16.0	157.6 ± 17.9	15.9	0.0002	0.013	cg22442732	Body	N Shelf	67.6 ± 5.9	71.2 ± 4.2	3.7	0.0249
SAMD12	8	8152506	109.6 ± 16.5	90.3 ± 15.8	-19.3	0.0017	0.033	cg22533992	Body	Open sea	70.4 ± 4.0	73.6 ± 6.0	3.2	0.0235
SCD5	4	8101366	205.9 ± 57.3	179.7 ± 58.0	-26.3	0.0005	0.017	cg09031823	Body	Open sea	56.5 ± 7.9	59.9 ± 8.1	3.4	0.0087
SEC24D	4	8102482	598.9 ± 145.2	726.1 ± 161.3	127.3	0.0005	0.017	cg04428346	Body	Open sea	57.4 ± 6.6	61.8 ± 7.2	4.4	0.0003

SEL1L	14	7980547	1,149.9 ± 154.0	1,217.1 ± 162.3	67.2	0.0034	0.049	cg23295683	Body	N Shore	52.3 ± 5.6	56.0 ± 5.2	3.7	0.0094
SEMA6A	5	8113666	202.4 ± 59.3	158.2 ± 59.7	-44.3	0.0002	0.013	cg06583577	5'UTR	Open sea	70.2 ± 5.9	73.9 ± 4.4	3.7	0.0128
SGSM1	22	8071953	86.7 ± 24.7	97.1 ± 22.2	10.3	0.0017	0.033	cg03942471	Body	S Shelf	73.0 ± 2.7	76.2 ± 2.4	3.2	0.0028
SH3BP5	3	8085556	84.6 ± 11.5	103.3 ± 17.0	18.7	0.0002	0.013	cg05046996	TSS200	Open sea	51.8 ± 5.3	54.8 ± 3.8	3.0	0.0152
SH3BP5	3	8085556	84.6 ± 11.5	103.3 ± 17.0	18.7	0.0002	0.013	cg18444702	Body; 5'UTR	Open sea	55.0 ± 6.0	58.2 ± 6.3	3.2	0.0235
SH3BP5	3	8085556	84.6 ± 11.5	103.3 ± 17.0	18.7	0.0002	0.013	cg03495084	Body	Open sea	67.4 ± 6.2	71.7 ± 8.1	4.3	0.0409
SHCBP1	16	8001133	71.5 ± 38.5	38.1 ± 8.6	-33.4	0.0002	0.013	cg04112058	Body	Open sea	57.0 ± 6.4	60.0 ± 6.2	3.0	0.0302
SHMT2	12	7956401	548.7 ± 125.7	643.2 ± 157.6	94.5	0.0005	0.017	cg26220528	TSS1500; TSS200	N Shore	5.6 ± 4.0	9.3 ± 7.6	3.7	0.0009
SIPA1L1	14	7975459	282.5 ± 31.1	257.3 ± 33.9	-25.2	0.0012	0.027	cg02058870	5'UTR	Open sea	34.2 ± 13.2	37.4 ± 13.1	3.2	0.0249
SIPA1L1	14	7975459	282.5 ± 31.1	257.3 ± 33.9	-25.2	0.0012	0.027	cg10727432	Body	Open sea	64.6 ± 5.4	68.1 ± 5.8	3.5	0.0027
SLC16A7	12	7956658	411.4 ± 151.2	308.9 ± 128.7	-102.6	0.0002	0.013	cg26090940	Body	Open sea	64.1 ± 6.1	67.3 ± 7.1	3.2	0.0052
SLC24A6	12	7966600	106.2 ± 23.8	94.2 ± 23.8	-11.9	0.0005	0.017	cg22510037	5'UTR	N Shore	28.9 ± 8.5	32.1 ± 8.3	3.1	0.0248
SLC38A9	5	8112121	246.5 ± 39.5	201.6 ± 42.6	-44.9	0.0005	0.017	cg07042489	Body	Open sea	79.4 ± 3.3	82.4 ± 3.2	3.0	0.0061
SLC7A7	14	7977786	67.1 ± 20.4	53.4 ± 12.5	-13.6	0.0007	0.020	cg12775909	TSS1500; 5'UTR	Open sea	78.3 ± 5.4	81.7 ± 3.8	3.4	0.0073
SLC7A7	14	7977786	67.1 ± 20.4	53.4 ± 12.5	-13.6	0.0007	0.020	cg20495370	Body	Open sea	52.1 ± 6.2	55.6 ± 6.4	3.5	0.0469
SMAD3	15	7984364	364.4 ± 161.7	326.4 ± 190.1	-38.0	0.0024	0.041	cg15799109	Body; 5'UTR	Open sea	75.6 ± 5.8	78.7 ± 5.7	3.1	0.0487
SOCS5	2	8041820	335.0 ± 40.0	300.9 ± 46.7	-34.1	0.0017	0.033	cg12224879	5'UTR	Open sea	57.2 ± 5.6	60.4 ± 3.8	3.2	0.0088
SPRED1	15	7982564	238.0 ± 47.0	208.6 ± 32.5	-29.4	0.0024	0.041	cg03028506	Body	Open sea	69.2 ± 4.6	72.6 ± 4.5	3.4	0.0089
SPTLC3	20	8060997	102.3 ± 25.6	82.1 ± 20.3	-20.2	0.0024	0.041	cg11745355	Body	Open sea	61.2 ± 3.6	64.6 ± 2.3	3.4	0.0053
SRP9	1	7910099	1,084.0 ± 97.6	1,000.7 ± 113.8	-83.3	0.0034	0.049	cg00311360	Body; 3'UTR	Open sea	79.0 ± 3.9	82.1 ± 3.4	3.1	0.0054
SRP9	1	7910099	1,084.0 ± 97.6	1,000.7 ± 113.8	-83.3	0.0034	0.049	cg26298737	TSS1500	N Shore	35.5 ± 6.7	38.8 ± 7.1	3.2	0.0049
SRP9	10	7935002	1,605.0 ± 135.9	1,468.0 ± 172.7	-137.0	0.0012	0.027	cg00311360	Body; 3'UTR	Open sea	79.0 ± 3.9	82.1 ± 3.4	3.1	0.0054
SRP9	10	7935002	1,605.0 ± 135.9	1,468.0 ± 172.7	-137.0	0.0012	0.027	cg26298737	TSS1500	N Shore	35.5 ± 6.7	38.8 ± 7.1	3.2	0.0049
ST7	7	8135638	244.6 ± 36.1	271.4 ± 41.6	26.8	0.0024	0.041	cg00764217	Body	Open sea	25.0 ± 8.1	28.2 ± 8.9	3.2	0.0176

ST7	7	8135638	244.6 ± 36.1	271.4 ± 41.6	26.8	0.0024	0.041	cg24895142	Body	Open sea	33.4 ± 11.0	37.1 ± 10.2	3.7	0.0395
STAT5B	17	8015590	263.3 ± 25.1	305.8 ± 40.2	42.5	0.0017	0.033	cg07402310	5'UTR	N Shelf	73.9 ± 4.6	77.1 ± 3.4	3.3	0.0091
STK17B	2	8057887	286.7 ± 59.3	244.7 ± 55.9	-42.1	0.0007	0.020	cg15023571	5'UTR	N Shore	25.8 ± 4.8	28.8 ± 5.5	3.0	0.0099
STMN1	1	7913869	293.6 ± 31.6	251.5 ± 55.8	-42.2	0.0005	0.017	cg09518245	TSS1500	S Shore	69.7 ± 3.6	74.0 ± 3.4	4.3	0.0019
TANC1	2	8045889	129.1 ± 27.8	112.5 ± 20.9	-16.6	0.0024	0.041	cg25348841	5'UTR	Open sea	78.4 ± 3.1	81.4 ± 4.0	3.0	0.0204
TANC1	2	8045889	129.1 ± 27.8	112.5 ± 20.9	-16.6	0.0024	0.041	cg21216100	Body	Open sea	77.6 ± 5.9	82.1 ± 3.7	4.6	0.0028
TAOK3	12	7966851	317.4 ± 35.0	274.6 ± 34.7	-42.8	0.0007	0.020	cg20353780	3'UTR	Open sea	58.9 ± 6.2	62.6 ± 6.2	3.7	0.0054
TBC1D1	4	8094574	269.5 ± 31.2	230.7 ± 20.1	-38.8	0.0002	0.013	cg09569989	Body	Open sea	65.9 ± 5.6	69.2 ± 6.4	3.2	0.0254
TBC1D1	4	8094574	269.5 ± 31.2	230.7 ± 20.1	-38.8	0.0002	0.013	cg19090437	Body	Open sea	57.2 ± 4.3	60.8 ± 5.8	3.6	0.0120
TBC1D1	4	8094574	269.5 ± 31.2	230.7 ± 20.1	-38.8	0.0002	0.013	cg10278506	Body	Open sea	74.9 ± 3.6	78.7 ± 3.4	3.8	0.0011
TBC1D1	4	8094574	269.5 ± 31.2	230.7 ± 20.1	-38.8	0.0002	0.013	cg17508905	Body	Open sea	71.0 ± 3.5	75.2 ± 3.6	4.2	0.0006
TBXAS1	7	8136557	117.7 ± 33.2	95.1 ± 23.8	-22.5	0.0007	0.020	cg07590004	5'UTR	Open sea	81.6 ± 3.8	84.9 ± 2.3	3.3	0.0002
TCF12	15	7983843	512.5 ± 59.2	460.3 ± 51.2	-52.2	0.0024	0.041	cg26810157	3'UTR	Open sea	54.4 ± 12.4	58.1 ± 12.9	3.7	0.0460
TCF7L2	10	7930537	215.1 ± 30.9	180.2 ± 28.9	-34.9	0.0002	0.013	cg26775558	Body	Open sea	56.5 ± 10.0	60.4 ± 9.2	3.9	0.0060
TFDP2	3	8091103	319.3 ± 39.2	272.5 ± 46.1	-46.9	0.0002	0.013	cg21593986	TSS1500	Open sea	79.8 ± 3.0	83.2 ± 2.4	3.4	0.0022
TGFBR2	3	8078350	267.5 ± 55.9	234.6 ± 59.9	-32.9	0.0034	0.049	cg21814995	Body	Open sea	43.5 ± 9.7	46.8 ± 8.6	3.3	0.0055
TMC5	16	7993638	512.3 ± 193.4	388.7 ± 182.5	-123.5	0.0002	0.013	cg07998072	5'UTR	S Shelf	77.1 ± 3.5	80.5 ± 3.1	3.4	0.0091
TMC7	16	7993588	76.8 ± 22.1	68.4 ± 19.1	-8.4	0.0024	0.041	cg06428742	TSS1500	N Shore	66.3 ± 4.6	69.7 ± 4.5	3.4	0.0152
TMEM2	9	8161701	489.1 ± 123.3	441.4 ± 109.9	-47.8	0.0034	0.049	cg14565465	3'UTR	Open sea	54.0 ± 9.2	57.2 ± 8.4	3.3	0.0433
TMEM200A	6	8122038	113.3 ± 61.9	148.8 ± 59.3	35.5	0.0012	0.027	cg26931050	TSS1500	Open sea	78.2 ± 4.8	82.1 ± 3.6	3.9	0.0048
TMEM63B	6	8119926	168.6 ± 21.2	149.1 ± 19.3	-19.4	0.0034	0.049	cg00507055	Body	N Shore	83.8 ± 4.6	86.9 ± 2.8	3.1	0.0065
TMEM9B	11	7946439	627.4 ± 76.8	557.3 ± 68.0	-70.1	0.0002	0.013	cg16733419	Body	N Shelf	49.2 ± 4.0	52.7 ± 4.0	3.4	0.0057
TNFSF12-TNFSF13	17	8004464	110.5 ± 11.6	121.9 ± 13.5	11.4	0.0024	0.041	cg08090557	Body	N Shore	49.0 ± 3.9	52.1 ± 5.3	3.1	0.0050
TRAM2	6	8127051	124.4 ± 25.5	105.7 ± 18.9	-18.8	0.0017	0.033	cg12793314	Body	Open sea	82.9 ± 2.5	86.1 ± 2.8	3.1	0.0020
TRIM22	11	7938035	161.8 ± 53.4	111.8 ± 34.1	-49.9	0.0005	0.017	cg17025642	TSS1500	Open sea	78.6 ± 6.0	82.1 ± 2.8	3.5	0.0271

TSPAN4	11	7937518	108.7 ± 14.1	117.3 ± 12.8	8.6	0.0017	0.033	cg15164881	5'UTR; Body	Island	66.9 ± 5.4	71.4 ± 4.1	4.6	0.0105
TSPYL1	6	8129099	1,368.2 ± 169.9	1,268.3 ± 147.7	-100.0	0.0024	0.041	cg19341652	1stExon	N Shore	17.8 ± 3.1	20.9 ± 3.0	3.1	0.0021
TSPYL2	X	8167763	187.6 ± 55.3	254.8 ± 62.1	67.2	0.0005	0.017	cg22170936	TSS200	Island	28.8 ± 19.5	25.7 ± 16.6	-3.1	0.0364
TUBA1C	12	7955179	475.0 ± 129.8	538.5 ± 133.4	63.5	0.0034	0.049	cg19426128	3'UTR	Open sea	78.9 ± 4.9	83.4 ± 2.9	4.4	0.0049
TUBB2B	6	8123651	246.5 ± 63.7	292.8 ± 80.0	46.3	0.0007	0.020	cg24138650	3'UTR	Island	76.3 ± 2.2	79.4 ± 1.4	3.2	0.0013
UACA	15	7990054	122.9 ± 27.6	94.2 ± 21.1	-28.7	0.0007	0.020	cg10177766	Body	Open sea	78.0 ± 5.1	81.4 ± 4.9	3.4	0.0326
UBD	6	8124650	364.3 ± 192.3	260.4 ± 144.7	-103.9	0.0005	0.017	cg14809932	Body	S Shelf	63.1 ± 4.4	66.4 ± 5.5	3.3	0.0108
UBD	6_cox_hap1	8178295	345.5 ± 178.3	246.1 ± 137.0	-99.4	0.0002	0.013	cg14809932	Body	S Shelf	63.1 ± 4.4	66.4 ± 5.5	3.3	0.0108
UBR2	6	8119529	493.6 ± 68.7	452.6 ± 73.5	-41.0	0.0005	0.017	cg20646500	Body	S Shelf	40.4 ± 9.3	45.2 ± 7.1	4.9	0.0111
UTRN	6	8122464	201.0 ± 30.7	177.9 ± 34.7	-23.1	0.0024	0.041	cg22560545	TSS200	Open sea	62.4 ± 6.5	65.4 ± 7.7	3.0	0.0352
UTRN	6	8122464	201.0 ± 30.7	177.9 ± 34.7	-23.1	0.0024	0.041	cg26926221	Body	Open sea	39.3 ± 4.6	42.4 ± 3.8	3.1	0.0147
UTRN	6	8122464	201.0 ± 30.7	177.9 ± 34.7	-23.1	0.0024	0.041	cg01524618	Body	Open sea	40.4 ± 7.0	43.6 ± 7.0	3.1	0.0178
UTRN	6	8122464	201.0 ± 30.7	177.9 ± 34.7	-23.1	0.0024	0.041	cg13800496	Body	Open sea	36.0 ± 4.0	39.3 ± 3.8	3.3	0.0036
UXT	X	8172358	421.2 ± 36.9	484.0 ± 32.8	62.7	0.0012	0.027	cg03689540	Body	N Shore	62.7 ± 6.7	67.1 ± 4.4	4.4	0.0119
WDR44	X	8169519	208.8 ± 38.1	195.2 ± 41.1	-13.6	0.0034	0.049	cg13674559	TSS1500	Island	17.2 ± 12.3	20.5 ± 12.2	3.3	0.0037
VPS13D	1	7897890	181.6 ± 28.9	164.4 ± 24.3	-17.2	0.0034	0.049	cg10204259	Body	Open sea	65.1 ± 6.7	68.4 ± 6.0	3.4	0.0145
VPS13D	1	7897890	181.6 ± 28.9	164.4 ± 24.3	-17.2	0.0034	0.049	cg10798815	3'UTR	Open sea	70.3 ± 6.2	74.9 ± 4.8	4.6	0.0152
VPS41	7	8139165	1,600.3 ± 234.3	1,469.6 ± 270.6	-130.8	0.0012	0.027	cg04957903	Body	Open sea	70.5 ± 5.0	74.4 ± 6.1	4.0	0.0036
XRCC5	2	8048146	2,279.6 ± 144.3	2,112.4 ± 182.8	-167.2	0.0017	0.033	cg07011981	Body	Open sea	73.0 ± 5.0	76.4 ± 2.7	3.4	0.0095
ZBTB38	3	8083090	1,315.7 ± 219.0	1,174.0 ± 232.6	-141.7	0.0012	0.027	cg21612382	5'UTR	Open sea	71.7 ± 4.8	75.3 ± 3.6	3.6	0.0184
ZC3HAV1	7	8143279	442.1 ± 55.0	411.4 ± 58.0	-30.7	0.0012	0.027	cg23424043	TSS1500	S Shore	61.7 ± 4.7	65.3 ± 5.6	3.6	0.0031
ZC3HAV1	7	8143279	442.1 ± 55.0	411.4 ± 58.0	-30.7	0.0012	0.027	cg08222662	TSS1500	S Shore	12.8 ± 7.7	16.9 ± 8.1	4.1	0.0244
ZC3HAV1	7	8143279	442.1 ± 55.0	411.4 ± 58.0	-30.7	0.0012	0.027	cg00303210	Body; 3'UTR	Open sea	50.0 ± 8.4	54.4 ± 11.4	4.4	0.0172
ZNF395	8	8149986	278.1 ± 80.6	387.2 ± 146.9	109.1	0.0007	0.020	cg21330896	3'UTR	Open sea	55.4 ± 11.8	59.3 ± 10.3	4.0	0.0231
ZNF436	1	7913582	157.2 ± 23.1	131.9 ± 17.8	-25.4	0.0002	0.013	cg07106501	Body	N Shelf	54.8 ± 5.1	57.9 ± 3.9	3.0	0.0209

ZNF552	19	8039687	62.7 ± 10.5	51.6 ± 10.5	-11.1	0.0007	0.020	cg14727106	3'UTR	Open sea	68.4 ± 3.9	71.9 ± 3.9	3.5	0.0075
