# Supplementary material for: Effects of palmitate on genome-wide mRNA expression and DNA methylation patterns in human pancreatic islets
Source: BMC Med. 2014 Jun 23;12:103. doi: 10.1186/1741-7015-12-103 (PMC4065864; doi:10.1186/1741-7015-12-103)

**Supplementary Figure 1:** Results from KEGG pathway analysis using genes that exhibit differential DNA methylation (*P* <0.05) after palmitate treatment in human pancreatic islets. Includes only pathways which also displayed enrichment for genes with differential expression after palmitate treatment in human pancreatic islets.

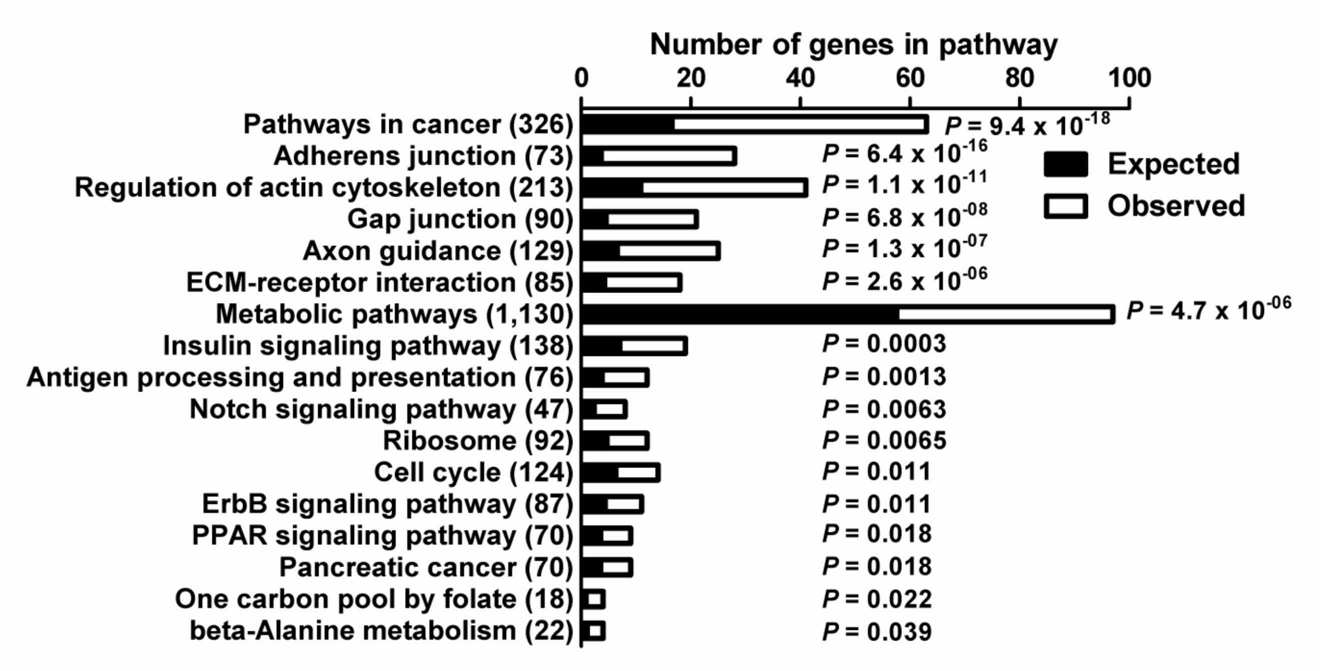

Supplement: Additional file 12: Figure S1 — Results from KEGG pathway analysis using genes that exhibit differential DNA methylation (P <0.05) in human pancreatic islets exposed to palmitate compared to control treatment. It includes only pathways which also displayed enrichment for genes with differential expression after palmitate treatment in human pancreatic islets. [file 1741-7015-12-103-S12.pdf]
