# Supplementary material for: Effects of palmitate on genome-wide mRNA expression and DNA methylation patterns in human pancreatic islets
Source: BMC Med. 2014 Jun 23;12:103. doi: 10.1186/1741-7015-12-103 (PMC4065864; doi:10.1186/1741-7015-12-103)
Supplement: Additional file 13: Table S12 — Biological pathways including genes that exhibit differential DNA methylation (P <0.05) in human pancreatic islets exposed to palmitate compared to control teatment. It includes only pathways which also displayed enrichment for genes with differential expression after palmitate treatment in human pancreatic islets. [file 1741-7015-12-103-S13.pdf]

Supplementary Table 12: Biological pathways including genes that exhibit differential DNA methylation (P <0.05) after palmitate treatment in human pancreatic islets. Includes only pathways which also displayed enrichment for genes with differential expression after palmitate treatment in human pancreatic islets.

**Pathway (total number of genes in the pathway)**	**Observed number of genes**	**Expected number of genes**	**Ratio Observed/ expected**	**Raw *P*-value**	**Adjusted *P*-value**
Pathways in cancer (326)	63	16.61	3.79	$5.09x10^{-20}$	$9.37x10^{-18}$
Adherens junction (73)	28	3.72	7.53	$6.96x10^{-18}$	$6.40x10^{-16}$
Regulation of actin cytoskeleton (213)	41	10.85	3.78	$1.77x10^{-13}$	$1.09x10^{-11}$
Gap junction (90)	21	4.58	4.58	$3.67x10^{-09}$	$6.75x10^{-08}$
Axon guidance (129)	25	6.57	3.80	$7.55x10^{-09}$	$1.26x10^{-07}$
ECM-receptor interaction (85)	18	4.33	4.16	$2.28x10^{-07}$	$2.62x10^{-06}$
Metabolic pathways (1,130)	97	57.57	1.69	$4.36x10^{-07}$	$4.72x10^{-06}$
Insulin signaling pathway (138)	19	7.03	2.70	$7.74x10^{-05}$	0.0003
Antigen processing and presentation (76)	12	3.87	3.10	0.0004	0.0013
Notch signaling pathway (47)	8	2.39	3.34	0.0024	0.0063
Ribosome (92)	12	4.69	2.56	0.0025	0.0065
Cell cycle (124)	14	6.32	2.22	0.0044	0.0105
ErbB signaling pathway (87)	11	4.43	2.48	0.0046	0.0107
PPAR signaling pathway (70)	9	3.57	2.52	0.0090	0.0180
Pancreatic cancer (70)	9	3.57	2.52	0.0090	0.0180
One carbon pool by folate (18)	4	0.92	4.36	0.0116	0.0222
beta-Alanine metabolism (22)	4	1.12	3.57	0.0235	0.0393

**Pathway (total number of genes in the pathway)**	**Observed genes**
Pathways in cancer	*CDKN2B, GLI2, FN1, COL4A2, FOXO1, FGF13, EPAS1, CTNNA3, CTNNA1, ITGA6, RAC1, CTBP2, PRKCA, PDGFRA, TGFBR2, ETS1, GSTP1, FGF19, FZD6, CTNNA2, MECOM, DAPK2, CREBBP, RB1, CDC42, IGF1R, CTNNB1, PTEN, PIK3R2, CDKN1B, BCL2, COL4A1, ARNT2, MET, CASP8, RHOA, TPM3, LAMC1, DCC, PIK3R1, FGF12, MMP1, LAMA2, MAP2K2, EGFR, FZD7, TCF7L2, RXRB, GRB2, FGF1, PIAS3, TCF7L1, JAK1, WNT5B, MSH3, PML, WNT3, RUNX1, FZD5, TRAF5, ARNT, CTBP1, EGLN1*
Adherens junction	*LMO7, WASF2, SORBS1, RHOA, CTNNA3, CTNNA1, SNAI1, RAC1, NLK, CSNK2A1, TJP1, TGFBR2, EGFR, CTNNA2, TCF7L2, CREBBP, PTPRF,*

	MLLT4, TCF7L1, FYN, PTPRJ, CDC42, PTPRB, CTNNB1, IGF1R, MET, VCL, PARD3
Regulation of actin cytoskeleton	*WASF2, SSH2, PPP1R12A, FN1, FGF13, ITGA6, RAC1, MYH9, PDGFRA, MYH10, FGF19, PIP5K1C, IQGAP2, CDC42, PIK3R2, RDX, MYH14, ITGA9, VCL, CHRM3, LIMK2, CYFIP1, PDGFC, RHOA, PIK3R1, FGF12, MYL10, ARPC1A, MAP2K2, EGFR, ARPC3, FGF1, GNA12, ARHGEF6, CHRM5, ITGAD, PDGFD, GNA13, CHRM2, PIP5K1B, FGD1*
Gap junction	*PDGFC, HTR2C, TJP1, TUBA1C, PRKCA, PDGFRA, MAP2K2, CDK1, EGFR, GRB2, LPAR1, ADCY5, ADCY2, ITPR1, ADRB1, PRKG1, PDGFD, TUBB2B, TUBB8, CSNK1D, ADCY7*
Axon guidance	*UNC5C, PPP3CA, LRRC4C, RHOA, DCC, RAC1, PLXNA2, EFNA3, ROBO1, SLIT3, SEMA6D, SEMA3E, PLXNB2, SEMA6A, CDK5, ABLIM1, FYN, CDC42, SEMA4A, NCK2, NTN4, MET, NTN1, LIMK2, UNC5D*
ECM-receptor interaction	*SV2B, FN1, COL4A2, LAMC1, COL11A2, ITGA6, TNXB, SDC2, RELN, LAMA2, CD36, COL3A1, GP5, VWF, GP6, COL4A1, CD44, ITGA9*
Metabolic pathways	*ARG2, PPT2, NDUFA5, ACACB, PCYT1B, AASS, LPCAT2, POLR1A, GALNT12, GALNT6, AGPAT1, PNLIPRP1, IDH2, TPO, EXT1, ST6GALNAC5, PIGL, CDS1, NDUFB3, FLAD1, ITPK1, ST3GAL4, GALNT9, PGAM4, DUT, ALDH1A3, GOT1, NDUFV3, HIBCH, GALNTL4, MGLL, MTHFD2L, ALLC, SEPHS1, TAT, GALNT5, SAT1, MTHFD1L, GALK2, HPSE2, PNLIPRP3, TPK1, AGPAT6, HLCS, AKR1D1, INPP4B, UQCRH, COX7B2, ITPKB, PIGN, PLCD3, PIP5K1C, C1GALT1, SMPD3, AMY2B, INPP5A, QDPR, FUT8, ALPL, CTPS2, PCK1, SHMT2, UGT1A8, STT3B, SYNJ2, GMDS, DBT, NOS3, HMGCR, NT5E, SMS, SUCLA2, GFPT1, GUK1, AK5, PRIM2, BPGM, GALC, UPB1, TRIT1, TBXAS1, C1GALT1C1, POLD3, SPTLC2, LIPC, GK2, PFKM, GCNT4, IDS, GAD2, ACSS3, GALNT2, PC, NME7, POLR1C, SPTLC3, PIP5K1B*
Insulin signaling pathway	*ACACB, SORBS1, RHOQ, RAPGEF1, FOXO1, INPP5D, PIK3R1, PRKAR1B, MAP2K2, PDE3A, GRB2, PTPRF, IRS2, RPTOR, PIK3R2, PRKCZ, PCK1, RPS6, MKNK2*
Antigen processing and presentation	*HSPA1L, IFNG, HLA-DPA1, CD8A, HLA-DQA2, HLA-DQA1, HLA-E, HLA-DMB, TAP2, HLA-C, HLA-DOB, HLA-DRB5*
Notch signaling pathway	*CREBBP, NCOR2, NOTCH4, CTBP2, MAML2, CTBP1, NCSTN, NUMB*
Ribosome	*RPL32, RPL17, RPL39, RPL14, RPS6, RPL30, RPL18A, RPL4, RPL24, RPS18, MRPL13, RPL23A*
Cell cycle	*CDKN2B, CREBBP, ANAPC5, RB1, CDC14B, PRKDC, TFDP1, MAD1L1, ATM, CDKN1B, YWHAQ, YWHAZ, CDK1, RAD21*

ErbB signaling pathway	*GRB2, ABL2, NRG3, PIK3R2, PIK3R1, NCK2, CDKN1B, PRKCA, ERBB4, MAP2K2, EGFR*
PPAR signaling pathway	*SORBS1, PCK1, FABP2, MMP1, APOA5, CD36, GK2, PLTP, RXRB*
Pancreatic cancer	*RB1, CDC42, JAK1, ARHGEF6, RAC1, PIK3R2, PIK3R1, TGFBR2, EGFR*
One carbon pool by folate	*SHMT2, MTHFD1L, MTHFD2L, ALDH1L1*
beta-Alanine metabolism	*GAD2, HIBCH, UPB1, SMS*
