# Supplementary material for: Effects of palmitate on genome-wide mRNA expression and DNA methylation patterns in human pancreatic islets
Source: BMC Med. 2014 Jun 23;12:103. doi: 10.1186/1741-7015-12-103 (PMC4065864; doi:10.1186/1741-7015-12-103)
Supplement: Additional file 14: Table S13 — Infinium HumanMethylation450K BeadChip array probes with possible cross reactivity to other locations in the genome. [file 1741-7015-12-103-S14.pdf]

Supplementary Table 13: Infinium HumanMethylation450K BeadChip array probes with possible cross reactivity to other locations in the genome.

**Probe ID**	**Numbers of cross reactive targets with matching base pairs:**			
	**47 bp**	**48 bp**	**49 bp**	**50 bp**
cg02738374	1	0	0	0
cg04031454	24	1	0	0
cg05903046	2	0	0	0
cg07106501	223	6	0	0
cg08036804	546	59	1	0
cg10278506	6	0	0	0
cg11773920	0	0	1	0
cg12224879	0	1	0	0
cg12992040	1	0	0	0
cg14727106	132	12	0	0
cg20284982	39	3	0	0
cg21577639	19	2	0	0
cg24002907	68	24	7	0
