# Supplementary material for: Effects of palmitate on genome-wide mRNA expression and DNA methylation patterns in human pancreatic islets
Source: BMC Med. 2014 Jun 23;12:103. doi: 10.1186/1741-7015-12-103 (PMC4065864; doi:10.1186/1741-7015-12-103)
Supplement: Additional file 15: Table S14 — Genes displaying significant difference in mRNA expression between control and palmitate-treated human pancreatic islets (q <0.05) as well as significant correlation between BMI and mRNA expression in human pancreatic islets (P <0.05). [file 1741-7015-12-103-S15.pdf]

Supplementary Table 14: Genes displaying significant difference in mRNA expression between control and palmitate treated human pancreatic islets (q <0.05) as well as significant correlation between BMI and mRNA expression in human pancreatic islets (P <0.05).

Gene symbol	Probe set ID	mRNA expression						BMI correlated with mRNA expression	
		Chr	Control mean ± sd	Palmitate mean ± sd	Diff. in expr. (palmitate-control)	*P*-value	*q*-value	Regression coefficient	*P*-value
ADM	7938390	11	306.1 ± 136.2	592.8 ± 468.5	286.7	0.0012	0.027	0.030	0.033
AIFM2	7934101	10	117.8 ± 19.1	134.9 ± 21.9	17.2	0.0024	0.041	0.014	0.038
ANGPTL4	8025402	19	176.2 ± 109.0	270.4 ± 181.2	94.2	0.0012	0.027	0.032	0.032
AP1M2	8034084	19	386.9 ± 61.7	357.2 ± 53.2	-29.7	0.0012	0.027	-0.016	0.016
ASPM	7923086	1	19.9 ± 5.0	14.4 ± 2.7	-5.5	0.0007	0.020	-0.021	0.040
BET1L	7945349	11	156.7 ± 15.4	182.5 ± 19.6	25.8	0.0002	0.013	0.014	0.023
BRI3BP	7959777	12	247.9 ± 34.0	222.2 ± 37.3	-25.7	0.0034	0.049	-0.016	0.046
CAMK2N1	7913237	1	1,134.0 ± 260.3	1,030.1 ± 237.0	-103.9	0.0002	0.013	-0.015	0.049
CDKN1A	8119088	6	241.8 ± 56.2	309.6 ± 72.3	67.9	0.0005	0.017	0.018	0.028
CNTN3	8088866	3	50.3 ± 10.1	40.9 ± 12.6	-9.4	0.0002	0.013	-0.022	0.046
CNTN4	8077323	3	251.9 ± 147.1	207.3 ± 117.7	-44.6	0.0024	0.041	-0.048	0.010
CRYAB	7951662	11	298.2 ± 156.6	420.3 ± 237.5	122.2	0.0034	0.049	0.052	0.006
EFR3A	8148333	8	1,179.9 ± 218.9	1,071.9 ± 203.8	-108.0	0.0005	0.017	-0.018	0.049
EIF3D	8075798	22	1,277.8 ± 227.2	1,405.8 ± 261.5	128.0	0.0012	0.027	0.015	0.009
EMP3	8030007	19	132.3 ± 41.5	159.3 ± 46.0	27.0	0.0007	0.020	0.021	0.047
EXOSC9	8097128	4	264.0 ± 19.9	286.1 ± 32.4	22.1	0.0024	0.041	0.020	0.013
FUT2	8030094	19	107.3 ± 33.9	92.5 ± 29.0	-14.8	0.0024	0.041	-0.046	0.007
FXYD3	8027748	19	426.5 ± 97.5	338.7 ± 97.4	-87.7	0.0002	0.013	-0.039	0.002
GNL3	8080419	3	757.3 ± 102.2	888.3 ± 116.3	131.1	0.0012	0.027	0.016	0.012
HIBADH	8138776	7	578.4 ± 78.2	507.3 ± 88.2	-71.1	0.0002	0.013	-0.019	0.013
HSPA1A	8118310	6	2,833.9 ± 557.5	3,360.6 ± 656.2	526.7	0.0005	0.017	0.026	0.041
HSPA1A	8179322	6_qbl_hap2	2,768.4 ± 550.8	3,294.7 ± 646.9	526.4	0.0005	0.017	0.025	0.043

HSPA6	7906764	1	83.1 ± 31.5	131.5 ± 72.9	48.4	0.0017	0.033	0.059	0.045
HSPA9	8114455	5	1,818.0 ± 162.0	1,995.5 ± 178.1	177.5	0.0024	0.041	0.011	0.048
IQCB1	8089954	3	188.9 ± 46.3	218.6 ± 62.4	29.6	0.0024	0.041	0.028	0.005
JOSD1	8076128	22	883.1 ± 178.9	994.1 ± 210.8	110.9	0.0012	0.027	0.016	0.025
LIPA	7934920	10	1,049.7 ± 191.0	919.0 ± 173.4	-130.7	0.0024	0.041	-0.015	0.040
LPCAT4	7987230	15	106.4 ± 7.3	92.8 ± 10.5	-13.6	0.0007	0.020	-0.013	0.049
MED10	8110886	5	352.3 ± 61.5	427.3 ± 71.2	75.0	0.0002	0.013	0.040	0.001
MIA2	7974080	14	86.9 ± 30.4	74.2 ± 29.0	-12.6	0.0024	0.041	-0.022	0.028
MYL6	7956211	12	4,630.9 ± 266.9	4,379.0 ± 339.0	-251.9	0.0002	0.013	-0.009	0.042
NEUROD1	8057480	2	1,106.1 ± 465.3	980.6 ± 426.5	-125.5	0.0007	0.020	-0.031	0.036
NR4A1	7955589	12	115.1 ± 22.4	137.1 ± 22.9	22.0	0.0002	0.013	0.035	0.002
NR4A2	8055952	2	90.7 ± 17.7	127.9 ± 32.0	37.2	0.0012	0.027	0.043	0.004
NR4A3	8156848	9	61.7 ± 5.0	76.0 ± 14.7	14.3	0.0002	0.013	0.022	0.016
PDZK1	7904843	1	224.1 ± 112.8	179.6 ± 97.6	-44.4	0.0024	0.041	-0.045	0.026
PECR	8058837	2	120.8 ± 22.0	97.6 ± 27.9	-23.3	0.0012	0.027	-0.022	0.028
PLIN2	8160297	9	392.4 ± 200.1	762.9 ± 577.3	370.6	0.0002	0.013	0.027	0.036
PPP1R15A	8030128	19	194.6 ± 36.2	319.2 ± 109.8	124.6	0.0002	0.013	0.033	0.004
PSMB7	8164067	9	1,373.3 ± 151.0	1,451.2 ± 140.5	77.9	0.0017	0.033	0.015	0.028
PXMP2	7960117	12	229.5 ± 35.5	205.4 ± 28.6	-24.2	0.0024	0.041	-0.023	0.006
RAB7L1	7923812	1	500.6 ± 113.7	457.6 ± 118.7	-43.0	0.0034	0.049	-0.021	0.025
RASGRP1	7987405	15	159.7 ± 54.0	138.0 ± 46.7	-21.6	0.0024	0.041	-0.043	0.010
RBM3	8167234	X	143.5 ± 36.4	164.0 ± 39.7	20.5	0.0005	0.017	0.031	0.038
SCD5	8101366	4	205.9 ± 57.3	179.7 ± 58.0	-26.3	0.0005	0.017	-0.019	0.028
SCP2	7901513	1	655.7 ± 107.4	594.7 ± 129.2	-61.0	0.0034	0.049	-0.012	0.043
SLC2A3	7960865	12	406.4 ± 106.3	664.2 ± 517.2	257.8	0.0002	0.013	0.030	0.036
SLC30A8	8148003	8	5,399.1 ± 1,886.6	4,762.5 ± 1,777.0	-636.6	0.0034	0.049	-0.031	0.019
SLCO2A1	8090823	3	61.4 ± 14.0	44.2 ± 4.5	-17.2	0.0012	0.027	-0.045	0.003

SNHG12	7914202	1	171.6 ± 75.1	267.0 ± 123.8	95.4	0.0034	0.049	0.035	0.024
SORD	7983393	15	568.3 ± 184.7	470.7 ± 152.8	-97.6	0.0024	0.041	-0.028	0.022
SRXN1	8064375	20	867.7 ± 225.9	1,159.6 ± 396.4	291.9	0.0012	0.027	0.023	0.029
SYNJ2BP	7979916	14	565.0 ± 47.9	501.3 ± 62.2	-63.7	0.0034	0.049	-0.014	0.040
TM4SF4	8083301	3	4,704.1 ± 1,740.2	3,956.5 ± 1,856.2	-747.7	0.0002	0.013	-0.054	0.003
TMEM54	7914592	1	434.0 ± 93.5	364.5 ± 82.6	-69.6	0.0002	0.013	-0.017	0.042
TMEM63B	8119926	6	168.6 ± 21.2	149.1 ± 19.3	-19.4	0.0034	0.049	-0.015	0.019
TNFRSF10B	8149733	8	321.5 ± 97.5	367.1 ± 139.2	45.6	0.0007	0.020	0.029	0.013
UFM1	7968670	13	1,058.4 ± 117.1	1,229.9 ± 142.0	171.5	0.0017	0.033	0.012	0.018
UGT2B15	8100737	4	2,339.8 ± 1,324.2	1,394.2 ± 1,331.7	-945.6	0.0007	0.020	-0.076	0.037
UGT2B15	8100746	4	2,339.8 ± 1,324.2	1,394.2 ± 1,331.7	-945.6	0.0007	0.020	-0.076	0.037
UGT2B7	8100758	4	2,820.4 ± 1,716.6	1,682.2 ± 1,628.9	-1,138.2	0.0007	0.020	-0.077	0.033
WDR44	8169519	X	208.8 ± 38.1	195.2 ± 41.1	-13.6	0.0034	0.049	-0.013	0.040
WEE1	7938348	11	133.2 ± 24.9	155.1 ± 31.4	21.9	0.0017	0.033	0.018	0.042
VIL1	8048319	2	137.9 ± 42.8	110.6 ± 43.4	-27.3	0.0024	0.041	-0.037	0.027
ZFAND2A	8137709	7	174.8 ± 30.4	208.7 ± 27.0	33.8	0.0005	0.017	0.036	0.045
ZNF165	8117630	6	107.7 ± 22.1	180.5 ± 46.4	72.8	0.0002	0.013	0.026	0.011
ZNFX1-AS1	8063337	20	512.3 ± 138.3	659.0 ± 177.7	146.7	0.0002	0.013	0.021	0.008
